# Reinventing Chemiluminescence through Redox-Driven Self-Assembly

**DOI:** 10.1021/jacs.5c12281

**Published:** 2025-10-14

**Authors:** Dario Alessi, Luca Morgan, Elisa Pelorosso, Mirco Scaccaglia, Piermaria Pinter, Alessandro Aliprandi

**Affiliations:** † Dipartimento di Scienze Chimiche, Università Degli Studi di Padova, Via Marzolo 1, Padova 35131, Italy; ‡ 243547 Novaled GmbH, Elisabeth-Boer-Straße 9, Dresden 01099, Germany

## Abstract

Chemiluminescence (CL) typically derives from harvesting the energy released during the cleavage of covalent bonds, wherein molecular substrates are irreversibly consumed to generate electronically excited states. In contrast, regenerative CL seeks to bypass substrate decomposition by using reversible redox processes to produce emissive states, an approach that is largely limited to polypyridyl complexes of Ru­(II) and Cr­(III). Here, we report a fundamentally distinct chemiluminescence mechanism in which the exergonic reduction of Pt­(IV) complexes powers the spontaneous formation of emissive Pt­(II) aggregates. Notably, light emission arises not from discrete molecular species but from the self-assembled state, whose electronic structure features a lowered excited-state energy (*E*_0,0_) conducive to radiative decay. The emission spectrum observed during chemical reduction matches that of photoexcited aggregates, confirming that the emissive state is intrinsic to the aggregated Pt­(II) architecture. This chemiluminescence occurs without external photoexcitation, and its intensity and persistence depend on the nature of the reductant, ranging from transient flashes to sustained afterglow. These findings unveil a new energy transduction pathway in CL: emission driven by redox-triggered self-assembly, expanding the conceptual and chemical space of chemiluminescence beyond classical molecular luminophores and toward dynamic, structure-responsive materials.

## Graphical abstract

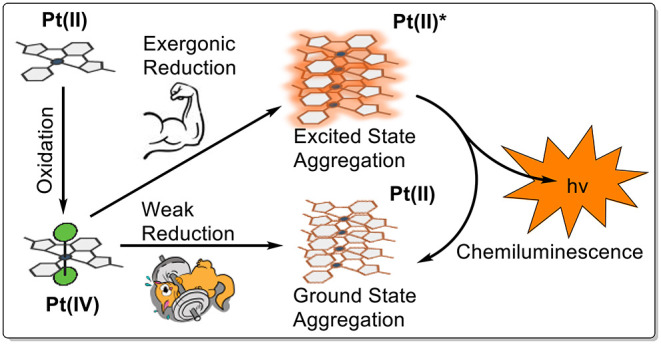

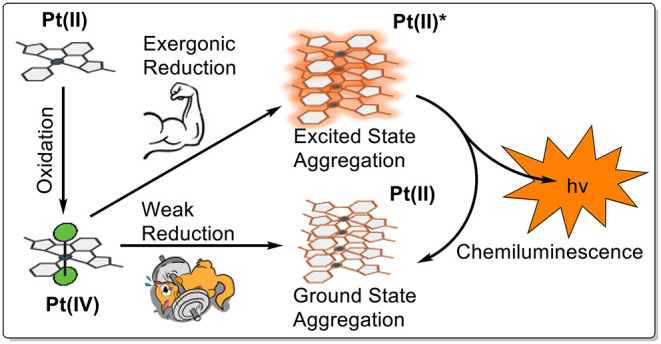

## Introduction

Electronically excited states of transition-metal complexes (TMCs) have long played a pivotal role in light-driven redox chemistry, most notably in the conversion of solar energy into chemical energy.^1,2^ In contrast, the reverse process, generating excited states directly from chemical energy via electron transfer, has received little attention. To the best of our knowledge, only a few early studies, notably by Hercules,^3^ Bard,^4^ and Balzani,^5^ have reported luminescence arising from such redox-triggered excitation, including the oxidation of Cr­(II) polypyridine complexes by persulfate to form Cr­(III)* and the reduction of Ru­(III) polypyridine complexes by hydroxide ions or oxalate to yield Ru­(II)*. Additionally, Ru­(III) species have been used as catalytic centers in the Belousov–Zhabotinsky reaction, resulting in oscillating chemiluminescence.^6^ These examples differ fundamentally from traditional chemiluminescence, in which light emission arises from highly exergonic reactions that cleave covalent bonds to populate excited states.^7^ In contrast, redox-triggered processes are characterized by a distinct three-step pathway. To align with our findings, the mechanism of redox-triggered chemiluminescence with TMCs will be elucidated through an oxidation–reduction pathway, as exemplified by Hercules with [Ru­(bpy)_3_]^2+^.^3^ Nonetheless, the reverse reduction–oxidation pathway is also viable.^5,8^ The initial phase of oxidation–reduction chemiluminescence involves the oxidation of a metal complex (M) with an electron acceptor to produce an analogous complex with M^*n*+^. Chemiexcitation subsequently occurs due to an exergonic electron transfer reaction between M^*n*+^ and an electron donor, culminating in the formation of the M complex in its excited state, M*. In the final step, M* returns to its ground state by emitting a photon, thus, regenerating the initial species.

$$M+EA\to M^{n+}+{EA}^{n-}$$

$$M^{n+}+ED\to M^{*}+{ED}^{n+}$$

$$M^{*}\to M+h\upsilon$$

This regenerative mechanism permits repeated excitation without chemical degradation of the luminophore, a concept termed regenerative chemiluminescence (RCL).^9^ The molecular components capable of mediating this energy transduction are termed chemiluminescence inducers (CLIs), which must combine suitable redox and photophysical properties to convert chemical potential into luminescence. The energetic feasibility of such redox-induced excitation for a generic luminophore (A) can be approximated by the relationship

1 E°(An*/An+1)≅E°(An/An+1)−E0,0

where *E*°(A^*n*^/A^*n*+1^) is the ground-state redox potential and *E*_0,0_ is the 0–0 transition energy of the excited state.^9^ From this relationship, it follows that lowering *E*_0,0_ reduces the redox energy required to generate an excited state, potentially enabling the use of mild, even biologically relevant, reductants such as NADH.

One promising strategy for achieving this goal involves the unique photophysical behavior of luminescent Pt­(II) complexes. Their square-planar geometry promotes intermolecular Pt···Pt interactions and π–π stacking between chromophores, which give rise to new orbitals through d*z* ^2^ orbital overlap and the formation of metal–metal-to-ligand charge transfer (MMLCT) excited states (Figure 1a,b).^10–13^ These metallophilic interactions, typically at distances of 3.0–3.5 Å, induce bathochromic shifts in absorption and emission spectra and effectively lower *E*_0,0_. Pt­(II) complexes bearing N^N^N-type chromophores and pyridinic ancillary ligands have been shown to self-assemble into supramolecular aggregates that exhibit large red-shifts in emission (from blue to red) and dramatically enhanced photoluminescent quantum yields (PLQYs) of up to 90%.^14–17^ Notably, a parameter of paramount importance for controlling the aggregation properties is the solvent/nonsolvent balance and the specific nature of the medium employed.^18^ In the field of Pt­(II) aggregation, the solvent is typically an organic medium (e.g., ACN, acetone, DMF, THF, 1,4-dioxane), which must be miscible with the countersolvent, most often H_2_O in the case of neutral Pt­(II) species. The solvent ratio can play a decisive role in driving the aggregation process, influencing not only the onset and kinetics of self-assembly but also the aggregate morphology, the photophysical properties, and the thermodynamic vs kinetic nature of the assembled state.^15,18–21^

**Figure 1:**
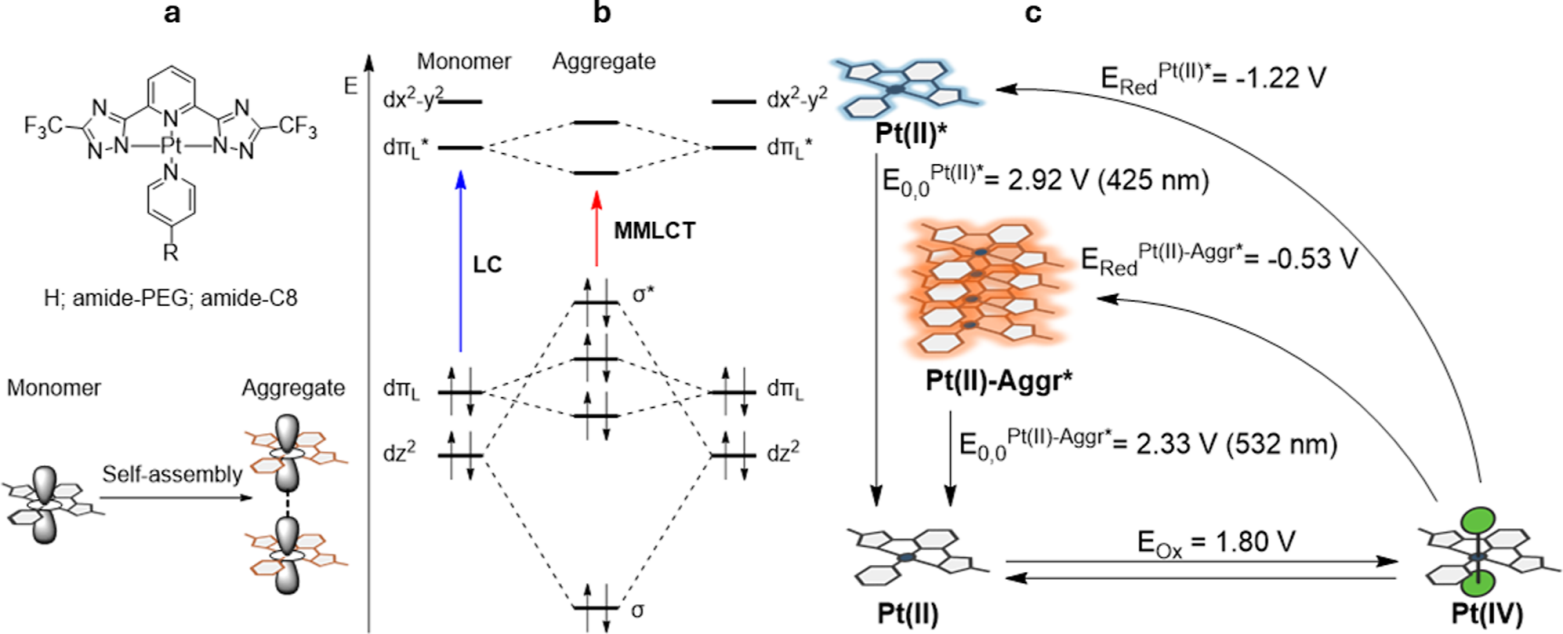
Molecular orbital evolution and chemiluminescence thermodynamics. (a) Schematic illustration of the effect of Pt­(II) aggregation on molecular orbital energies and electronic transitions. Aggregation leads to Pt···Pt interactions and π–π stacking, giving rise to new delocalized orbitals and lowering the energy of the emissive excited state (*E*_0,0_). (b) Thermodynamic diagram of the chemiluminescence process, depicting the redox-driven formation of the excited state in both monomeric and aggregated Pt­(II) species. The excitation energy (Δ*E* ≈ *E*_0,0_) required to populate the emissive state decreases upon aggregation, enabling light emission from redox events with lower driving force. Thermodynamic values are derived from experimental and computational data for complexes **1**, **1a**, and **A1**, the latter obtained via reduction of **1a** with sodium ascorbate.

Recently, we demonstrated that oxidation to an octahedral Pt­(IV) complex disrupts these noncovalent interactions, effectively halting self-assembly. Upon chemical reduction, the square-planar Pt­(II) geometry is restored, reactivating self-assembly pathways and yielding luminescent gels that cannot be directly accessed from the Pt­(II) precursor alone.^22^

In this study, we leverage this redox-triggered assembly process to achieve chemiluminescence driven by reduction reactions using mild reductants (Figure 1c). By coupling spontaneous self-assembly to a thermodynamically favorable redox event, we lower the excited-state energy through Pt···Pt interactions and enable light emission from the resulting supramolecular aggregates. In this work, we present chemiluminescence arising from the formation of self-assembled excited-state structures, a strategy that allows for CL activation with biologically compatible reductants such as NADH. This redox-assembly emission mechanism opens new directions for chemiluminescence in bioimaging, chemical sensing, and adaptive supramolecular materials.

## Results and Discussion

### Synthesis and Characterization

The Pt­(II) precursors bearing various pyridine-based ligands were oxidized using PhICl_2_ in refluxing ethyl acetate to yield the corresponding Pt­(IV) complexes. Pt­(II) complexes bearing a simple pyridine ligand (**1**), a pyridine functionalized with an amide-PEG chain (**2**), or a pyridine appended with an eight-carbon aliphatic chain (**3**) were synthesized according to the reported procedures.^23^ The Pt­(II) species were oxidized to their Pt­(IV) analogues (**1a–3a**), as shown in Scheme 1. Oxidation was performed in refluxing ethyl acetate with a 2-fold excess of PhICl_2_ over 1–3 h. During the reaction, the initially yellow heterogeneous slurry gradually became a pale-yellow homogeneous solution. The Pt­(IV) complexes were purified by silica gel chromatography using mixtures of dichloromethane (or hexane) and ethyl acetate. The products (**1a–3a**) were isolated as pale-yellow solids in 60–70% yield. NMR analysis in CDCl_3_ (see Figures S1–S16; HR-MS Figures S17–S19) confirmed the formation of the Pt­(IV) complexes. Notably, protons at positions 2 and 6 of the ancillary pyridine ligands exhibited symmetrical coupling to the ^195^Pt nucleus, resulting in satellite peaks with ^3^ *J*_H–Pt_ coupling constants ranging from 11.6 to 11.9 Hz, consistent with the Pt­(IV) oxidation state. The structural assignment was further corroborated by single-crystal X-ray diffraction analysis reported previously.^22^ The solubility of Pt complexes is crucial for selecting solvents that facilitate rapid and efficient aggregation because a portion of the exergonic energy released during the reduction of Pt­(IV) to Pt­(II) must be transferred to the self-assembled structures to generate the excited states. Pt­(II) complexes generally exhibit low solubility due to their square-planar geometry, which promotes strong noncovalent interactions such as π–π stacking and hydrogen bonding. On the other end, Pt­(IV) complexes, owing to their additional axial ligands, typically display enhanced solubility relative to their Pt­(II) precursors. Complexes **1** and **2** show limited solubility in most solvents, with the notable exception of *N*,*N*-dimethylformamide (DMF). The amide functionality presents in the pyridine ligands of **2** and **3** introduces additional hydrogen-bonding capabilities, which are often critical for achieving higher supramolecular entities such as metallogels.^24–26^ The appended substituents are responsible for the markedly different solubility profiles of the Pt­(II) complexes. Compound **2** is soluble in dichloromethane (DCM), dimethyl sulfoxide (DMSO), and tetrahydrofuran (THF), slightly soluble in dioxane, and insoluble in more polar solvents such as acetonitrile (ACN), methanol (MeOH), and water. In contrast, compound **3**, bearing an eight-carbon aliphatic chain, exhibits the highest solubility across the series, dissolving readily in nearly all organic solvents, including ACN, but remains poorly soluble in MeOH and water. Notably, the general insolubility of these Pt­(II) complexes in water facilitates rapid self-assembly into aggregates upon addition of a poor solvent. Compounds **1a**–**3a** are soluble in hot ACN and moderately soluble in less polar solvents such as ethyl acetate while remaining insoluble in alkanes and water.

**Scheme 1:**
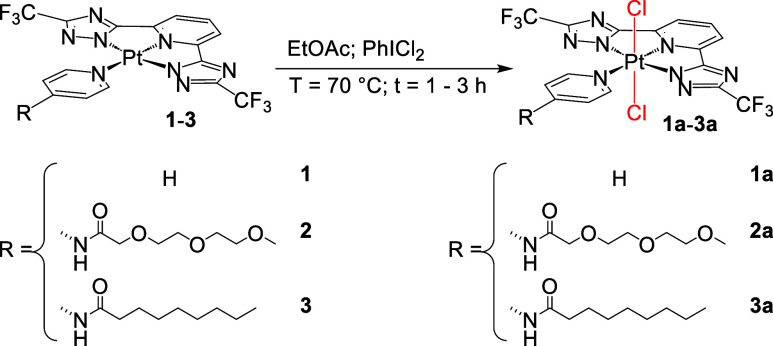
Oxidation of Pt­(II) Complexes (**1**–**3**) to Pt­(IV) Analogues (**1a–3a**)

### Chemiluminescence

Chemiluminescence from the self-assembled Pt­(II) structures was triggered by the addition of a reducing agent. To investigate this phenomenon, stock solutions of Pt­(IV) complexes (**1a**–**3a**) were prepared by dissolving the compounds in hot acetonitrile (70 °C), followed by cooling to room temperature to maximize the solubility (final concentration: 0.02 M). A series of reducing agents with varying redox potentials, including sodium borohydride (NaBH_4_), sodium ascorbate (NaAsc), nicotinamide-adenine dinucleotide reduced (NADH), and glutathione (GSH), were evaluated. Chemiluminescence measurements were performed by placing a 2 mL vial containing 100 μL of Pt­(IV) solution directly above a photomultiplier tube (PMT) or in a custom sample holder coupled to a spectrometer equipped with a Charge-Coupled Device (CCD) via an optical fiber (see the Methods section). A larger quantity of the reducing agent (500 μL, 0.04 M in 0.1 M NaOH, pH 13) was injected into the vial, triggering in situ reduction of Pt­(IV) to Pt­(II) and formation of supramolecular aggregates (**A1**–**A3**), accompanied by light emission (Figures 2 and 3 and Table 1). The higher concentration and volume of the aq reducing agent solution serves a double purpose: it allows for quick reduction of Pt­(IV) compounds and simultaneously favors the aggregation of the Pt­(II) compound, respectively. Notably, the pH was identified as a critical parameter controlling chemiluminescence: no emission was observed under neutral or acidic conditions. Although NaOH alone sufficed to slowly reduce Pt­(IV) to Pt­(II) and induce aggregation, it did not produce a light emission. This likely reflects the pH-dependent reducing power of the agents, consistent with prior reports on sodium ascorbate’s oxidation potential,^27^ or the stability in aqueous solution such as in the case of NaBH_4_.

**Figure 2:**
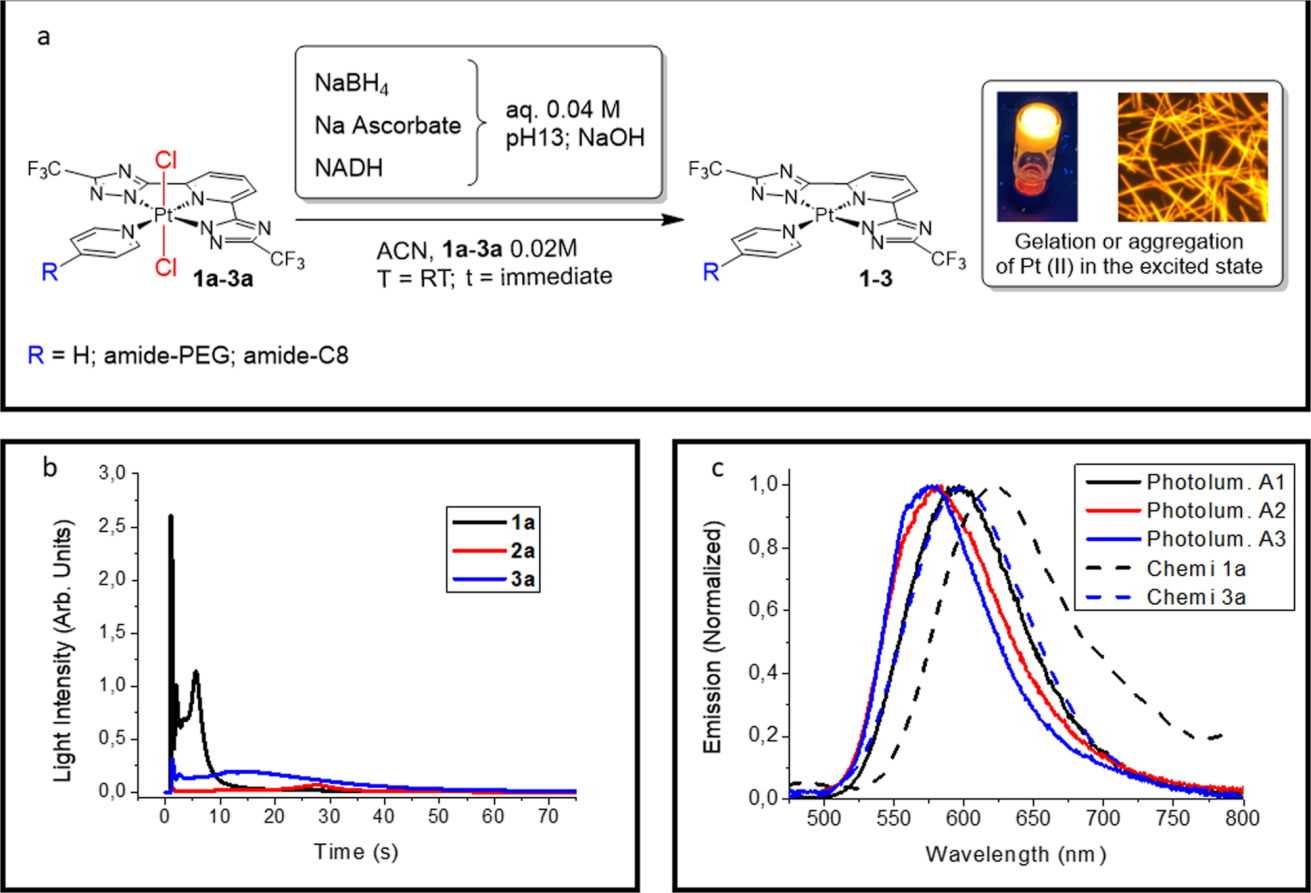
Chemiluminescence experiments with compounds **1a**–**3a** and reducing agents. (a) Schematic representation of the experimental steps. (b) Overlaid chemiluminescence intensity profiles (measured by photomultiplier tube, PMT) for reactions between compounds **1a**–**3a** (0.02 M in acetonitrile, 100 μL) and NaBH_4_ (0.04 M in water, 500 μL). (c) Overlay of normalized photoluminescence of compounds **1a**–**3a** (solid lines) and chemiluminescence emission spectra, acquired via CCD camera, obtained by reactions described at point **b** (dashed lines). Chemi **2a** not applicable.

**Figure 3:**
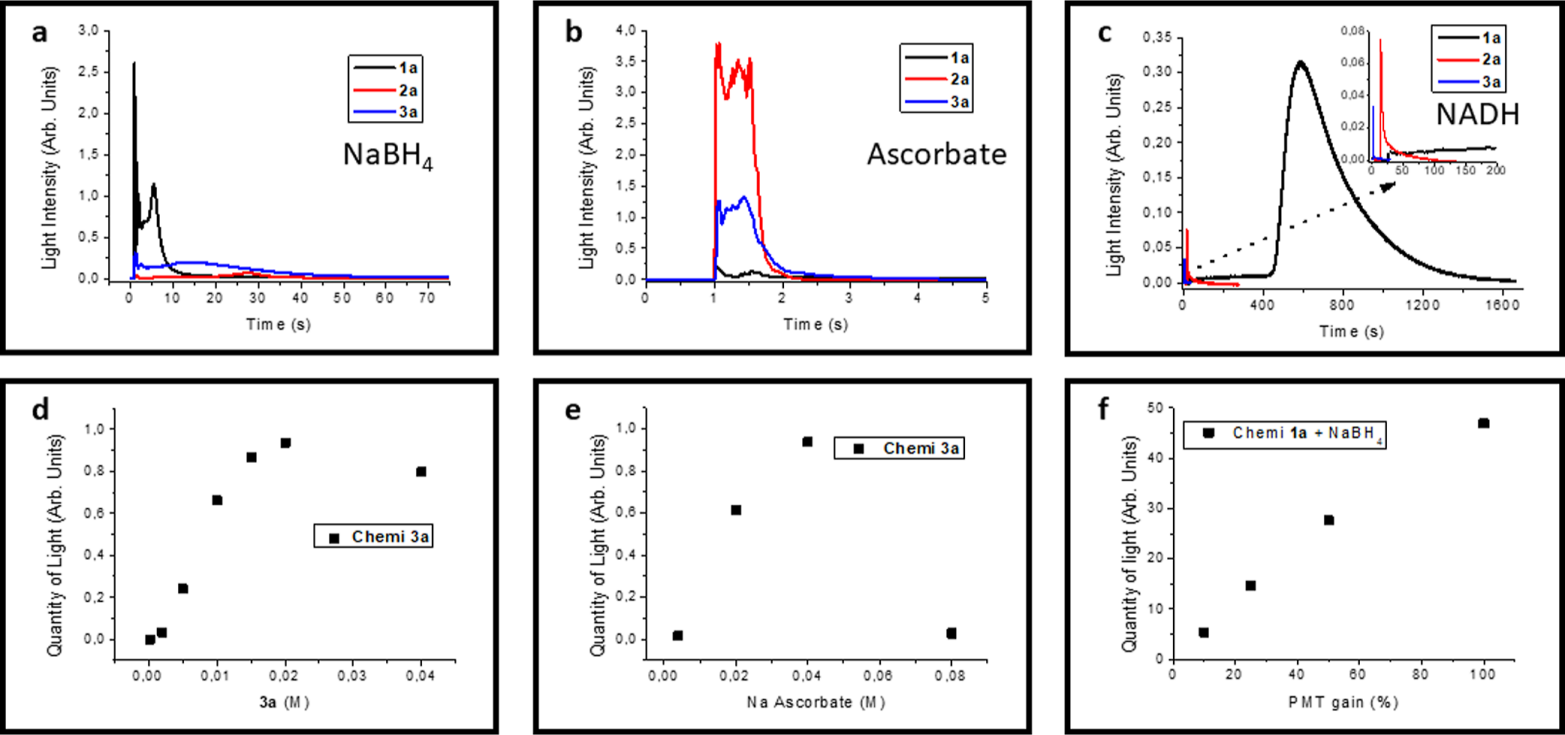
Chemiluminescence profiles and parameter studies. (a–c) PMT-measured chemiluminescence signals from reactions between complexes **1a**–**3a** (0.02 M in acetonitrile, 100 μL) and aqueous reducing agents (0.04 M, 500 μL): (a) NaBH_4_, gain 25; (b) sodium ascorbate, gain 10; and (c) NADH, gain 100; (d) effect of complex concentration on chemiluminescence intensity using sodium ascorbate as the reducing agent. (e) Influence of reducing agent concentration on chemiluminescence intensity using **3a** as the benchmark complex. (f) PMT signal linearity as a function of gain, measured using the chemiluminescence reaction between **1a** and NaBH_4_.

**Table 1:** Chemiluminescence Performances and Photophysical Properties of Aggregates^a^

entry	red. agent (GAIN %)^b^	pH	Pt(IV) complex	emitted light^d^ (a.u.)	λ_max,emi_ ^e^ (nm)	*E*_0,0_ (eV). (λ nm)	PLQY (%)
1	**NaBH**_**4**_ **(25)**	13	**1a**	6.25	605	2.37; (523)	10
2			**2a**	1.21	581	2.42; (512)	57
3			**3a**	6.42	582	2.40; (517)	35
5	**ascorbate (10)**	13	**1a**	0.34	595	2.33; (532)	30
6			**2a**	2.10	590	2.40; (516)	56
7			**3a**	0.94	577	2.38; (521)	13
9	**ascorbate** ^c^ **(10)**	7	**1a**				65
10			**2a**				83
11			**3a**				34
13	**NADH (100)**	13	**1a**	114.47	588	2.39; (518)	60
14			**2a**	0.19	591	2.41; (514)	65
15			**3a**	0.05	597	2.37; (523)	8

a All chemiluminescent experiments in the table are performed by flash injection of reducing agent solution (500 μL, 0.04 M, pH 13 NaOH, aq) into Pt­(IV) complex solution (100 μL, 0.02 M, ACN).

b The gain of the PMT detector.

c With no additional base.

d Total light output quantified by integrating the PMT signal over time.

e Spectroscopic data obtained from the luminescent aggregates following reduction.

At lower pH values, excited-state formation appears suppressed, whereas at very high pH values (e.g., pH 14), immiscibility between the reducing mixture and acetonitrile may impede mixing and reaction kinetics. To further investigate if the pH is the dominating factor, rather than the ionic strength of the solution, control experiments were carried out using **3a** as benchmark compound (0.02 M, ACN) and by substituting the 0.1 M NaOH with an equal concentration solution of NaCl containing the reducing agent (NaAsc 0.04 M). The experiment revealed no light output at a maximum instrumental sensitivity. Then, to test if the ionic strength plays a role in satisfying the condition for CL alongside the pH, the ascorbate reducing solution was set at pH 12, maintaining the same ionic strength as the solution at pH 13 by the addition of NaCl. This time, the signal was barely recognizable from the noise. A control experiment using the ascorbate solution at pH 12 with no ionic strength compensation has been carried out and gave the same outcome of the previous one, definitively ruling out the hypothesis of a role of ionic strength in the aggregation-induced CL see Figure S20. As a comparison, the signal registered in the same condition at pH 13 and with lower gain (10) was 4 orders of magnitude greater than that at pH 12.

The reduction of compound **1a** with NaBH_4_ in basic conditions (pH 13) rapidly yields the corresponding Pt­(II) complex **1**, which self-assembles into the jelly like orange material **A1**-**NaBH**_**4**_. Dynamics of gelation and thermodynamic vs kinetic aggregation has already studied by our group.^22,23^ Notably, gel formation is accompanied by chemiluminescence from the aggregates (Figure 2b, 3a). The chemiluminescence emission spectrum closely overlaps with the photoluminescence spectrum of aggregated **1** (Figure 2c), confirming the assembled material as the light source. The fluctuating light emission reflects the heterogeneous, gel-like nature of the assembly. In particular, the absence of stirring leads to the local accumulation of reagents in different regions of the mixture, which results in an uneven emission profile.

Complexes **2a** and **3a** showed chemiluminescent behavior, upon NaBH_4_ treatment (pH 13), that rapidly formed the corresponding Pt­(II) complexes as granular aggregates: **A2-NaBH**_**4**_ and **A3-NaBH**_**4**_ (Figure 2b, 3a). Chemiluminescence spectrum from **3a** matched its aggregate photoluminescence (Figure 2c), while **2a** emission was too weak for CCD spectral acquisition. The aggregate-based emission origin was confirmed as was the case for **1a**. Chemiluminescence from **2a** decayed smoothly, unlike the fluctuating emission of **1a**. Compound **3a** behaved similar to **1a** but with a lower peak intensity and longer duration (∼70 s). Total light emission followed the trend: **1a** ≅ **3a** > **2a**, with **2a** emitting approximately 25% of **3a**′s total, despite having the highest PLQY among NaBH_4_-derived aggregates (Figure 2b, **3a**; Table 1).

Replacing NaBH_4_ with sodium ascorbate produced similar aggregates but with shorter emission durations. **A2**-**Asc** exhibited a bright flash that approached the saturation limit of the detector, yielding integrated intensities 2–6 times higher than those of the other compounds (Figure 3b). To ensure comparability among samples, CL was measured at the same instrumental sensibility (photomultiplier gain set to 10), a compromise that allowed all traces to be visualized while minimizing the loss of the integrated area from **2a**, despite its near-saturating signal. As before, chemiluminescence spectra matched aggregate photoluminescence (Figures S21). **A1-Asc** and **A3-Asc** provided strong but less intense light output lasting 1–2 s, precluding spectral recording. With ascorbate, light output increased in the order **1a** < **3a** < **2a**, opposite to NaBH_4_.

Reduction of **1a** with NADH (PMT gain 100) showed a 400 s induction period with low light emission, followed by 20 min of considerable light development (Figure 3c). Conversely, **2a** and **3a** produced only weak, brief signals with NADH.

The optimal luminophore and reductant concentrations were established through chemiluminescence experiments. For this set of studies, compound **3a** and basic sodium ascorbate solution were chosen as the benchmark pair because they provide a sharp and intense CL signal without saturating the detector under all conditions. The luminophore concentration was varied in the range 0.002 to 0.04 M (100 μL in ACN), while a fixed amount of reductant solution (0.04 M, 500 μL in H_2_O pH 13) was added. From these experiments, the optimal luminophore concentration was identified as 0.02 M (Figure 3d). Similarly, the optimal reductant concentration was determined by varying the sodium ascorbate concentration in the range 0.004–0.08 M (aq. pH 13, 500 μL), while maintaining compound **3a** at 0.02 M (100 μL in ACN). Under these conditions, the optimal reductant concentration was found to be 0.04 M (Figure 3e). To allow a quantitative comparison of CL signals recorded at different PMT gain levels, the relationship between signal intensity and gain was examined. For this purpose, the redox pair compound **1a**/NaBH_4_ was employed as it provides the largest integrated CL area without saturating the detector even at maximum sensitivity. The response showed a linear trend, thereby confirming the comparability of measurements after signal conversion across gain settings (Figure 3f).

The regenerative nature of our Pt­(IV)/Pt­(II) system has been previously demonstrated by NMR spectroscopy on the **1a**/**1** couple, as reported in detail in our recent work.^22^ In this study, we further substantiated this property under the conditions employed for chemiluminescence. After the CL reactions, the Pt­(II) complexes could be separated from residual salts by straightforward washing with H_2_O and filtration and subsequently reoxidized to their Pt­(IV) precursors as described in the experimental procedure (see the Methods section). This recycling process was repeated at least ten times without an appreciable loss of performance. Moreover, the aggregates **A3** formed during the CL process were monitored by emission spectroscopy for up to 7 h during which the spectral profiles remained unchanged (see Figure S22). This underscores the robustness and regenerative capacity of the system.

To place the aggregation-assisted CL system in the broader context of chemiluminescence research, we compared it with one of the most established and best-performing redox chemiluminescence systems, the [Ru­(II)­bpy_3_]^2+^/[Ru­(III)­bpy_3_]^3+^ couple. Although both rely on redox-driven processes, the Ru-based system differs fundamentally from ours in several respects. First, the Ru system does not involve an aggregation step; instead, CL arises directly from the radiative decay of Ru­(II), which is intrinsically emissive. Second, generation of the active Ru­(III) species requires harsh conditions, namely, 9 M H_2_SO_4_ and PbO_2_ as oxidants, and the resulting species cannot be transferred out of the strongly acidic medium (see the Methods section for experimental details). For comparison, we examined the Ru system under two sets of reducing conditions: (i) the same NaAsc/basic mixture employed for the Pt-based CL reported herein (NaAsc 0.04 M pH 13), and (ii) the conditions originally described by Hercules with addition of NaAsc (NaAsc 0.04 M in 9 M NaOH).^3^ Under condition (i), no detectable emission was observed even at maximum detector sensitivity. In contrast, condition ii yielded an extremely intense signal that saturated the PMT at minimum sensitivity, with emission lasting for the entire duration of the experiment. These results highlight key differences between the two systems. The Ru-based CL is far more intense but it requires extreme acidic/basic conditions and toxic oxidants. The Pt aggregation-based CL, in contrast, operates under comparatively milder conditions, albeit with lower overall intensity. Importantly, the Pt­(II) monomer is nonemissive, and luminescence emerges only upon aggregation, thus enabling CL from precursors that are themselves nonluminescent. This unique feature distinguishes aggregation-based CL as a complementary strategy to classical Ru­(bpy)-type systems.

### Thermodynamic and Mechanistic Investigation

We performed electrochemical, photophysical, and computational studies to elucidate the origin of the light emission during the chemiluminescent processes and clarify if the chemiluminescence mechanism involves only monomeric or aggregated Pt­(II) species. We first determined the oxidation potentials of the monomeric Pt­(II) species through cyclic voltammetry (CV) experiments conducted in acetonitrile (ACN) (see the Methods section for experimental details). Compounds **1**–**3** were analyzed at a concentration of 10^–3^ M; however, despite the dilution, they still formed small amounts of aggregates (**A1**-**A3**). The CV profile of compound **1** (see Figure S23) reveals a reversible reduction process at −1.28 V, attributed to the reduction of the terdentate ligand, along with two broad irreversible oxidation events assigned to the oxidation of the Pt center at positive voltages. The first Pt­(II)-centered oxidation appears as a very broad and weak signal around +1.0 V, likely due to Pt···Pt metallophilic interactions, which destabilize the HOMO orbital. A more intense irreversible oxidation is observed at +1.80 V, corresponding to the monomeric species in the absence of such interactions. Compound **2**, despite containing a PEG substituent, displayed poor solubility in the polar solvent ACN and, like compound **1**, formed small amounts of aggregate **A2**. Its CV (see Figure S24) exhibited a quasi-reversible reduction at −1.30 V and a quasi-irreversible peak centered at −1.57 V, attributed to the py-PEG ligand. As in the case of compound **1**, CV also displayed a weak and broad irreversible oxidation signal around +1.0 V, which is attributed to the presence of **A2** aggregates. The second more intense oxidation peak was observed at +1.76 V, corresponding to fully dissolved complex **2**. Attempts to inhibit the aggregation signal has been carried out by addition of TWEEN 20 surfactant at 5 to 20% v/v but proves to be ineffective strategy. To unambiguously attribute the source of the signal at +1.0 V, ECL experiments have been conducted on **A2** (10^–3^ M) using a DMF/H_2_O (2:1) solvent mixture, sodium ascorbate (5 × 10^–3^ M, pH 13, NaOH) as the reducing agent (coreactant), and TBAPF_6_ (0.1 M) as the supporting electrolyte (see Figure S25). In principle, ECL may originate either from aggregated species,^28,29^ or from monomeric Pt­(II) complexes,^30^ in the presence of a suitable coreactant. However, since the monomeric forms of the complexes investigated in this study are intrinsically nonemissive, any observed emission must be attributed to the aggregates. Indeed, emission was observed during the first oxidation process, thus supporting the assignment of the +1.0 V signal. On the same line, the experiment was carried out in a reduction–oxidation scan and using PhICl_2_ as the oxidating agent without additional base to intercept the ECL signal in correspondence of the reduction potential of the aggregates (see Figure S26). Accordingly with this experiment, the onset of ECL is at −1.1 V. The mild shift in reduction potential and the great shift of the oxidation potential reflect the magnitude of stabilization of the LUMO orbital and the great destabilization of HOMO imposed by metallophilic interactions. In contrast, compound **3**, bearing a C_8_ aliphatic chain, exhibited good solubility in ACN. Its CV (see Figure S27) showed a reversible reduction at −1.29 V, assigned to the terdentate ligand, and a single irreversible oxidation at +1.70 V. No signals associated with aggregate formation were detected.

Compounds **1**–**3**, fully dissolved in acetonitrile (10^–4^ M), exhibit weak absorption in the visible region, primarily due to spin-allowed metal-to-ligand charge transfer (^1^MLCT) and intraligand (^1^IL) transitions between 438 and 425 nm (2.92–2.83 eV), with λ_abs,max_ values ranging from 395 to 391 nm. These are followed by more intense absorption bands in the UV region, predominantly attributed to ^1^IL and metal-perturbed interligand charge transfer (^1^ILCT) transitions (see Figure S28). In their monomeric form, Pt­(II) complexes are generally nonemissive, with the exception of complex **2**, which displays weak and structured blue emission peaks at 390, 412, 436, 460, and 490 nm (see Figure S29). Oxidation of Pt­(II) complexes **1**–**3** with PhICl_2_ yields pseudo-octahedral Pt­(IV) species (**1a**–**3a**) featuring axial chloride ligands. These Pt­(IV) derivatives are nonluminescent and display a blue-shifted absorption onset (∼386 nm) compared to their Pt­(II) counterparts, with local maxima at 355 and 344 nm (see Figure S30). They appear pale yellow in solution (10^–4^ M in ACN).

Chemical reduction of the Pt­(IV) complexes restores the parent Pt­(II) species, which rapidly self-assemble into highly luminescent supramolecular aggregates (**A1**–**A3**) driven by Pt···Pt metallophilic interactions.^22^ The resulting aggregates display structured absorption spectra extending further into the visible region, with λ_abs,max_ around 485 nm, characteristic of ^1^MMLCT transitions (see Figure S31). Aggregates for photophysical analysis are generated by mixing equal volumes (75 μL each) of a 0.02 M solution of the Pt­(IV) complex in ACN and a 0.04 M aqueous sodium ascorbate solution (pH 13, adjusted with NaOH) in a 150 μL quartz cuvette.

Freshly formed aggregates (in situ reduction of **1a**–**3a** in ACN with 2 equiv of aqueous solution of ascorbate with no additional base) **A1**–**A3** are strongly photoluminescent, with PLQYs of 65%, 83%, and 34%, respectively, at pH 7 (see Figure S32; Table 1, entry 9–11) due to the formation of Pt···Pt interactions. Upon excitation at 470 nm, they emit in the orange-red region, with emission maxima between 580 and 595 nm. Notably, **A2** displays two different emissive species due to a more complex self-assembly landscape that has been previously described in detail (see Figure S33).^23^ Conversely, the aggregates obtained after the chemiluminescence reactions show slight differences in λ_max,em_ depending on the nature of the reducing agent employed. Thus, the maximum photoluminescence emission of such aggregates ranges between 577 and 605 nm, and their high-energy onset ranges from 512 to 527 nm (2.42–2.35 eV) (see Table 1). The PLQYs of aggregates **A1**–**A3** also varied significantly depending on the reducing agent used, with notable differences, for example, aggregates from **1a** exhibited an increase from 10% with NaBH_4_ to 60% with NADH. See Figures S34–S36 for emission spectra of the different combinations of aggregates and reducing agents at pH 13. Notably, PLQY is pH dependent decreasing substantially between samples formed at pH 7 and 13, namely: **A1** decreases from 65 to 30%, **A2** decreases from 83 to 56%, and **A3** decreases from 34 to 13%. Although the strong basicity of the system negatively affects the light output, it remains essential for chemiluminescence to occur.

### Morphological Analysis via Fluorescence Microscopy

Fluorescence microscopy was employed to examine the morphology of Pt­(II) aggregates formed during CL reactions. The samples were generated directly on a quartz Petri dish using one-fifth of the standard CL volumes, while maintaining the same concentrations and pH (see the Methods sections for details). Images of the resulting aggregates are reported in Figure S37 for each combination of complex and reducing agent. For compound **1a**, NaBH_4_ and NADH promote the formation of diffuse, cloudlike assemblies accompanied by partial development of needle-shaped structures, whereas sodium ascorbate produces exclusively the cloud-like morphology. Aggregates obtained from **2a** are consistently amorphous and flocculent across all reductants, and the presence of cloud-like aggregates is limited to NaBH_4_ reducing agent. The size of amorphous aggregates increases by decreasing the strength of the reducing agent employed, so they are thinnest when NaBH_4_ is employed and biggest with NADH. In the case of compound **3a**, more pronounced morphological differences are observed: NABH_4_ affords flocculent assemblies similar to those of **2a** with NADH, and sodium ascorbate yields distinct needle-like structures radiating from central nucleation points; NADH instead produces cloudy aggregates that tend to coalesce. Taken together, these observations show that the three complexes give rise to morphologies that vary both with the identity of the complex and the reductant employed. Moreover, a qualitative trend emerges when comparing aggregate appearance with CL intensity: more defined and structured morphologies are associated with stronger light emission within the same class of reductant employed, whereas ill-defined, cloud-like aggregates correspond to weaker signals.

### DFT Calculations

The minimum energy required to reach the excited state (eq 1) was estimated by electrochemical and photophysical analyses. For monomeric Pt­(II) complexes, the reduction potential necessary to trigger chemiluminescence is approximately −1.17 eV, while for the aggregates, it decreases to values between −0.50 and −0.70 eV due to the lower *E*_0,0_ caused by the establishment of Pt···Pt interactions. It is worth noting that the slight difference in the high-energy emission onset causes a small difference in *E*_0,0_. Consequently, NaBH_4_, with a standard oxidation potential of −1.24 eV, is more effective than sodium ascorbate in promoting chemiluminescence, which in turn is more effective than NADH, except for the abnormal behavior of compound **1a**.

To confirm the lower energy requirement for accessing the excited state in aggregates compared with monomers, we first examined the electronic properties of the ground-state monomer (GS_monomer) and the aggregate complexes using computational methods (Figure 4 for computed structures). Although the exact structure of the aggregates remains unknown, we recently reported the solid-state structure of a closely related Pt­(II) complex.^31^ Based on this precedent, we modeled the aggregates by constructing dimers by using structural parameters derived from the experimental structure. Herein, all of the investigated complexes are truncated in order to reduce the computational costs. All Pt–Pt interaction lengths and stabilization parameters of different dimers are compiled in Table S1.

**Figure 4:**
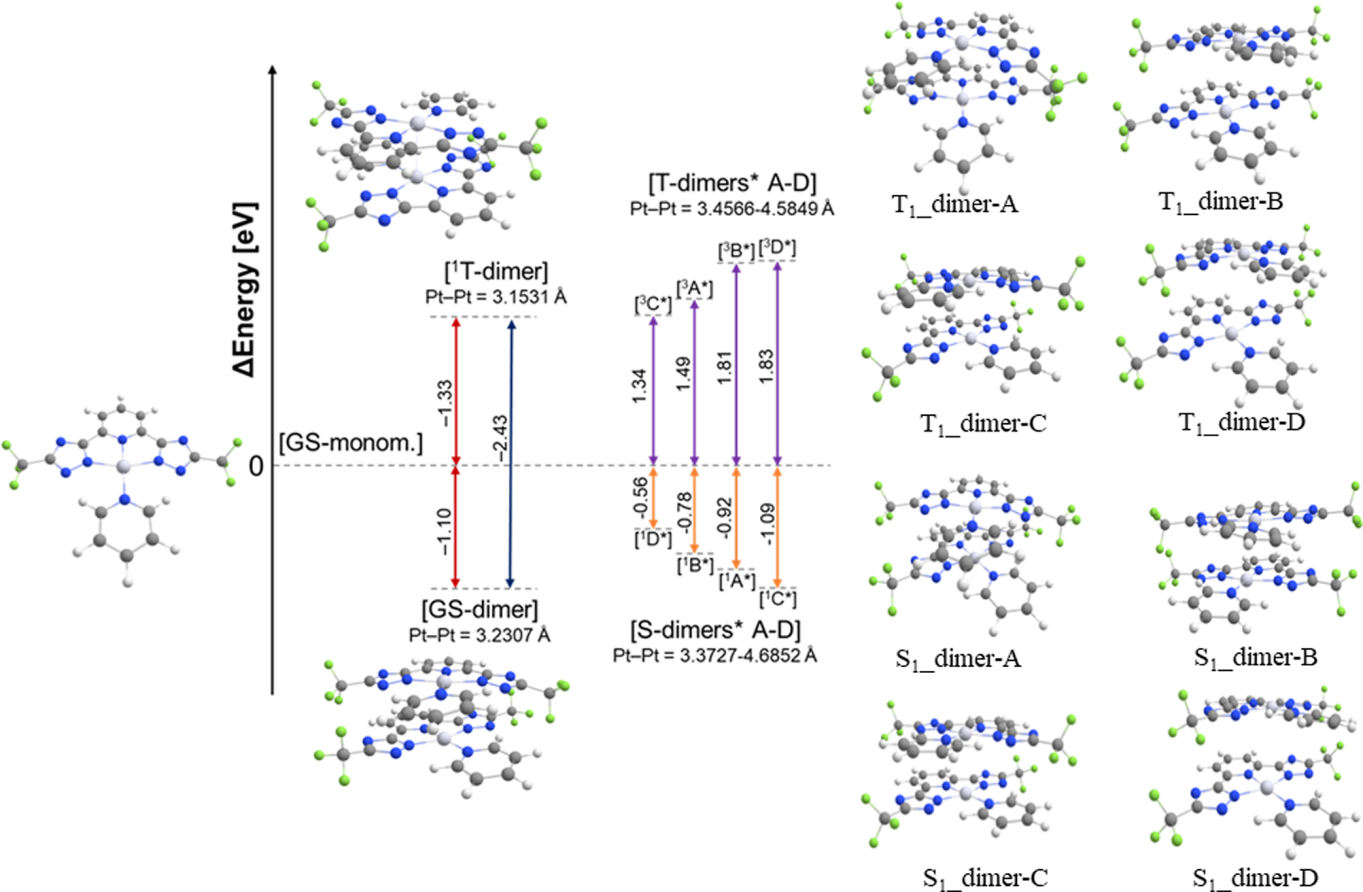
The calculated structures of the truncated molecule as monomer (GS-monomer), dimers in the singlet and triplet states (GS-dimer and ^1^T-dimer, respectively) and excited dimers in their triplet (T_1_-dimers A-D) and singlet (S_1_-dimers A-D) states. In the figure, the energies relative to the monomer in the ground state and the Pt–Pt distances are reported. The energies of the GS-monomer, GS-dimer, and ^1^T-dimer and excited state dimers (S_1_-dimers A-D and T_1_-dimers A-D) were obtained at the ZORA PBE0/ZORA-Def2-TZVPP with SARC-ZORA-TZVPP for platinum level of theory. For more details, see the DFT calculation part in the Methods section.

We optimized the geometry of a ground-state dimer (GS_dimer) featuring short Pt···Pt distances (Pt–Pt = 3.2307 Å) and calculated an energy stabilization of −1.10 eV relative to the GS_monomer. This result supports the well-established concept that metallophilic Pt···Pt interactions stabilize the aggregate formation. Consistent with experimental findings, the geometry of the dimer in its first triplet excited state (T_1__dimer, Pt–Pt = 3.1531 Å) shows further shortening of the Pt–Pt distance and a singlet–triplet energy gap of 2.4 eV. Next, we investigated the electronic properties of the dimers in their adiabatic singlet and triplet excited states. We optimized four different dimers in the first excited singlet state (S_dimer-A to S_dimer-D) and four in the first excited triplet state (T_dimer-A to T_dimer-D), each exhibiting different Pt–Pt distances (see Table S1) and calculated the energetic barrier between the ground and excited state dimers as the energy difference between the excited and ground state dimers referenced versus twice the energy of the monomer. We note that in all adiabatic excited-state dimers, the Pt–Pt distances increase relative to the GS_dimer, consistent with occupation of the antibonding Pt···Pt orbital (see Figure 1a).

Among the singlet excited dimers, S_dimer-C (Pt–Pt = 3.3739 Å) showed an almost negligible energy increase (+0.01 eV) relative to the GS_dimer. For S_dimer-A, -B, and -D, the calculated energy differences were +0.18, +0.32, and +0.54 eV, respectively. Notably, all adiabatic singlet excited dimers are still lower in energy than GS_monomer (−0.92, −0.78, −1.09, and −0.56 eV, respectively, for S_dimer-A, -B, -C, and -D), demonstrating that intermolecular interactions, including Pt···Pt metallophilic interactions, confer energetic stabilization to the aggregates, even in their excited states. As expected, the magnitude of stabilization correlates with the shorter Pt–Pt distances. A similar trend was observed for the triplet excited dimers. T_dimer-C (Pt–Pt = 3.4238 Å) exhibited a negligible stabilization energy difference of +0.01 eV from T_1__dimer, while T_dimer-A, -B, and -D displayed energy increases of +0.16, +0.48, and +0.50 eV, respectively.

## Conclusions

This study presents the first example of chemiluminescent self-assembly wherein the reduction of Pt­(IV) complexes induces the formation of supramolecular aggregates in the excited state. Unlike conventional chemiluminescence, which relies on covalent bond cleavage, our approach leverages electron transfer to regenerate the complexes, enabling continuous recycling and oxidation. The self-assembly of square planar luminescent Pt­(II) complexes effectively lowers the excited-state energy, facilitating the use of mild reducing agents, such as ascorbate. Crucially, our findings support the hypothesis that the chemiluminescence mechanism does not proceed through monomeric Pt­(II) species. This conclusion is based on several key observations. First, monomeric species, particularly compounds **1** and **3**, are essentially nonemissive in solution. Second, chemiluminescence onset potentials align with aggregate-related oxidation processes rather than monomer-centered redox events. Lastly, both photophysical and electrochemical signatures of light emission match those of preformed aggregates (**A1**–**A3**), not the monomeric species. These results strongly indicate that the aggregates and not the monomers are the emissive species involved in the chemiluminescent process. Attempts to directly correlate light output with the oxidation potential of the reducing agents revealed no consistent trend across the series, highlighting the multifactorial nature of the chemiluminescence process. This complexity warrants further investigation in future studies. Notably, the choice of reducing agent and pH critically influences the intensity and duration of chemiluminescence, with sodium ascorbate and NADH proving particularly effective in sustaining prolonged emission.

## Methods

### General

All of the reactions were carried out under an inert atmosphere of argon (Schlenk technique). All of the solvents and chemicals were used as received from Aldrich or Fluka without any further purification. The compounds were purified by column chromatography by using either silica gel 60 (70–230 mesh) or neutral alumina as the stationary phase. ^1^H, and ^13^C­{^1^H} spectra were recorded on a Bruker AVANCE III spectrometer equipped with a BBO probe, while ^19^F­{^1^H} NMR spectra were recorded with a Bruker 200 MHz instrument equipped with a QNP probe. ^1^H and ^13^C chemical shifts were referenced relative to residual solvents peaks while ^19^F chemical shifts were referenced using indirect referencing.^32^ The ^1^H NMR chemical shifts (δ) are given in ppm and refer to residual protons on the corresponding deuterated solvent. All coupling constants (*J*) are given in Hertz (Hz). All deuterated solvents were used as received without any further purification. [PtCl_2_(DMSO)_2_], **L1**,^15^ complex **1**, **1a**,^22^ and **2**,^23^ were synthesized as reported elsewhere. HR-MS spectra were recorded on a Thermo Q-Exactive instrument equipped with an ESI source. Mass spectra were recorded in the positive mode. Elemental analyses were carried out by Technical Services and Scientific Equipment at Department of Pharmaceutical and Pharmacological Sciences at University of Padua with varioMICRO CHNS, while samples were weighted with a Ultra Analytical Balance Sartorius Cubis MSA 6.6S-000-DM.

#### Synthesis of [Pt­(IV)­(Cl)_2_(**L1**)­(py-PEG)] (**2a**)

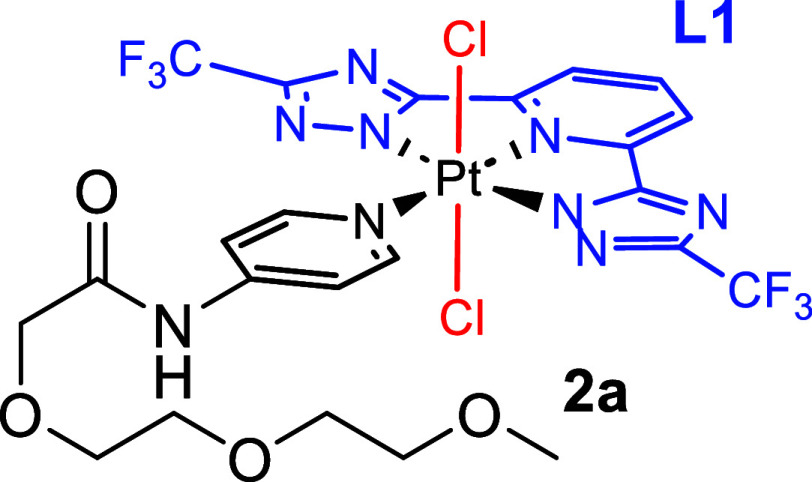



[Pt­(II)­(**L1**)­(**py-PEG**)] (**2**) (100 mg, 0.1256 mmol) and 2 equivof PhICl_2_ (68.8 mg, 0.2512 mmol) are suspended in 5 mL of EtOAc and stirred at *T* = 70 °C for 1 h. The initial bright yellow suspension turned into a homogeneous pale-yellow solution. The final product has been purified by column chromatography on silica gel using a 50/50 DCM/EtOAc mixture as an eluent. Obtained 66.4 mg, 0.0766 mmol as a pale-yellow powder. Yield 61%.

micrOTOF *m*/*z* [M + H]^+^ calcd 868.0711; found 868.0756. Elemental analysis for C_23_H_21_Cl_2_F_6_N_9_O_4_Pt × 1.6H_2_O × ACN × 0.5EtOAc: calcd = C 33.11, H3.19, N 14.30; found = C 33.03, H 3.19, N 14.17. ^1^H NMR (300 MHz, CDCl_3_, 298 K): δ (ppm) = 9.80 (s, 1H; NH amide), 9.35 (dd, ^3^ *J*_HPt_ = 11.5 Hz, ^3^ *J*_HH_ = 7.4 Hz, 2H; *ortho* **py-PEG**), 8.37 (dd, ^3^ *J*_HH_ = 8.6 Hz, ^3^ *J*_HH_ = 7.2 Hz, 1H; *para* py**L1**), 8.26–8.17 (m, 4H; *para* **py-PEG** and *meta* py**L1**), 4.22 (s, 2H; CH_2_), 3.86–3.71 (m, 6H; CH_2_), 3.63 (m, 2H; CH_2_) 3.49 (s, 3H; CH_3_). ^13^C­{^1^H} NMR (75 MHz, CDCl_3_, 298 K): δ = 170.5 (s, *C* amide), 160.6 (s; *ipso* triazole), 153.5 (q, ^2^ *J*_CF_ = 39.3 Hz; *ipso* py**L1**), 152.5 (s; *ortho* **py-PEG**), 149.4 (s, satellites *J*_CPt_ = 2.8 Hz; *ipso* py**L1**), 146.2 (s; *para* py**L1**), 145.4 (s; *para* **py-PEG**), 127.4 (q, ^1^ *J*_CF_ = 427.1 Hz, CF_3_), 121.0 (s, satellites ^3^ *J*_CPt_ = 8.9 Hz; *meta* py**L1**), 116.5 (s, satellites ^3^ *J*_CPt_ = 12.3 Hz; *meta* **py-PEG**), 71.5 (s, CH_2_), 71.4 (s, CH_2_), 71.1 (s, CH_2_), 69.9 (s, CH_2_), 58.9 (s, CH_3_).

#### Synthesis of [Pt­(II)­(**L1**)­(**py-C8**)] (**3**)

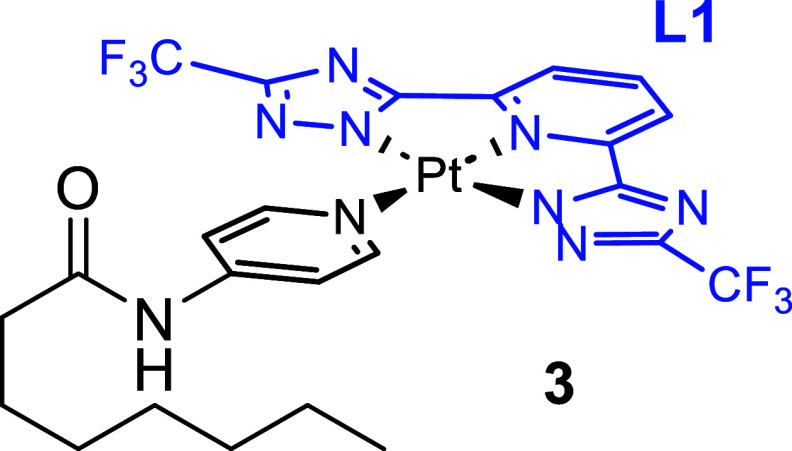



[PtCl_2_(DMSO)_2_] (100.0 mg, 0.2368 mmol) and 1.0 equivof **L**-**2H** (82.7 mg, 0.2368 mmol) are suspended, under an Ar atmosphere, in 10 mL of ACN, followed by the addition of 2.0 eq of DIPEA (61.2 mg, 0.4736 mmol, 82.6 μL). The initial heterogeneous slurry quickly turned into a yellow solution that is left under stirring at RT for 10 min. Afterward, 1.3 equivof **py**-**C8** (72.0 mg, 0.3078 mmol) is added to the solution. The orange solution is stirred at 70 °C overnight without changes in color. The final product was purified via column chromatography on silica gel using a 50/50 mixture or *n*-hexane and ethyl acetate as an eluent. Yield 73% (107.4 mg 0.1729 mmol) as an orange powder.

micrOTOF *m*/*z* [M + H]^+^ calcd 763.1657; found 763.1675. Elemental analysis for C_24_H_23_F_6_N_9_OPt × 2.5H_2_O × 1.5ACN × 0.4 *n*-hexane: calcd = C 39.08, H4.25, N 16.28; found = C 38.99, H 4.23, N 16.31. ^1^H NMR (300 MHz, CDCl_3_, 298 K): δ = 9.42 (d, ^3^ *J*_HH_ = 6.4 Hz, 2H; *ortho* **py-C8**), 8.00 (dd, ^3^ *J*_HH_ = 7.9 Hz, ^3^ *J*_HH_ = 7.9 Hz, 1H; *para* py**L1**), 7.74 (d, ^3^ *J*_HH_ = 7.9 Hz, 2H; *meta* py**L1**), 7.67 (*d*, ^3^ *J*_HH_ = 7.9 Hz, 2H; *meta* **py-C8**), 7.62 (s, 1H; NH amide), 2.52 (t, ^3^ *J*_HH_ = 7.7 Hz, 2H; CH_2_), 1.80 (m, 2H; CH_2_), 1.32 (m, 7H; CH_2_), 0.93 (m, 4H; CH_2_). ^13^C­{^1^H} NMR (75 MHz, CDCl_3_, 298 K): δ = 170.4 (s, *C* amide), 163.8 (s; *ipso* triazole), 154.0 (s, aromatic), 148.2 (s, aromatic), 142.9 (s; aromatic), 118.3 (s; aromatic), 115.2­(s, aromatic), 38.1 (s, CH_2_), 31.8 (s, CH_2_), 29.9 (s, CH_2_), 29.2 (s, CH_2_), 29.1 (s, CH_2_), 22.7 (s, CH_2_), 25.2 (s, CH_2_), 14.2 (s, CH_3_).

#### Synthesis of [Pt­(IV)­(Cl)_2_(**L1**)­(py-C8)] (**3a**)

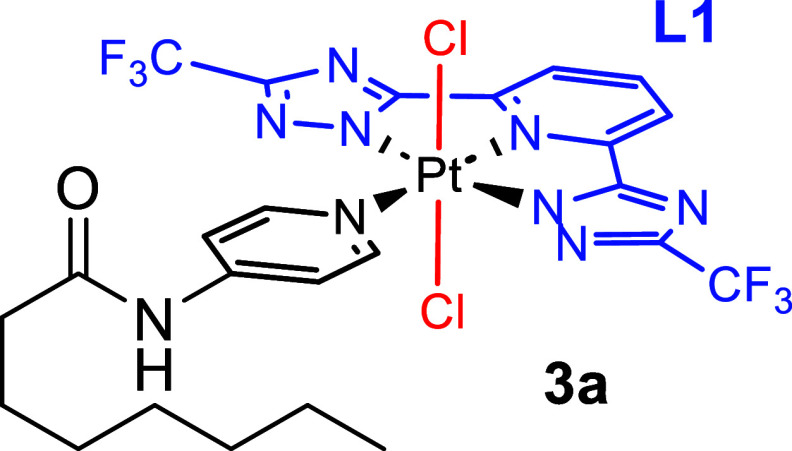



Compound **3a** is synthesized with the same methodology as **2a**, using a 70/30 mixture of an *n*-hexane/EtOAc mixture as the eluent for the chromatography on silica gel. Obtained 77.6 mg, 0.0917 mmol, as a pale-yellow powder. Yield 73%.

micrOTOF *m*/*z* [M + H]^+^ calcd 834.1021; found 834.1036. Elemental analysis for C_24_H_23_Cl_2_F_6_N_9_OPt × 3.5H_2_O × 0.1ACN × 0.5EtOAc: calcd = C 33.31, H3.66, N 13.49; found = C 33.10, H 3.66, N 13.33. ^1^H NMR (300 MHz, CDCl_3_, 298 K): δ (ppm) = 9.30 (dd, ^3^ *J*_HPt_ = 11.3 Hz, ^3^ *J*_HH_ = 7.0 Hz, 2H; *ortho* **py-C8**), 8.41 (dd, ^3^ *J*_HH_ = 8.4 Hz, ^3^ *J*_HH_ = 7.4 Hz, 1H; *para* py**L1**), 8.25 (d, ^3^ *J*_HH_ = 7.8 Hz, 2H; *meta* py**L1**), 7.96 (m, 2H; *meta* **py-C8**), 7.91 (s, 1H; NH amide), 2.53 (t, ^3^ *J*_HH_ = 7.5 Hz, 2H; CH_2_), 1.80 (m, 2H; CH_2_), 1.33 (m, 10H; CH_2_), 0.92 (m, 3H; CH_3_). ^13^C­{^1^H} NMR (75 MHz, CDCl_3_, 298 K): δ = 172.5 (s, *C* amide), 160.8 (s; *ipso* triazole), 153.9 (q, ^2^ *J*_CF_ = 39.6 Hz; *ipso* py**L1**), 152.9 (s; *ortho* **py-PEG**), 149.7 (s, satellites *J*_CPt_ = 2.8 Hz; *ipso* py**L1**), 146.4 (s; *para* py**L1**), 145.6 (s; *para* **py-C8**), 121.3 (s, satellites ^3^ *J*_CPt_ = 8.9 Hz; *meta* py**L1**), 119.4 (q, ^1^ *J*_CF_ = 270.6 Hz, CF_3_), 115.8 (s, satellites ^3^ *J*_CPt_ = 12.3 Hz; *meta* **py-C8**), 38.1 (s, CH_2_), 31.8 (s, CH_2_), 29.2 (s, CH_2_), 29.1 (s, CH_2_), 25.2 (s, CH_2_), 22.7 (s, CH_2_), 14.2 (s, CH_3_).

### General Procedure for Chemiluminescence Experiments

A solution 0.02 M of **1a**-**3a** is made in ACN, while the reducing agent mixture is made with NaBH_4_, sodium ascorbate, and NADH at pH 13 (NaOH) in H_2_O. Inside a light shielded metal box, a 2 mL vial containing 200 μL of the metal complex solution is placed close to the revealer of a PMT, or connected to a CCD camera via optical fiber, and the solution of reducing agent (500 μL) is flesh injected via cannula. The reduction reaction takes place in an instant with development of light which is registered with the desired instrument connected to the reactor.

The experiment at different pH occur similar to what just described, with the only difference that the basic solution in which the reducing agents are dilute is at pH 14, 12, 11, and 9 and with no additional base.

The influence of ionic strength on the CL response was investigated using identical concentrations of luminophore and sodium ascorbate as in the previously reported experiments while varying the pH of the reducing agent mixture (13, 12, and no added base). In all experiments, the total salt concentration was fixed at 0.1 M by dissolving NaCl in place of NaOH when the tested pH differed from 13.

The same experiment (same quantity and concentration of reagents, pH 13) is done by connecting the reactor to a CCD camera via an optical fiber to acquire the emission spectrum of the chemiluminescent reaction.

Comparison of the Pt­(II) aggregation-based CL signal with the reference system [Ru­(II)­bpy_3_]^2+^/[Ru­(III)­bpy_3_]^3+^ was carried out by preparing a bulk solution of the Ru­(III) species in strongly acidic medium and reacting it with sodium ascorbate under different conditions. Specifically, 0.04 mol [Ru­(II)­bpy_3_]­Cl_2_ were dissolved in 2 mL of 9 M H_2_SO_4_ (final complex concentration 0.02 M) and treated with 10 equiv of PbO_2_. The initially orange heterogeneous mixture turned green after 5 min of stirring at RT. The mixture was allowed to react for a total of 30 min and then decanted, and the supernatant was filtered through a Celite pad to remove insoluble material. The resulting [Ru­(III)­bpy_3_]^3+^ solution was used without further purification. For the chemiluminescence test, 100 μL of the 0.02 M solution of [Ru­(III)­bpy_3_]^3+^ in 9 M H_2_SO_4_ was placed in a 2 mL vial and positioned directly on top of the PMT detector. The reducing agent mixture, consisting of 500 μL of 0.04 M sodium ascorbate in aqueous NaOH (pH 13), was flesh injected into the vial via a cannula.

### Stability Study

A sample of aggregates **A3** obtained by the CL reaction between **3a** and sodium ascorbate at pH 13 has been transferred to a quartz Petri dish and monitored via emission analysis (integrating sphere, λ_exc_ = 350 nm) throughout 7 h time span. No modification of the emission profile has been observed during this time period; thus, the compounds are considered to be stable under CL conditions.

### Cyclic Voltammetry

Pt (II) species **1**–**3** are dissolved in ACN at 10^–3^ M concentration, using TBAPF_6_ (0.1 M) as the supporting electrolyte and Ag wire as the reference electrode. For sake of comparison with literature potential described in H_2_O against the Ag/AgCl reference electrode, the 0 V potential in the plotting has been corrected by measuring the potential of the standard Fc^+^/Fc couple (−0.47 V in this setup) and adjusting it by its known value vs Ag/AgCl (0.45 V). The applied potential ranges from 0 to 2 V, from 2 to −2 V, and back from −2 to 0 V at a scan rate of 0.1 V/s.

### Electrochemiluminescence

A solution of final concentration of **2** (10^–3^ M), TBAPF_6_ (0.1 M, supporting electrolyte), and sodium ascorbate (5 × 10^–3^ M, pH13, NaOH) in DMF/H_2_O 2:1 is analyzed via the CV/PMT method. The applied potential ranges from 0 to 2 V (reference electrode Ag wire) with a scan rate of 0.02 V/s, while the development of light is analyzed with a PMT placed in close proximity to the electrochemical reactor. The same setup and luminophore concentration have been used to analyze the ECL signal from a solution of **2** in the presence of PhICl_2_ (final concentration of 0.04 M, DMF/H_2_O 1:1 v/v) in a reduction–oxidation cycle (from 0 V to −2 V, 0.02 V/s).

### Preparation of Samples

Samples for photophysical measurements in solution are obtained by dissolution of 0.005 mmol complex **1–3**, or **1a**–**3a**, in a 50 mL volumetric flask and then diluted to a final concentration of 10^–4^ M, using ACN as the solvent.

Samples for photophysical measurements of aggregates are obtained by direct deposition of 20 μL of ACN containing the Pt (IV) complex at 0.02 M concentration and adding an equal volume of the desired reducing agent at 0.04 M concentration aq directly on the plate cuvette. Samples for the integrating sphere were obtained by abstraction with a spatula or pipet from preformed aggregates.

Samples for fluorescent microscopy are obtained accordingly with chemiluminescence experiments, and a drop of supernatant containing the aggregates is transferred to a quartz Petri dish and irradiated at 475 nm.

### Photophysical Measurements

Absorption spectra were recorded and baseline corrected with a Varian Cary 100bio UV–vis spectrophotometer in 10 mm quartz cuvettes filled with nondegassed solution of investigated compounds. Emission and excitation spectra were recorded with a photoluminescence spectrometer (Edinburgh Instruments, FLS1000) equipped with a 450 W Xe lamp and an air-cooled single-photon counting photomultiplier (Hamamatsu R13456). The aggregates samples were directly formed onto a quartz plate cuvette, while **1**–**3** and **1a**-**3a** solutions were analyzed in a 10 mm quartz cuvette. Emission and excitation spectra were corrected for the source intensity (lamp and grating) and emission spectral response (detector and grating) by standard correction curves. The absolute photoluminescence quantum yields (PLQY) were measured on a Hamamatsu Absolute PL quantum yield spectrometer C11347 Quantaurus QY integrating sphere under air-equilibrated conditions using an empty quartz Petri dish as a reference. Lifetimes were obtained on a FLS1000 in a quartz plate cuvette under air-equilibrated condition. Decay curves were analyzed with Fluoracle Software using the IRF convolution fitting (for lifetime in the nanosecond range) or the tail-fitting for longer lifetimes.

### DFT Calculation

All calculations were performed with ORCA v 6.0.1.^33^ Molecular geometries were preoptimized without any symmetry constrain in the gas phase using the BP86 functional.^34^ Scalar relativistic effects were modeled using the Zeroth Order Regular Approximation (ZORA)^35^ with the ZORA-Def2-SVP^36^ basis set and the SARC-ZORA-TZVP^37^ for platinum with the RI^38^ approximations and the related auxiliary basis sets (SARC/J).^39,40^ The D3 ^41^ dispersion correction with Becke-Johnson^42^ damping was used. All optimized structures were verified as true minima by the absence (Nimag = 0) of negative eigenvalues in the harmonic vibrational frequency analysis. Time-dependent DFT calculations including spin–orbit effects (SOC TD-DFT) calculations were performed in the gas phase using the PBE0 functional.^43^ Scalar relativistic effects were modeled using ZORA with the ZORA-Def2-TZVP^36^ basis set and the SARC-ZORA-TZVPP^37^ for platinum with the RI-SOMF­(1X)^38,44^ approximations with the related auxiliary basis sets (SARC/J).^39,40^ For the TD-DFT calculations, 25 roots were computed including both singlet and triplet SOC effects (DOSOC = true) and the Tamm-Dancoff approximation (TDA = true) to speed up the calculations. Geometries in the excited state were optimized without any symmetry constrain in the gas phase using the PBE0 functional with scalar relativistic effects were modeled using the ZORA in conjunction with the ZORA-Def2-SVP basis set and the SARC-ZORA-TZVPP for platinum with the RI-SOMF­(1X) approximations and the related auxiliary basis sets (SARC/J) selecting the appropriate root and multiplicity in the SOC TD-DFT) calculations. Then, the energy was obtained as a single-point energy calculation from the optimized geometry in the gas phase using the PBE0 functional with scalar relativistic effects modeled using the ZORA in conjunction with the ZORA-Def2-TZVPP basis set and the SARC-ZORA-TZVPP for platinum with the RI-SOMF­(1X) approximations and the related auxiliary basis sets (SARC/J). Structures were visualized with Chemcraft (https://www.chemcraftprog.com). The xyz coordinates, in conjunction with the Pt–Pt distances and electronic energies, are reported in the Supporting Information.

## Supplementary Material



## Data Availability

Source Data are provided with this manuscript. The Cambridge Crystallographic Data Centre (CCDC) Web site contains the supplementary crystallographic data for this paper under identifiers 2371629 for complex **1a**.^22^ This data can be obtained free of charge at www.ccdc.cam.ac.uk/data_request/cif, or by emailing data_request@ccdc.cam.ac.uk, or by contacting The Cambridge Crystallographic Data Centre, 12 Union Road, Cambridge CB2 1EZ, UK; fax: +44 1223 336033.

## Notes

Numerical values of data shown as graphs, as well as all additional data, are available from the corresponding authors. The authors declare that the data supporting the findings of this study are available within the article and its Supporting Information files. The Supporting Information is available free of charge at https://pubs.acs.org/doi/10.1021/jacs.5c12281.

NMR characterization; ^1^H NMR spectrum (300 MHz); ^13^C­{^1^H} NMR spectrum (75 MHz); ^1^H–^1^H COSY 2D NMR spectrum (300 MHz); ^1^H–^1^H NOESY 2D NMR spectrum (300 MHz); ^1^H–^13^C HMBC 2D NMR spectrum; ^1^H–^13^C HSQC 2D NMR spectrum; ^13^C­{^1^H} NMR spectrum (75 MHz); HRMS of compound **3**, **2a**, **3a**; chemiluminescence profiles of compound **3a** under different conditions; photoluminescence spectrum of aggregates **A2–Asc** and chemiluminescence emission spectrum obtained from exergonic reduction of **2a**; emission spectra of **A3**, **2**; CV of comp. **1**, **2**, **3**; UV–vis spectrum of complex 1–3 dissolved in ACN and complex 1a–3a dissolved in ACN; and absorption spectrum of gel and aggregates **A1**–**A3** in ACN (PDF)

## Abbreviations

ACN: acetonitrile
CL: chemiluminescence
ECL: electrochemiluminescence
DCM: dichloromethane
EtOAc: ethyl acetate
DMF: *N*,*N*-dimethylformamide
DMSO: dimethyl sulfoxide
CCD: charge-coupled device
MeOH: methanol
NADH: nicotinamide adenine dinucleotide
PMT: photomultiplier tube
