# Supplementary material for: Reinventing Chemiluminescence through Redox-Driven Self-Assembly
Source: J Am Chem Soc. 2025 Oct 14;147(43):39482–93. doi: 10.1021/jacs.5c12281 (PMC12576776; doi:10.1021/jacs.5c12281)
Supplement: Supplementary file 1 [file ja5c12281_si_001.pdf]

Supplementary Information

Reinventing Chemiluminescence through Redox-Driven Self-assembly

Authors: Dario Alessi[1], Luca Morgan[1], Elisa Pelorosso[1], Mirco Scaccaglia,[1] Piermaria Pinter,[2] Alessandro Aliprandi[1]*.

Corresponding author: alessandro.aliprandi@unipd.it

The PDF file includes:

Figs. S1 to S37

Table S1

Supplementary Text

NMR characterization

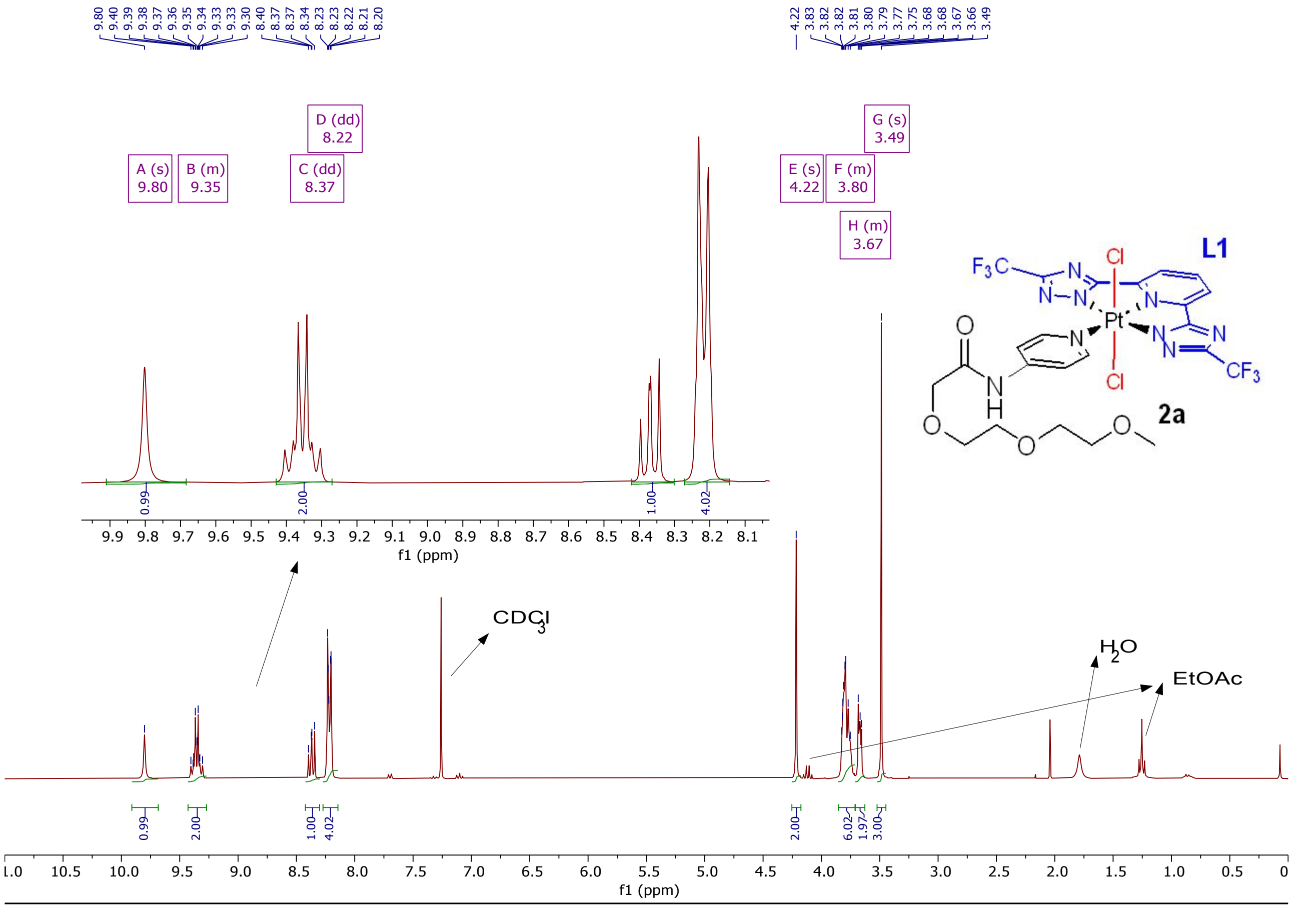

Figure S1: ^{1}H NMR spectrum (300 MHz) of [Pt(IV)(**L1**)(Cl)$_2$(**py-PEG**)] (**2a**) in $CDCl_3$ at 25 °C.

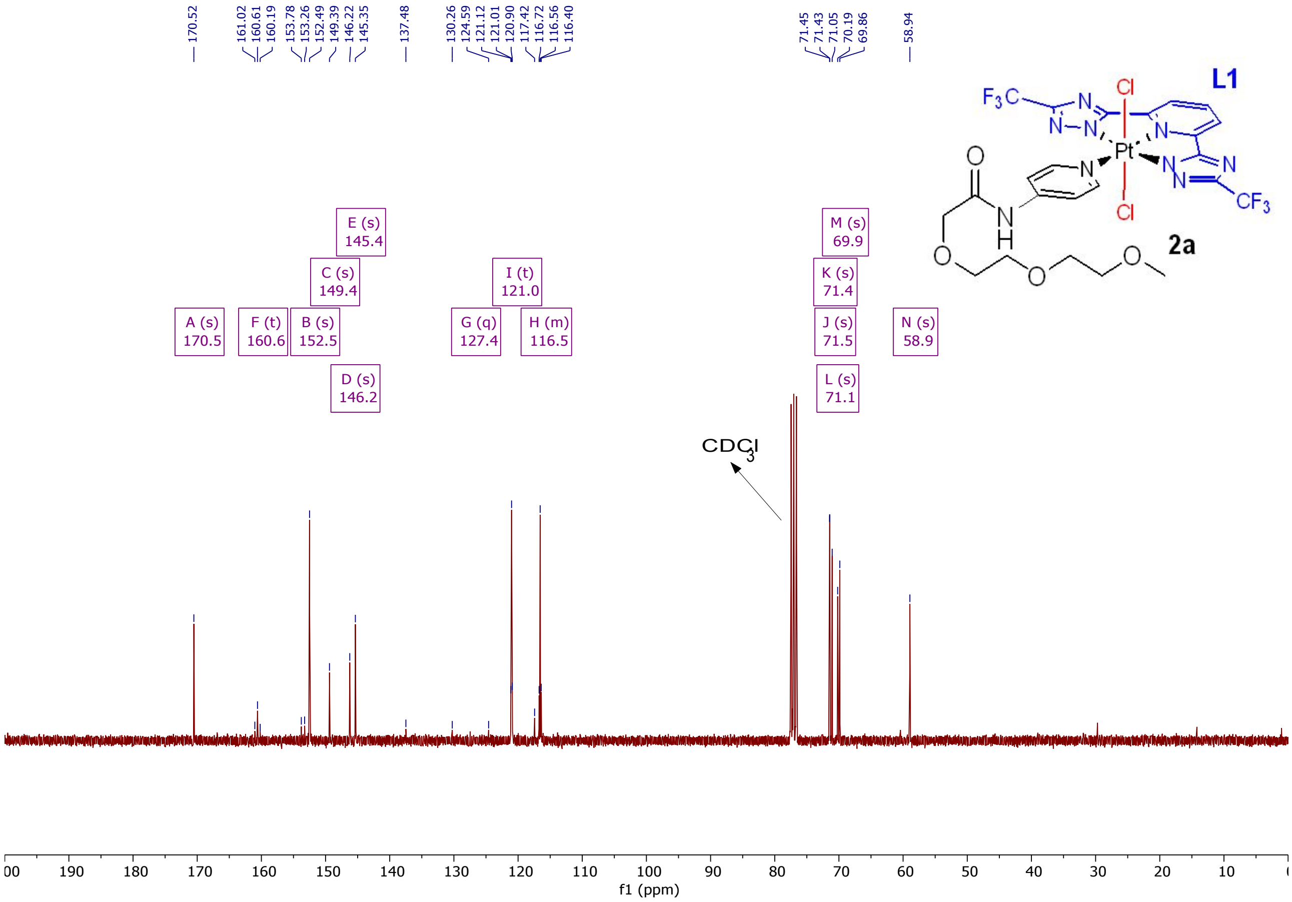

Figure S2: $^{13}C\{^{1}H\}$ NMR spectrum (75 MHz) of [Pt(IV)(**L1**)$(Cl)_2$(**py-PEG**)] (**2a**) in $CDCl_3$ at 25 °C.

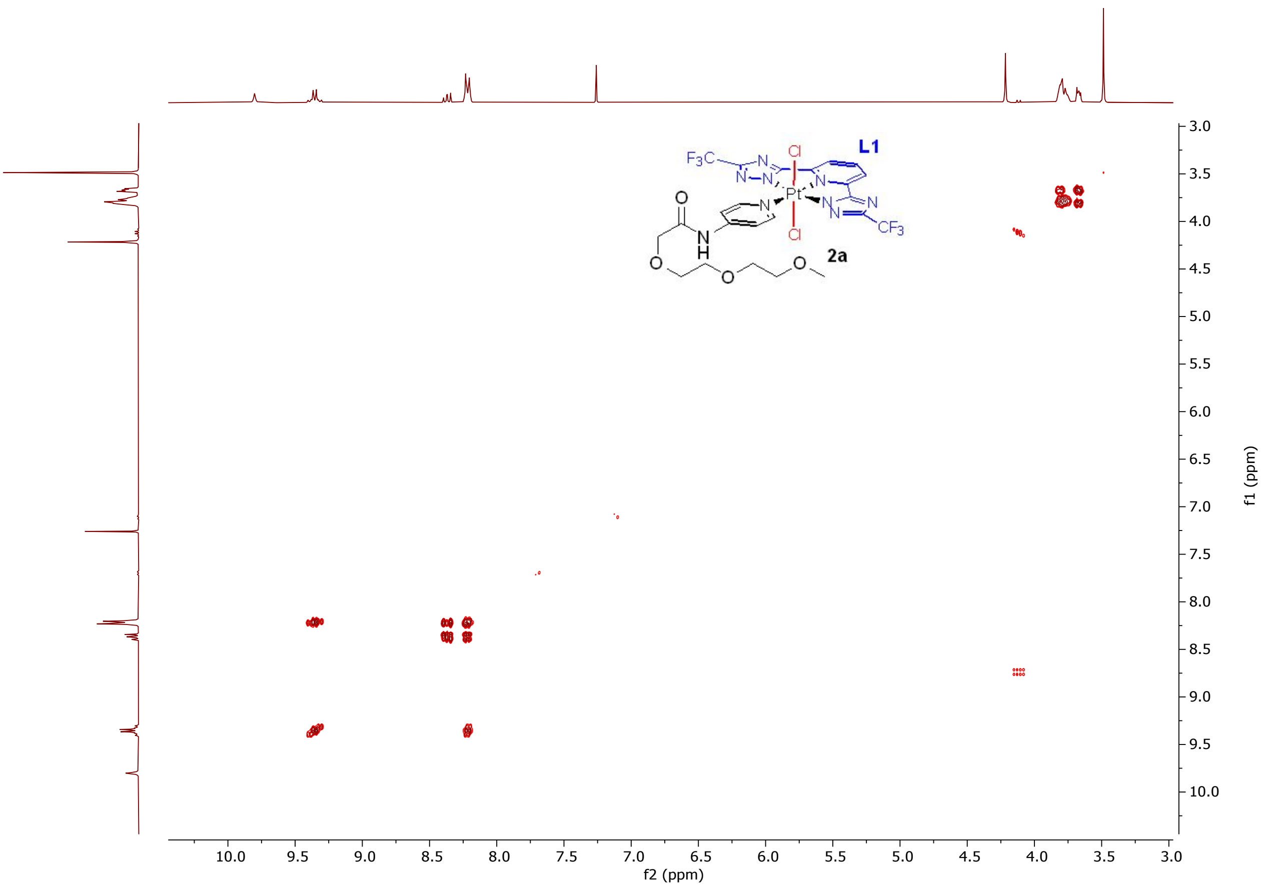

Figure S3: ^{1}H-^{1}H COSY 2D NMR spectrum (300 MHz) of [Pt(IV)(**L1**)(Cl)$_2$(**py-PEG**)] (**2a**) in $CDCl_3$ at 25 °C.

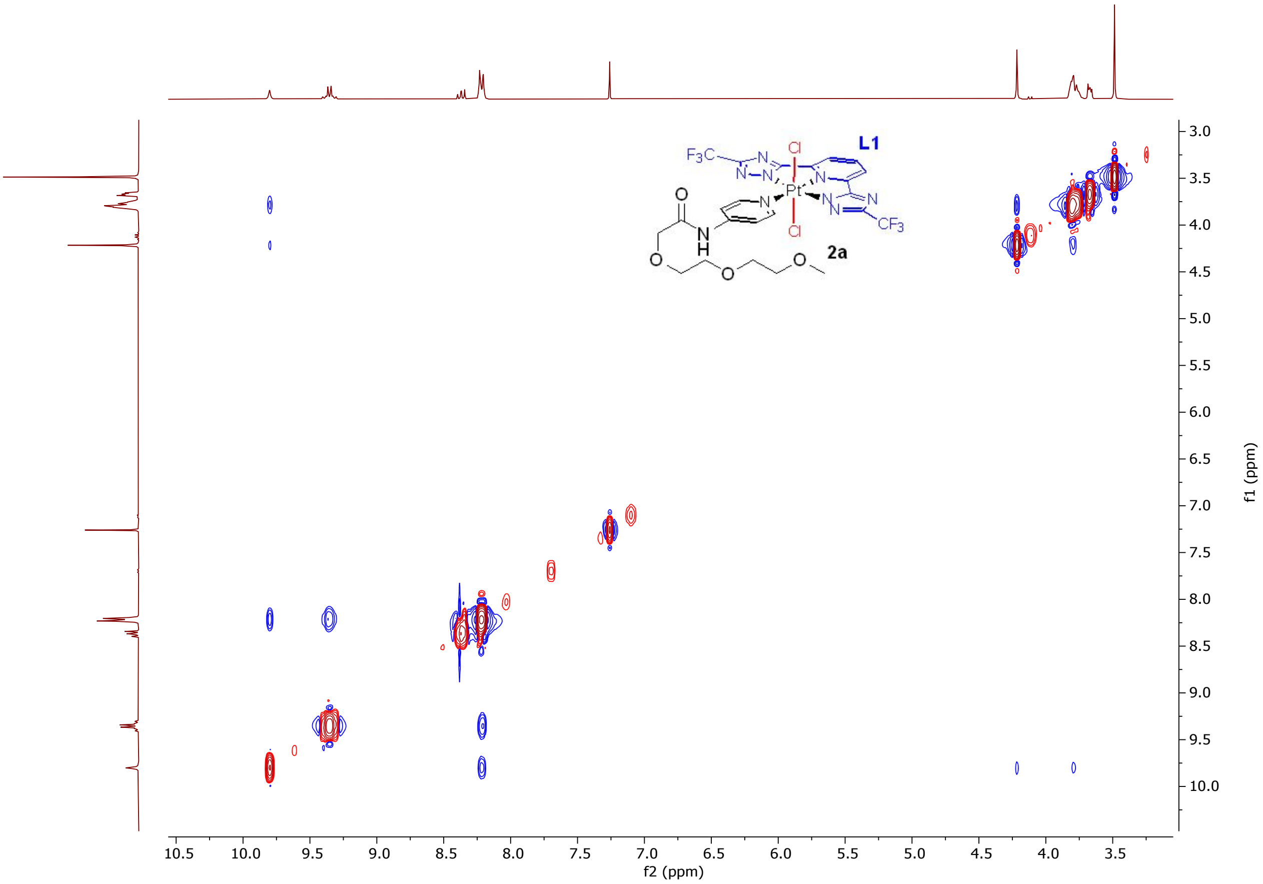

Figure S4: ^{1}H-^{1}H NOESY 2D NMR spectrum (300 MHz) of [Pt(IV)(**L1**)(Cl)$_2$(**py-PEG**)] (**2a**) in $CDCl_3$ at 25 °C.

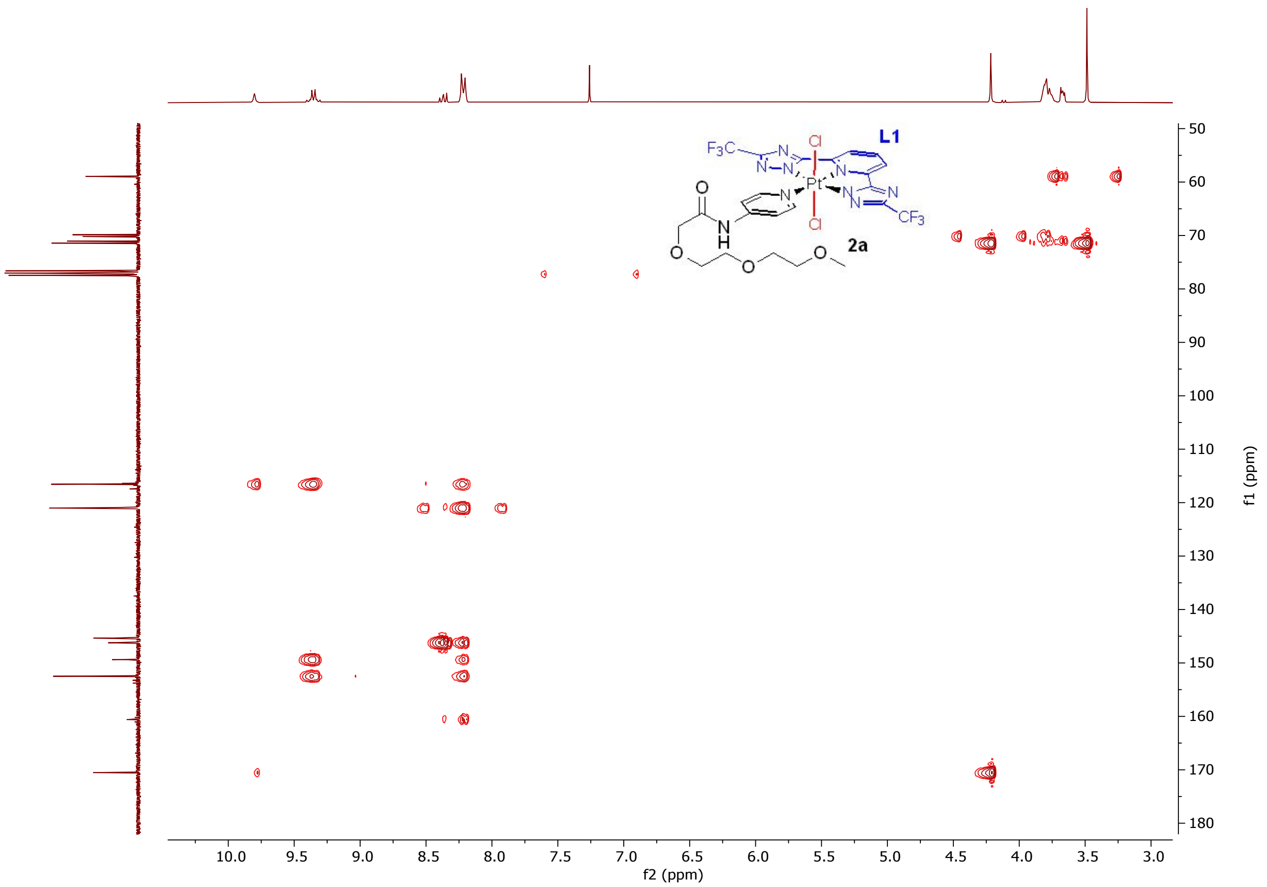

Figure S5: ^{1}H-^{13}C HMBC 2D NMR spectrum of [Pt(IV)(**L1**)(Cl)$_2$(**py-PEG**)] (**2a**) in $CDCl_3$ at 25 °C.

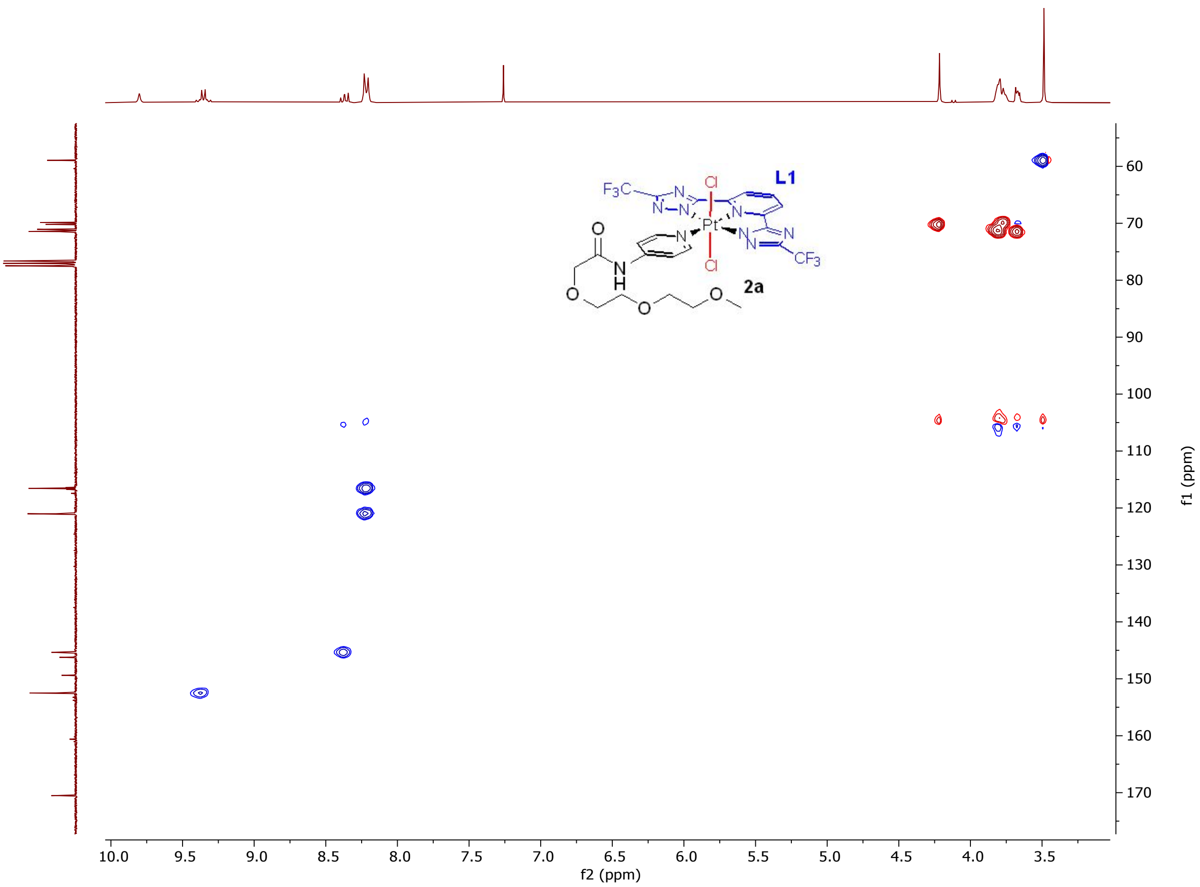

Figure S6: ^{1}H-^{13}C HSQC 2D NMR spectrum of [Pt(IV)(**L1**)(Cl)$_2$(**py-PEG**)] (**2a**) in $CDCl_3$ at 25 °C.

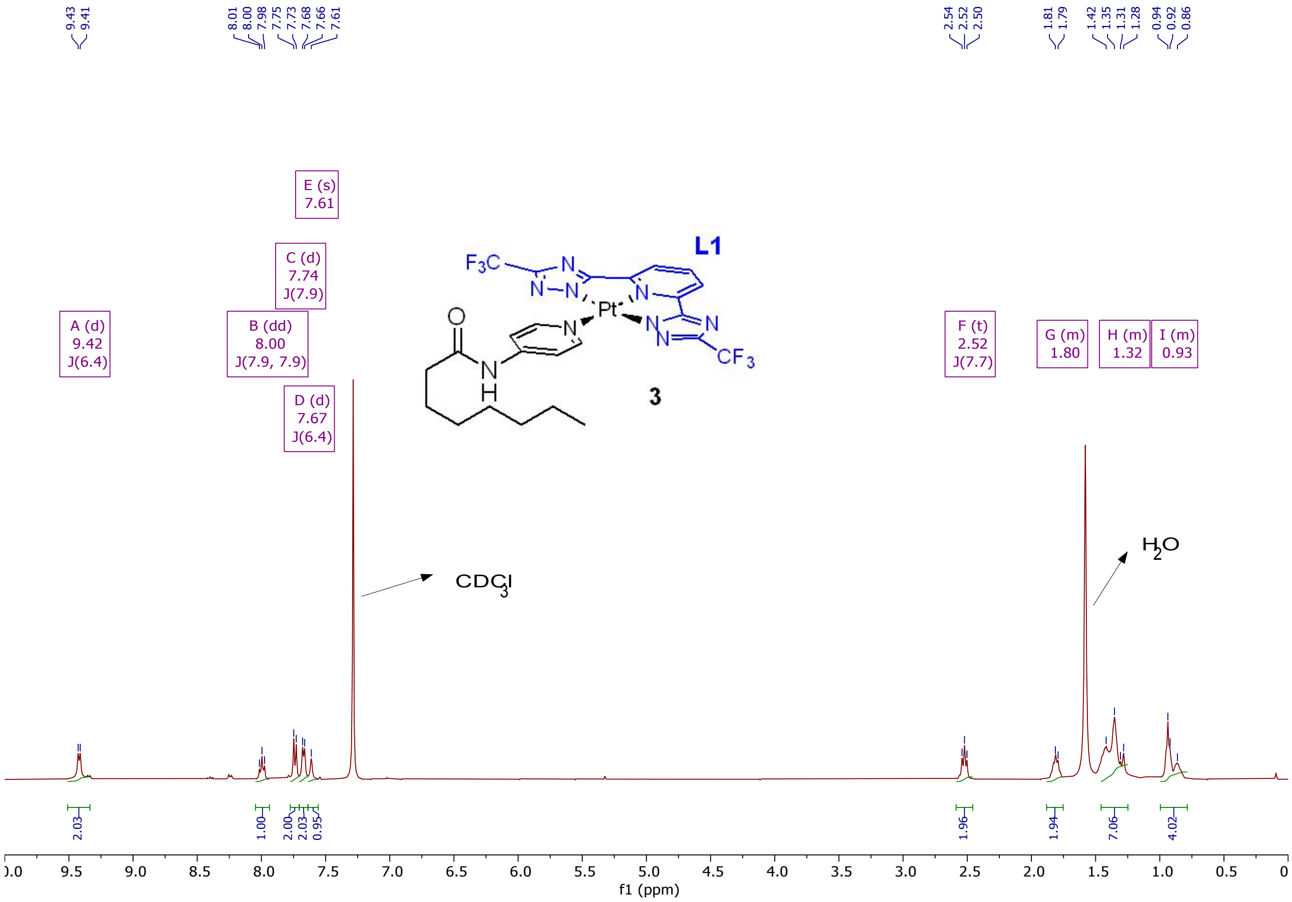

Figure S7: ^{1}H NMR spectrum (75 MHz) of [Pt(II)(**L1**)(**py-C8**)] (**3**) in $CDCl_3$ at 25 °C.

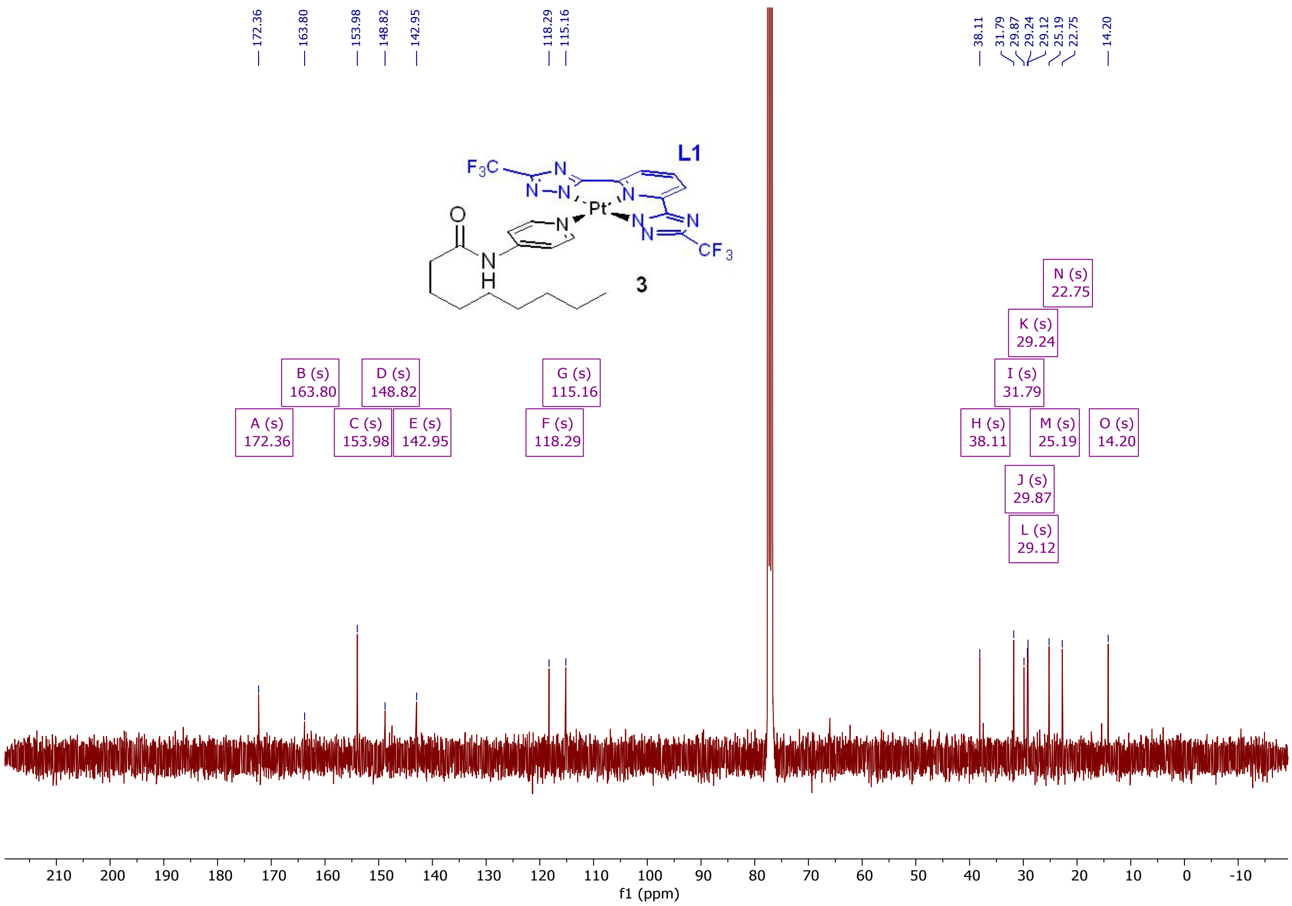

Figure S8: $^{13}C\{^{1}H\}$ NMR spectrum (75 MHz) of [Pt(II)(**L1**)(**py-C8**)] (**3**) in $CDCl_3$ at 25 °C.

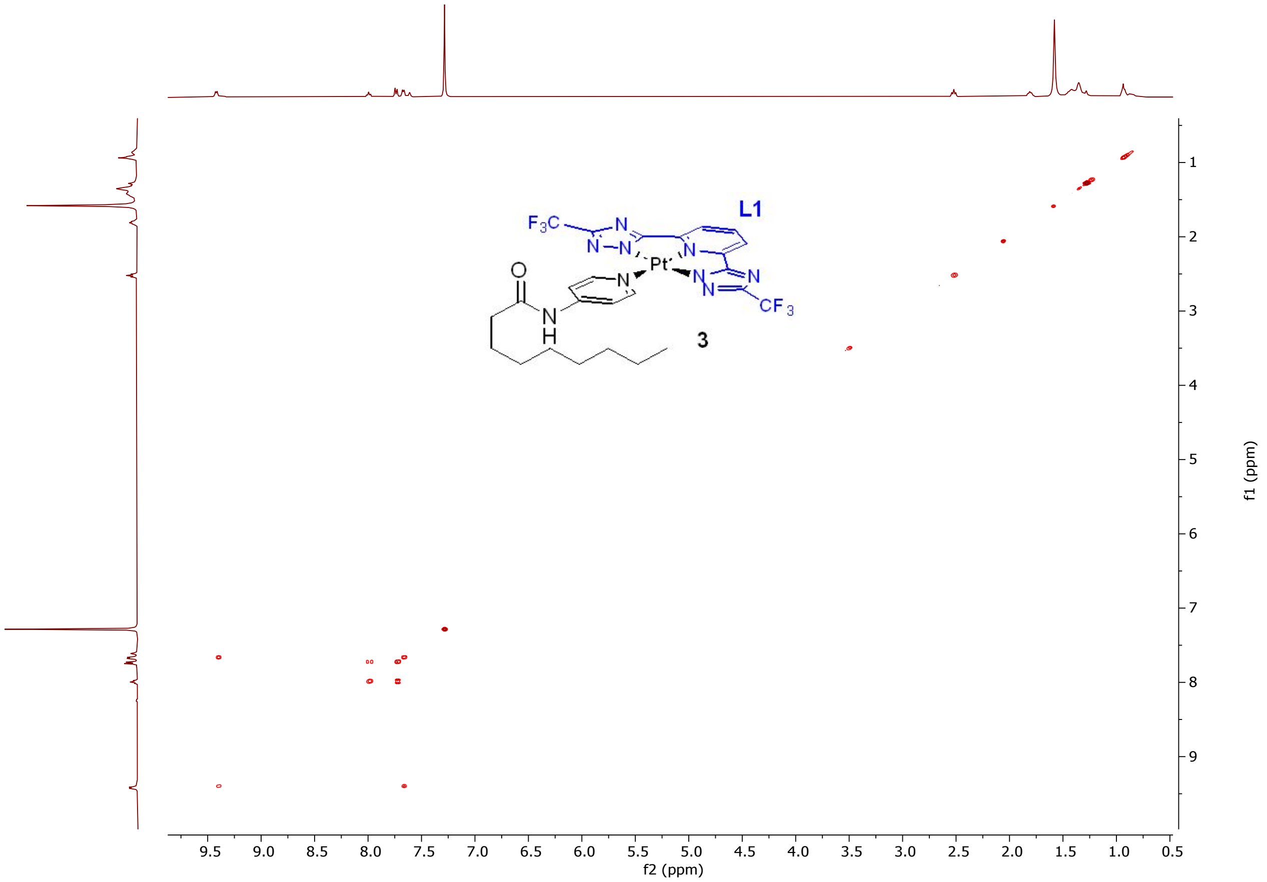

Figure S9: ^{1}H-^{1}H COSY 2D NMR spectrum (300 MHz) of [Pt(II)(**L1**)(**py-C8**)] (**3**) in $CDCl_3$ at 25 °C.

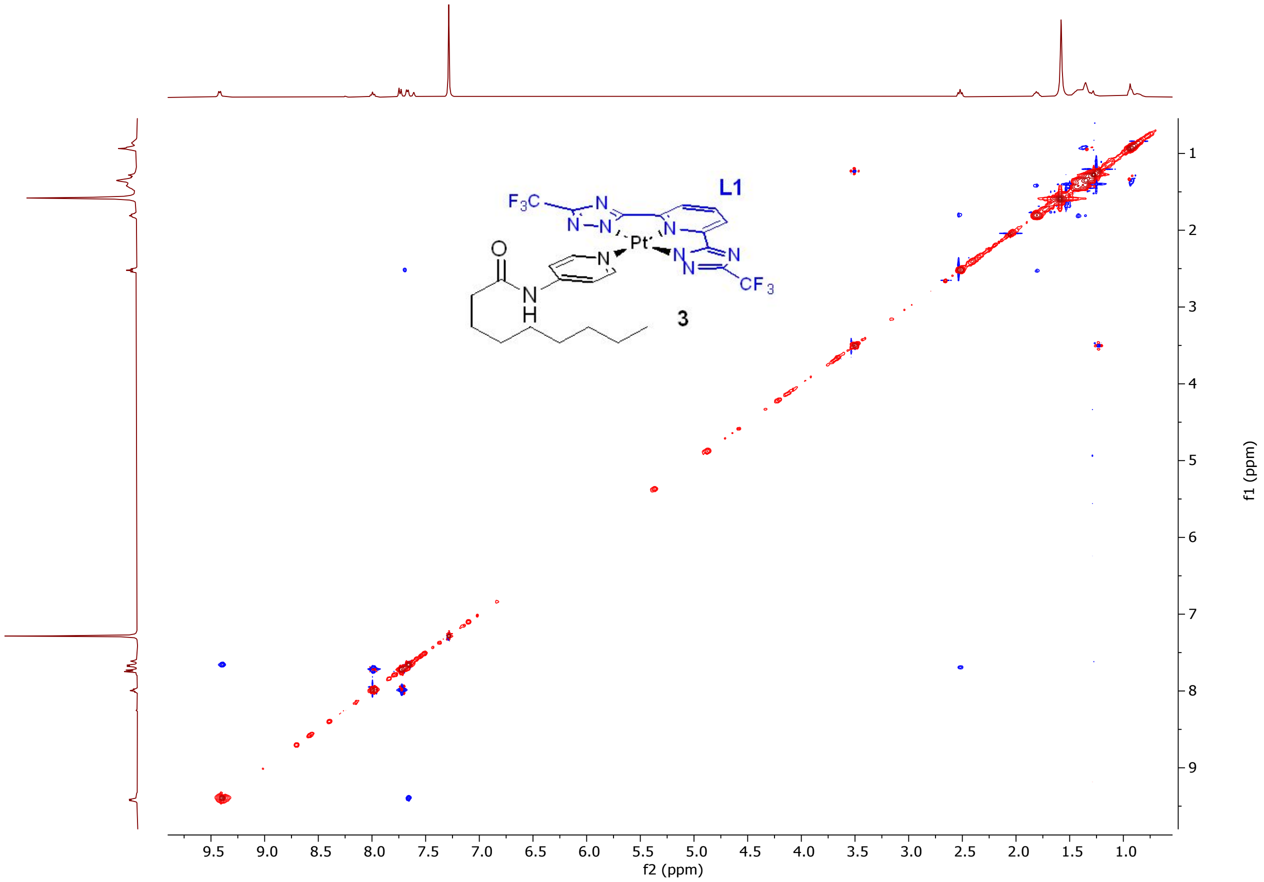

Figure S10: ^{1}H-^{1}H NOESY 2D NMR spectrum (300 MHz) of [Pt(II)(**L1**)(**py-C8**)] (**3**) in $CDCl_3$ at 25 °C.

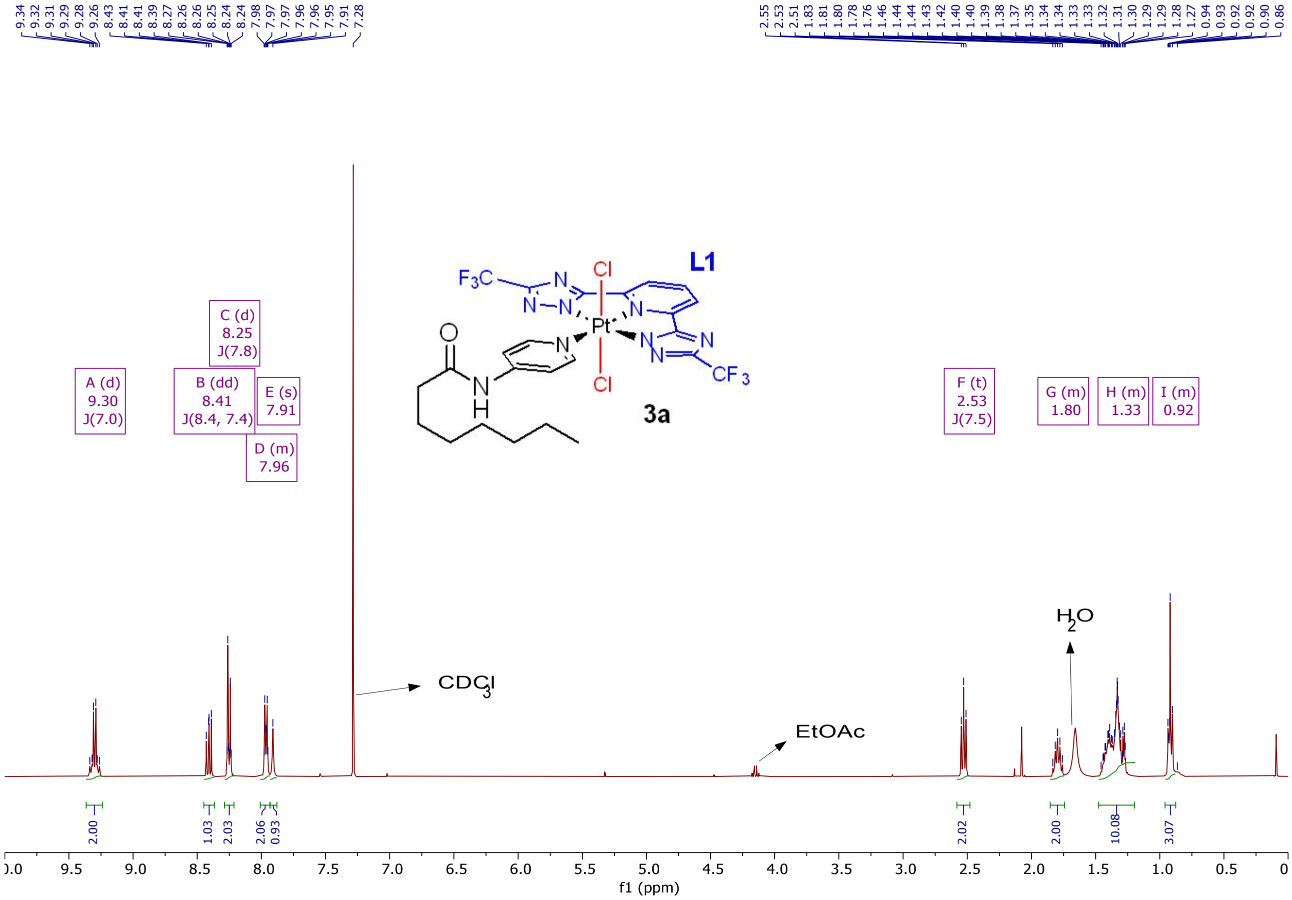

Figure S11: ^{1}H NMR spectrum (300 MHz) of [Pt(IV)(**L1**)(Cl)$_2$(**py-C8**)] (**3a**) in $CDCl_3$ at 25 °C.

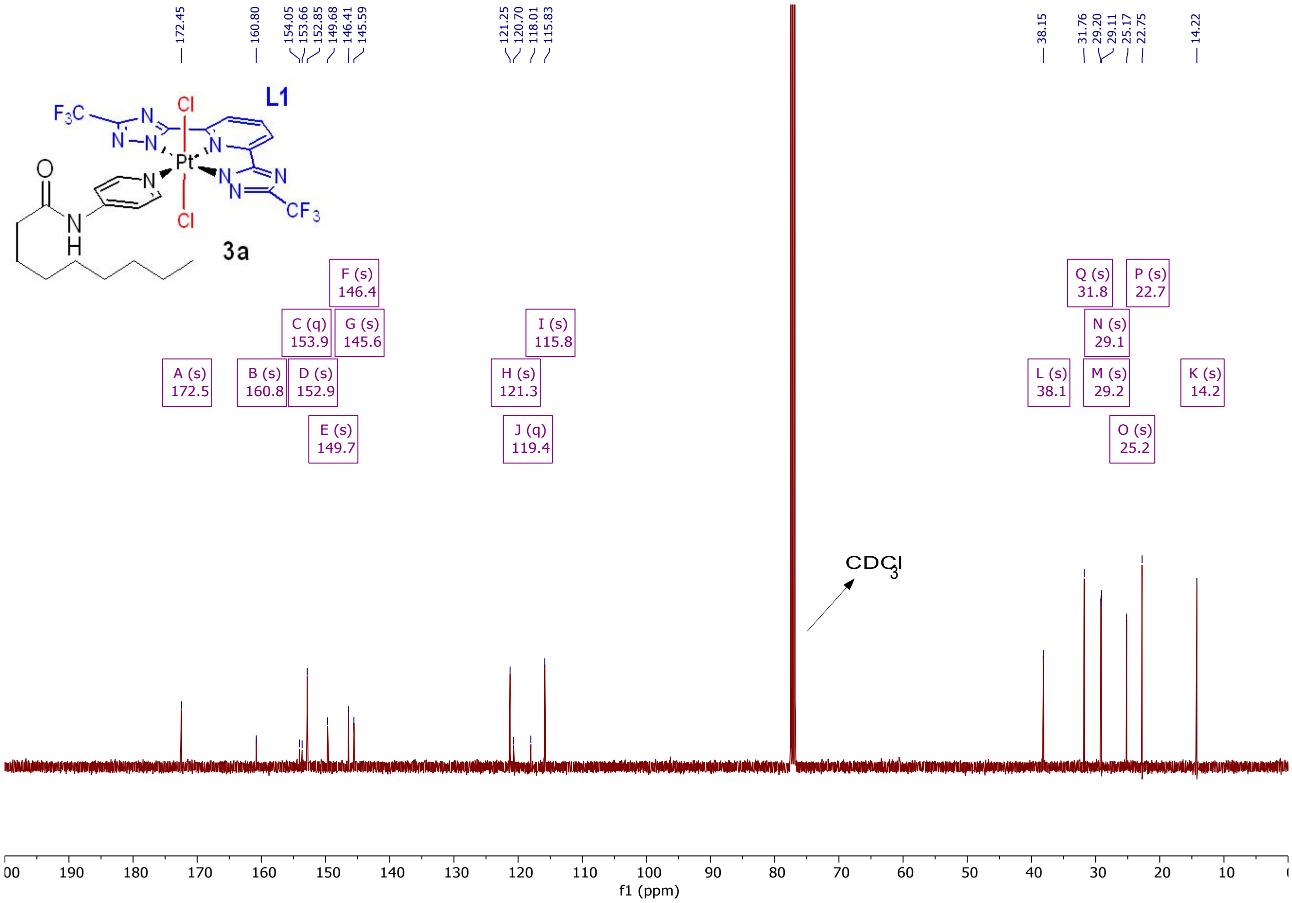

Figure S12: $^{13}C\{^{1}H\}$ NMR spectrum (75 MHz) of [Pt(IV)(**L1**)(Cl)$_2$(**py-C8**)] (**3a**) in $CDCl_3$ at 25 °C.

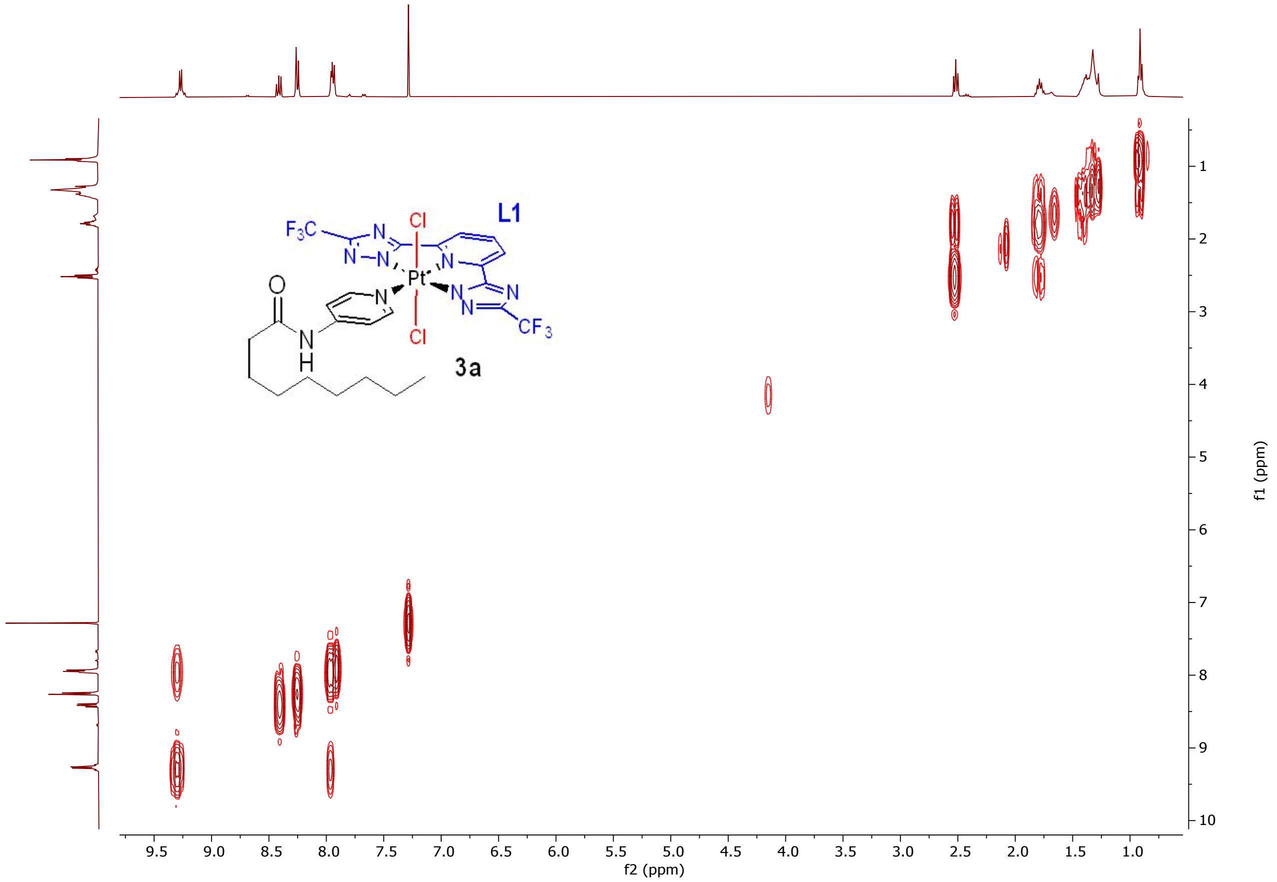

Figure S13: ^1H-^1H COSY 2D NMR spectrum (300 MHz) of [Pt(IV)(**L1**)(Cl)$_2$(**py-C8**)] (**3a**) in $CDCl_3$ at 25 °C.

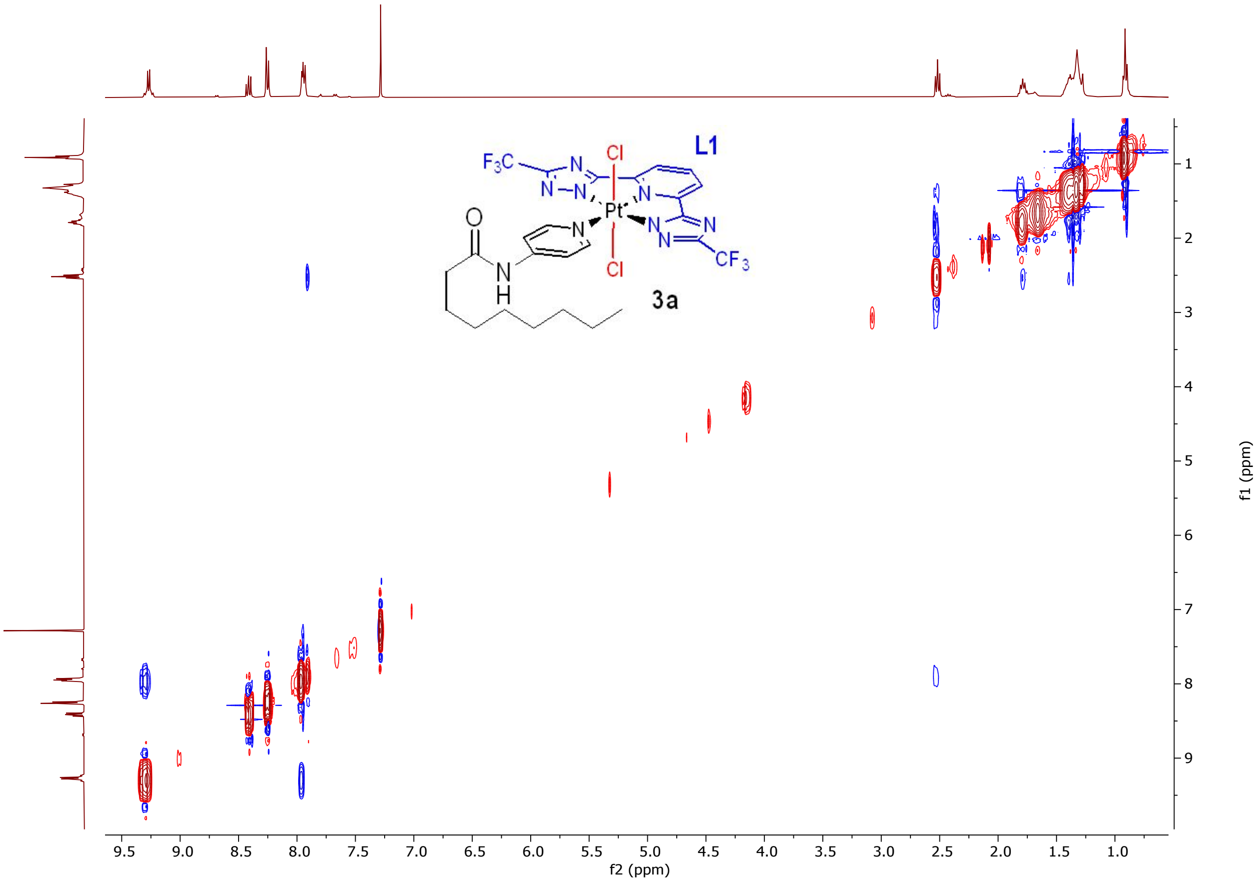

Figure S14: ^{1}H-^{1}H NOESY 2D NMR spectrum (300 MHz) of [Pt(IV)(**L1**)(Cl)$_2$(**py-C8**)] (**3a**) in $CDCl_3$ at 25 °C.

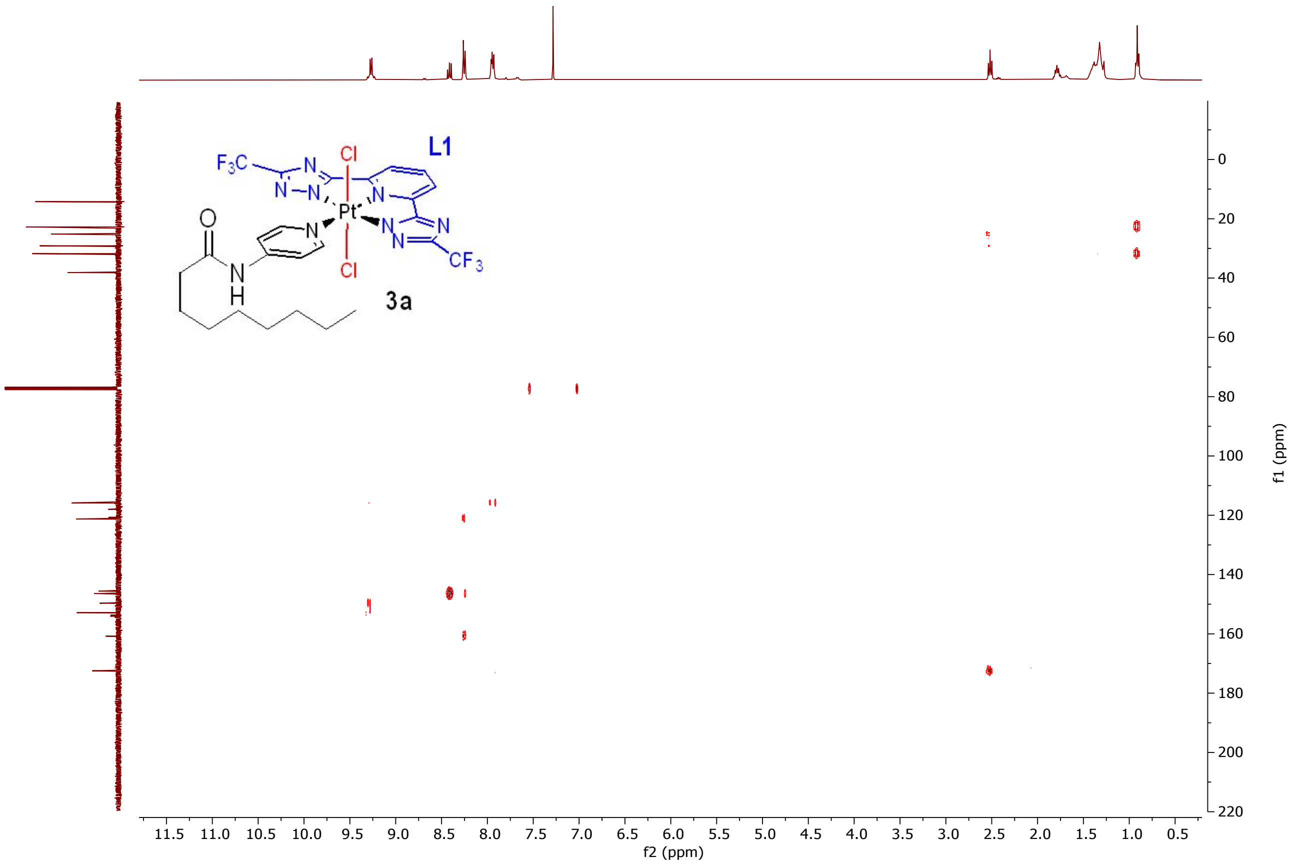

Figure S15: ^{1}H-^{13}C HMBC 2D NMR spectrum of [Pt(IV)(**L1**)(Cl)$_2$(**py-C8**)] (**3a**) in $CDCl_3$ at 25 °C.

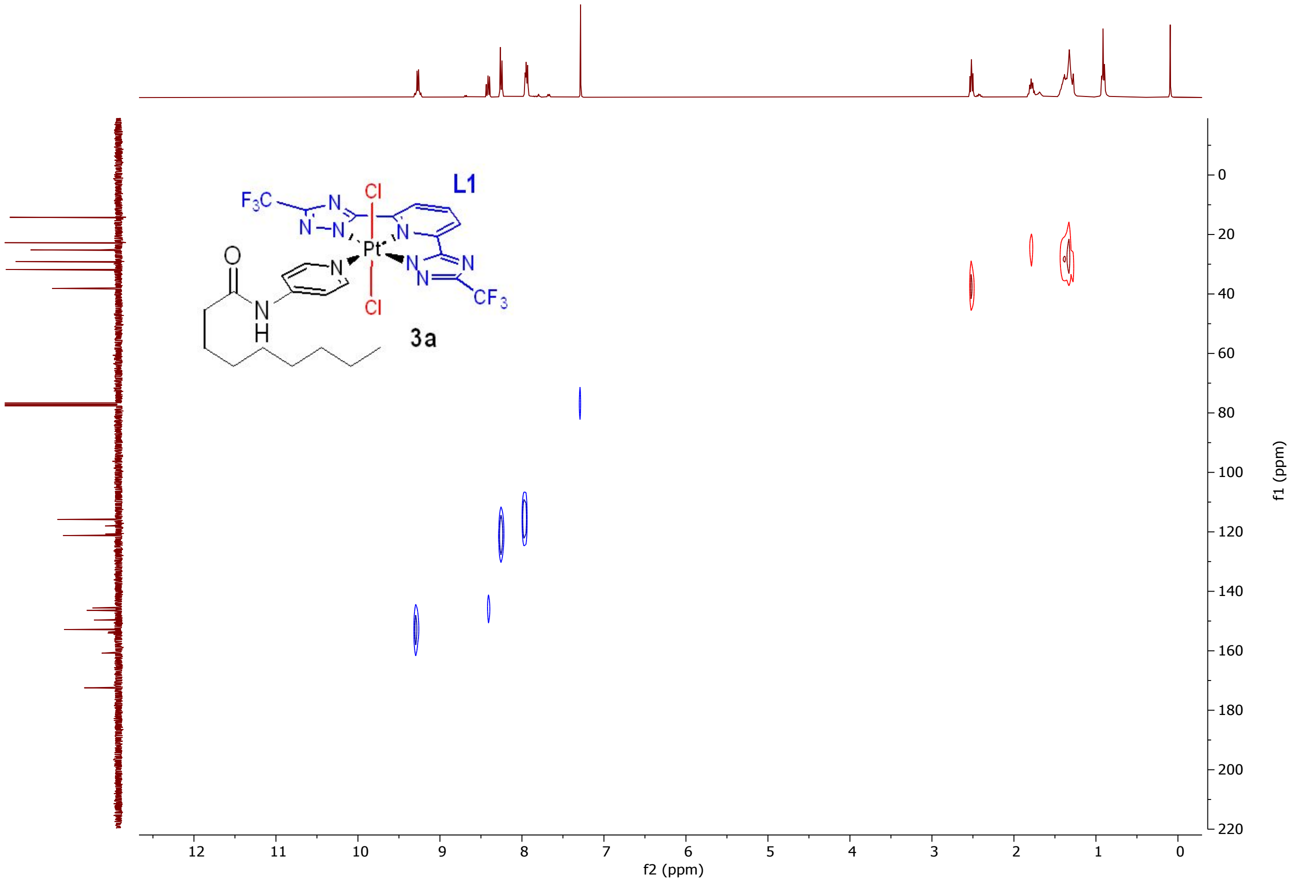

Figure S16: ^{1}H-^{13}C HSQC 2D NMR spectrum of [Pt(IV)(**L1**)(Cl)$_2$(**py-C8**)] (**3a**) in $CDCl_3$ at 25 °C.

Mass Spectra of compound **3**

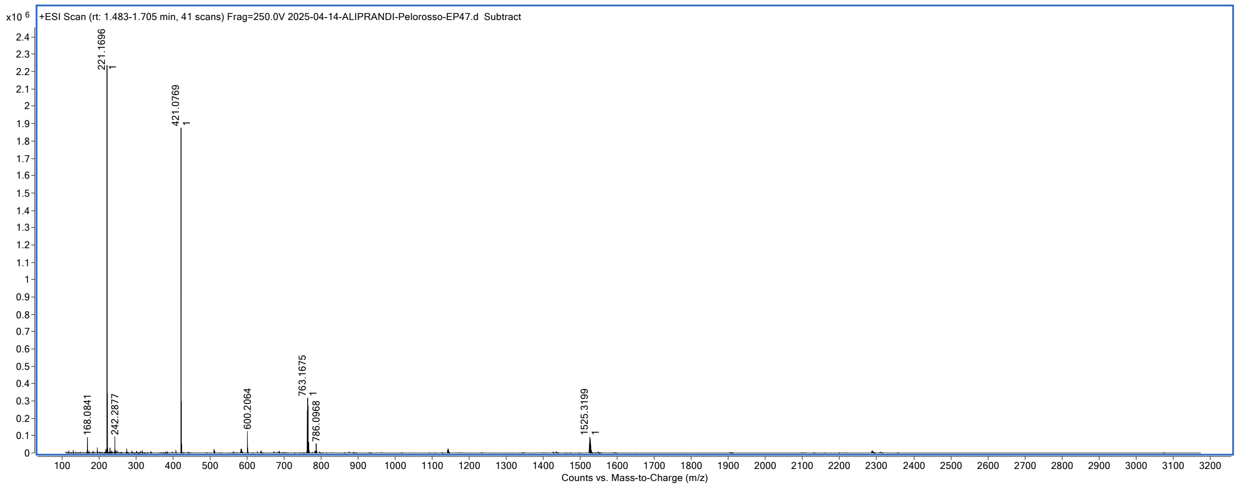

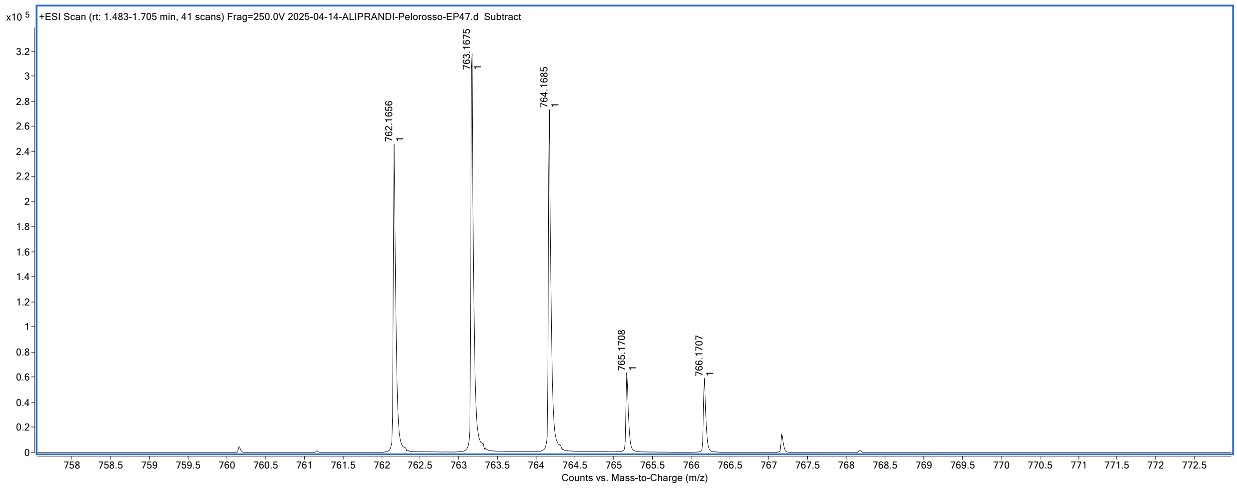

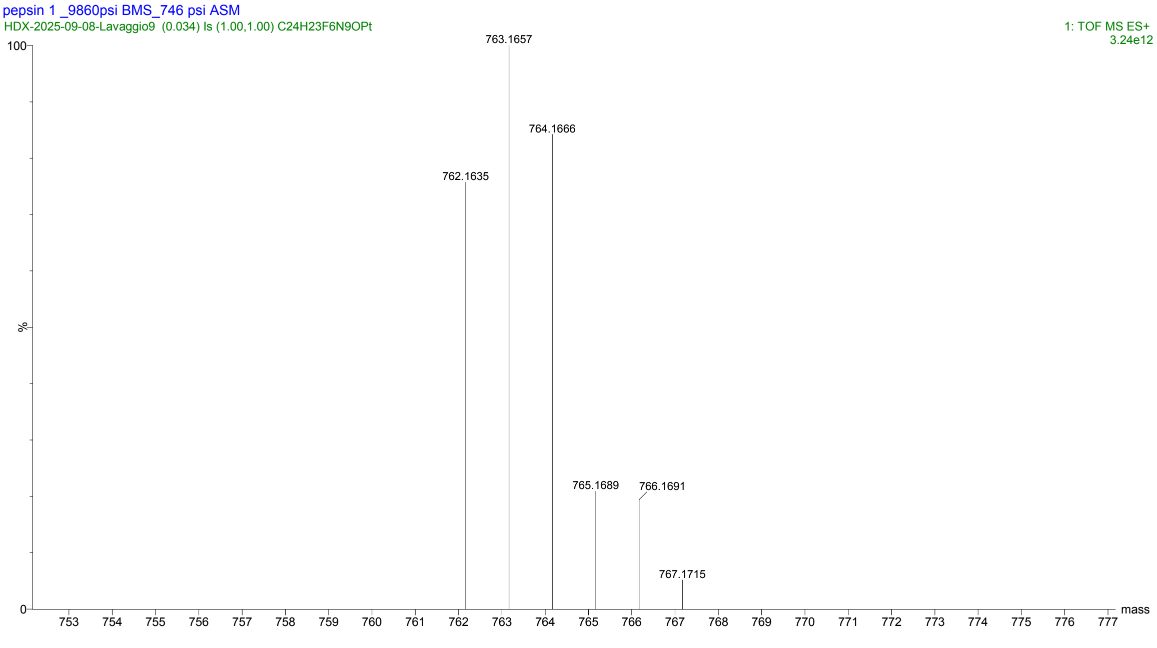

Figure S17: HRMS of compound **3** (positive mode); top: experimental Total-Ion Counting (TIC); middle: experimental isotopic distribution of molecular ion; bottom: calculated isotopic distribution of molecular ion.

Mass Spectra of compound **2a**

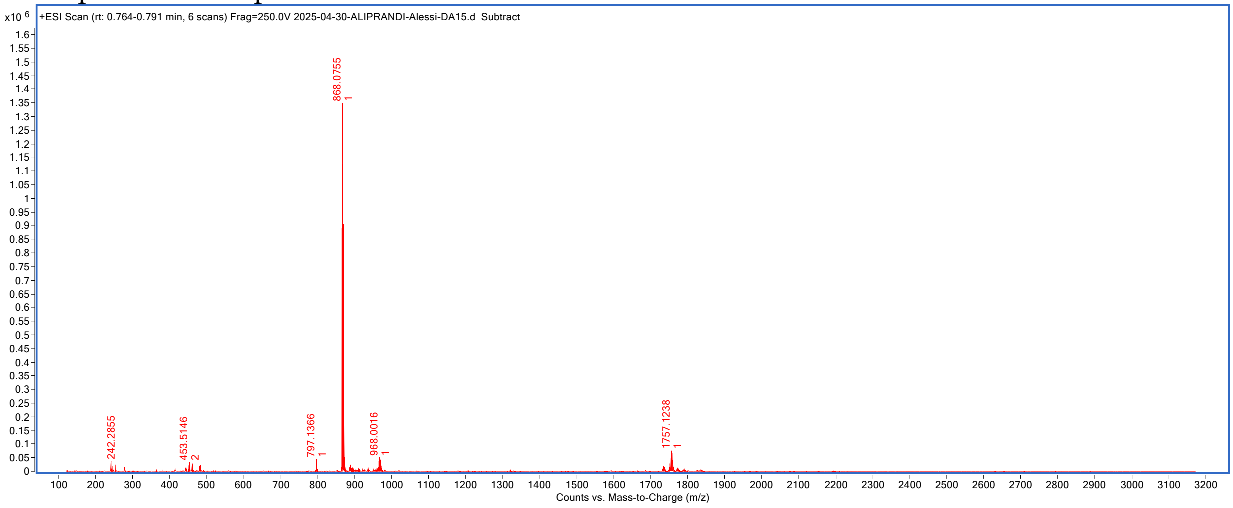

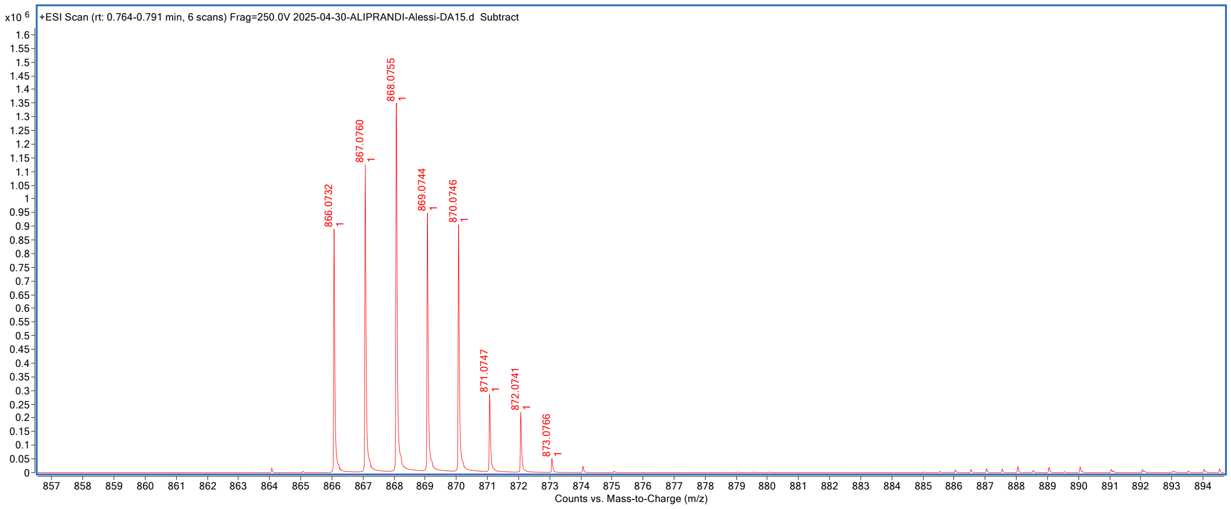

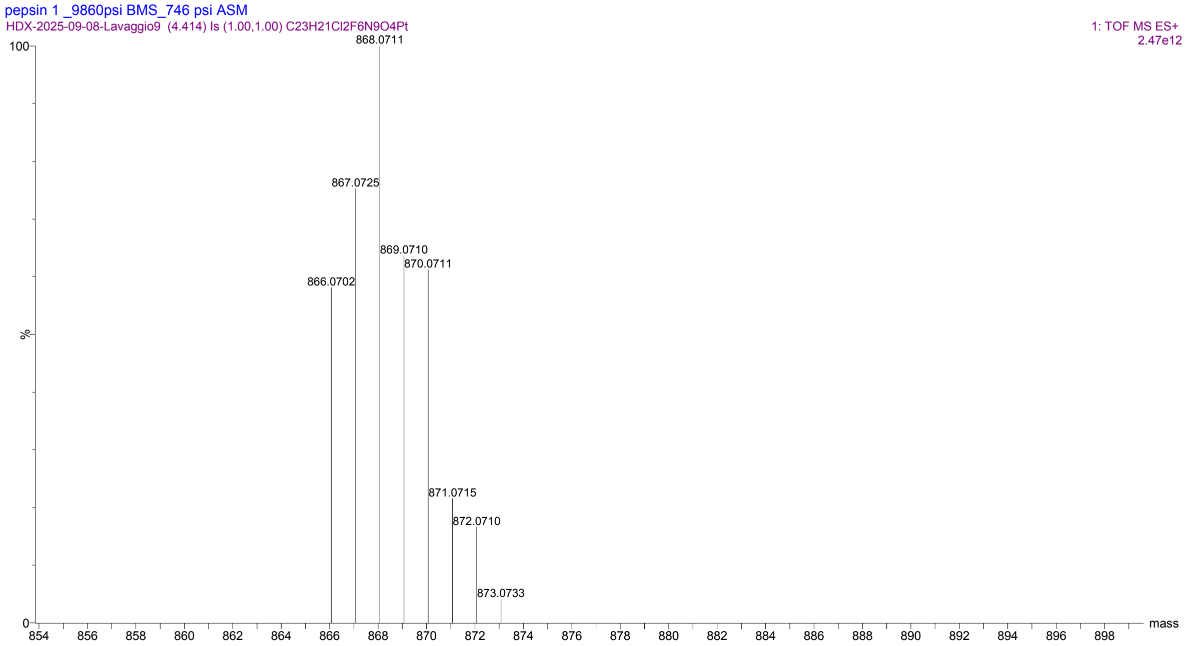

Figure S18: HRMS of compound **2a** (positive mode); top: experimental Total-Ion Counting (TIC); middle: experimental isotopic distribution of molecular ion; bottom: calculated isotopic distribution of molecular ion.

Mass Spectra of compound **3a**

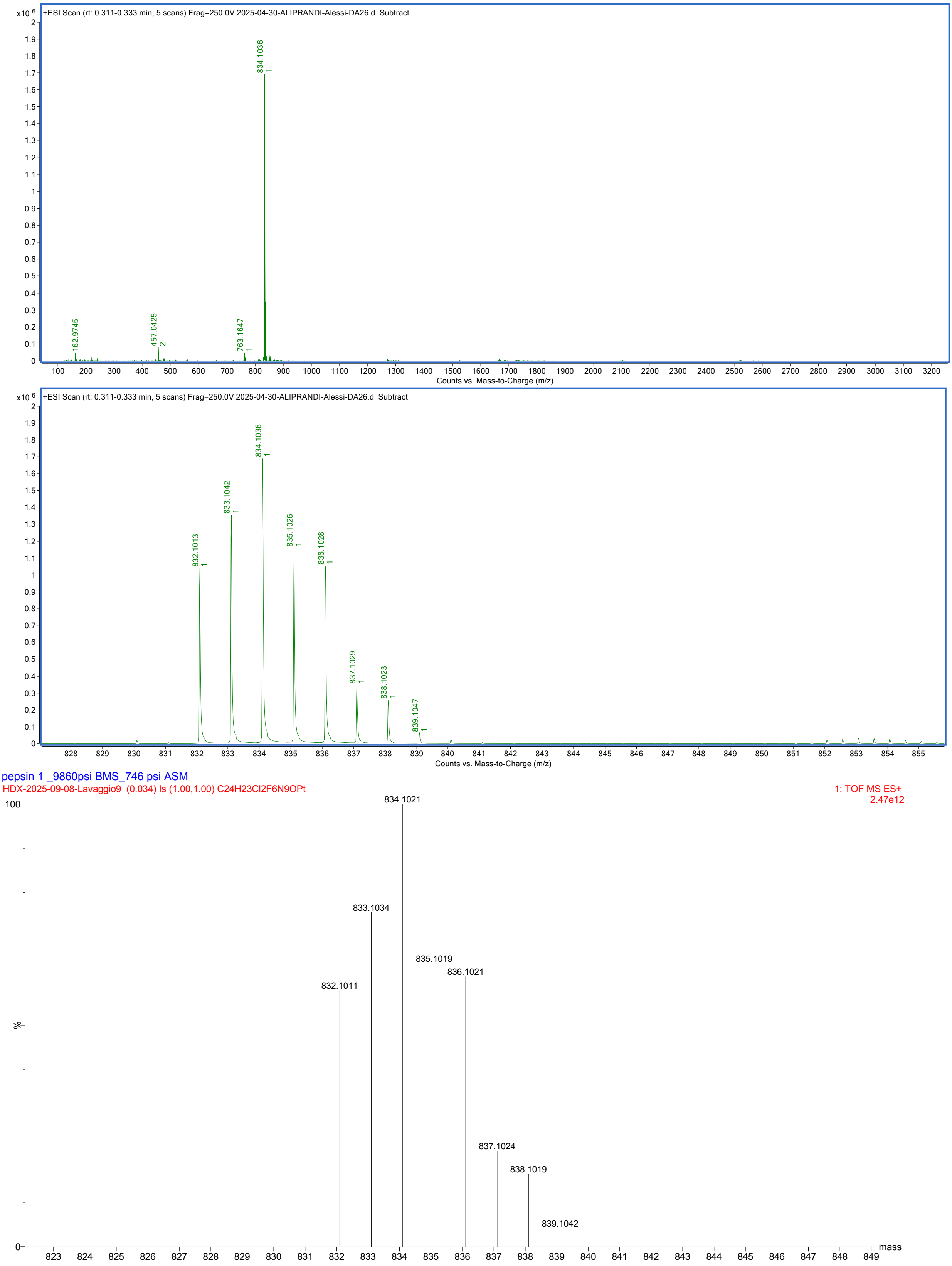

Figure S19: HRMS of compound **3a** (positive mode); top: experimental Total-Ion Counting (TIC); middle: experimental isotopic distribution of molecular ion; bottom: calculated isotopic distribution of molecular ion.

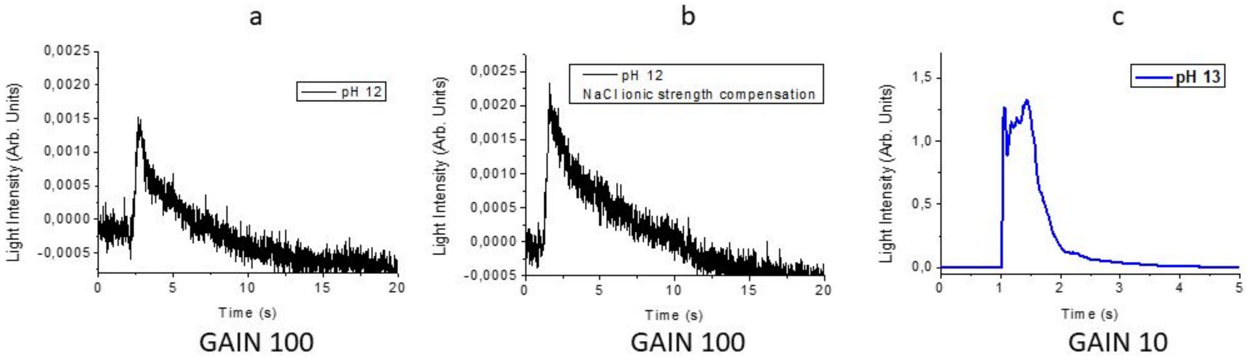

Figure S20: Chemiluminescence profiles of compound **3a** under different conditions. **a)** signal obtained by reaction between 100 μL of compound **3a** in ACN (0.02 M) and 500 μL of a 0.04 M solution of sodium ascorbate at pH 12 (NaOH), GAIN 100; **b)** chemiluminescence experiment in the same condition of point **a**, pH 12 (NaOH), and correction of ionic strength to reach the one at pH 13, GAIN 100; **c)** reference experiment with the same condition as point **a** at pH 13, GAIN 10.

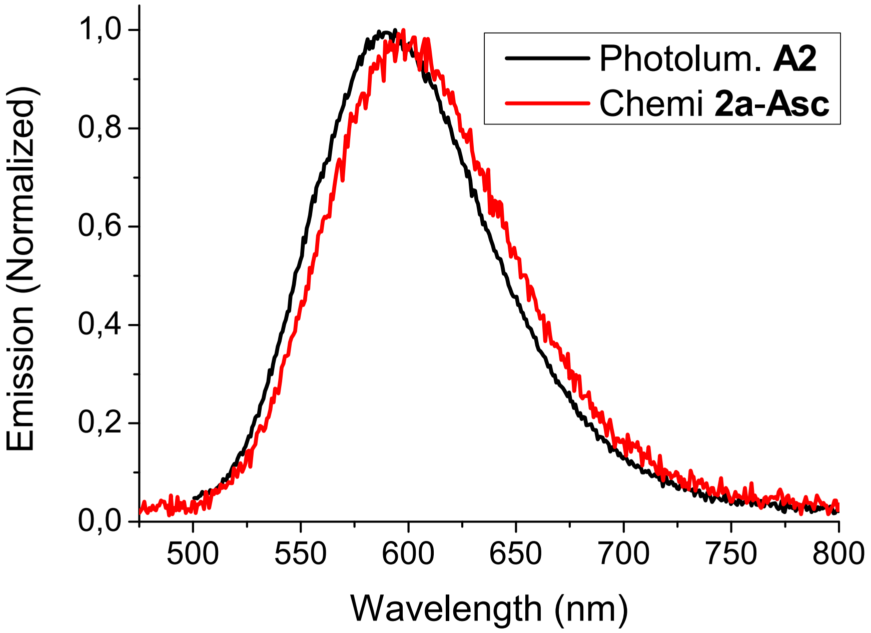

Figure S21: Photoluminescence spectrum of aggregates **A2-Asc** and chemiluminescence emission spectrum obtained from exergonic reduction of **2a**. Initial conditions for generation of **A2-Asc** and chemiluminescence: 100 μL, **2a** 0.02 M of in ACN, addition of 500 μL Na Ascorbate aq. solution 0.04 M at pH 13 (NaOH).

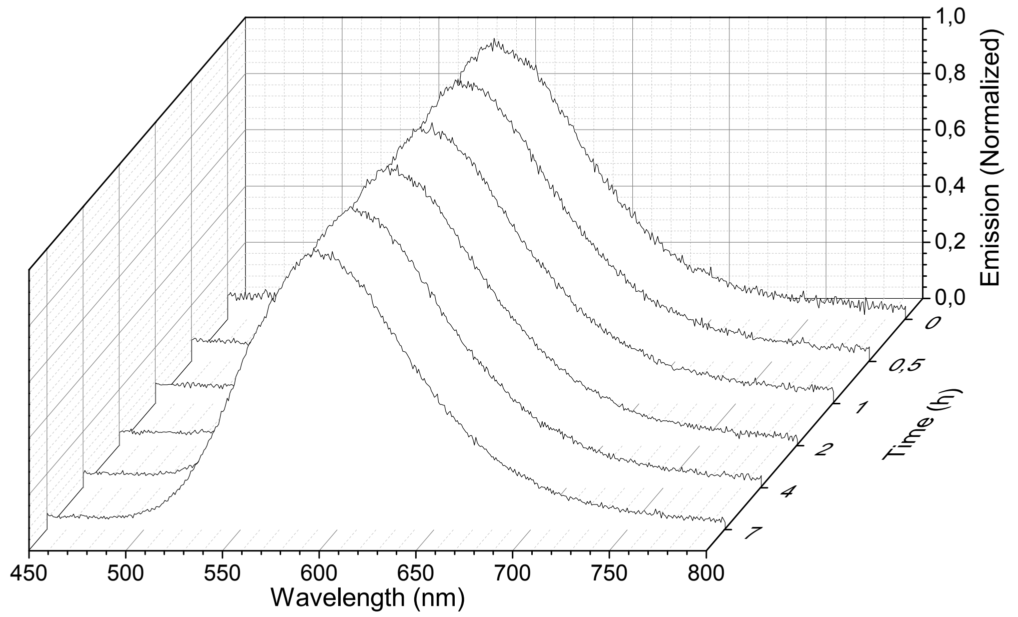

Figure S22: Emission spectra of A3 obtained by reaction between 100 µL of compound **3a** in ACN (0.02 M) and 500 µL of a 0.04 M solution of sodium ascorbate at pH 13 (NaOH) across 7 h observation period.

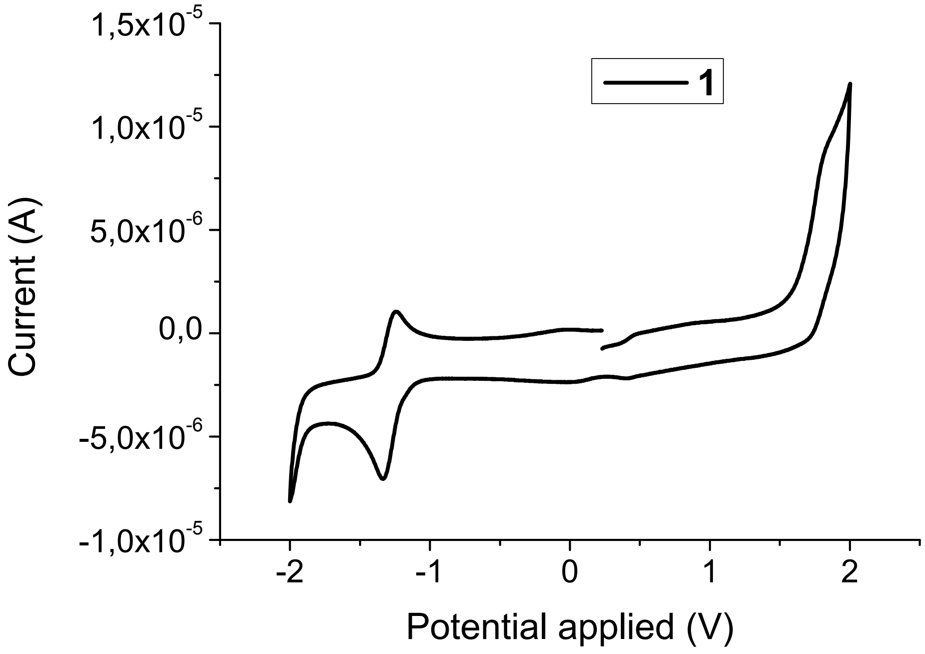

Figure S23: CV of comp. **1** (10^{-3} M in ACN) using $TBAPF_6$ (0.1 M) as supporting electrolyte and Ag wire as reference electrode; the potential of ferrocene, used as standard, is 0.47 V. Applied potential ranging from 2 V to -2 V (Ox-Red cycle), scan speed 0.1 V/sec.

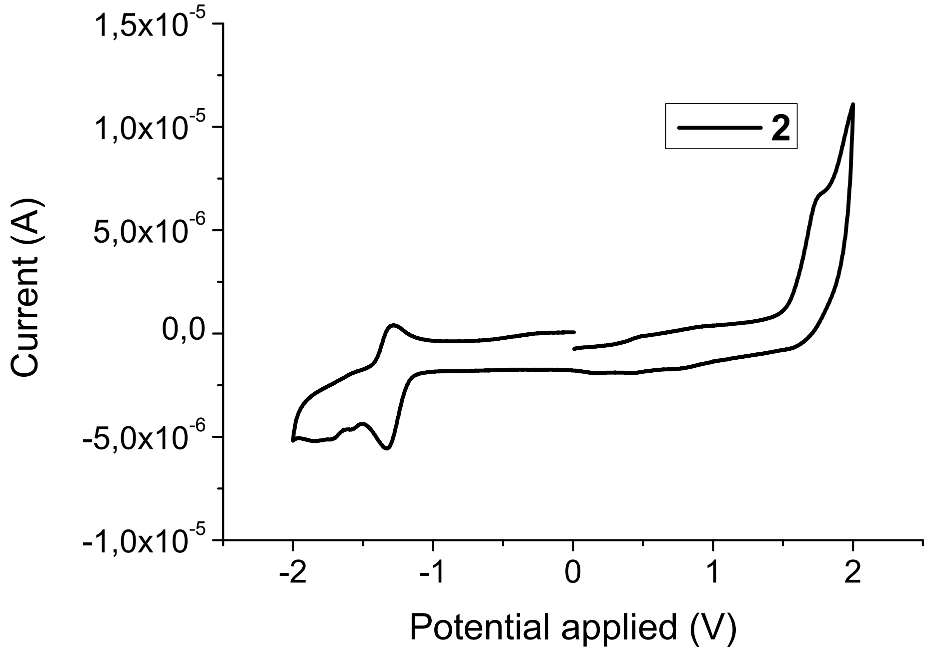

Figure S24: CV of comp. **2** (10^{-3} M in ACN) using $TBAPF_6$ (0.1 M) as supporting electrolyte and Ag wire as reference electrode; the potential of ferrocene, used as standard, is 0.47 V. Applied potential ranging from 2 V to -2 V (Ox-Red cycle), scan speed 0.1 V/sec.

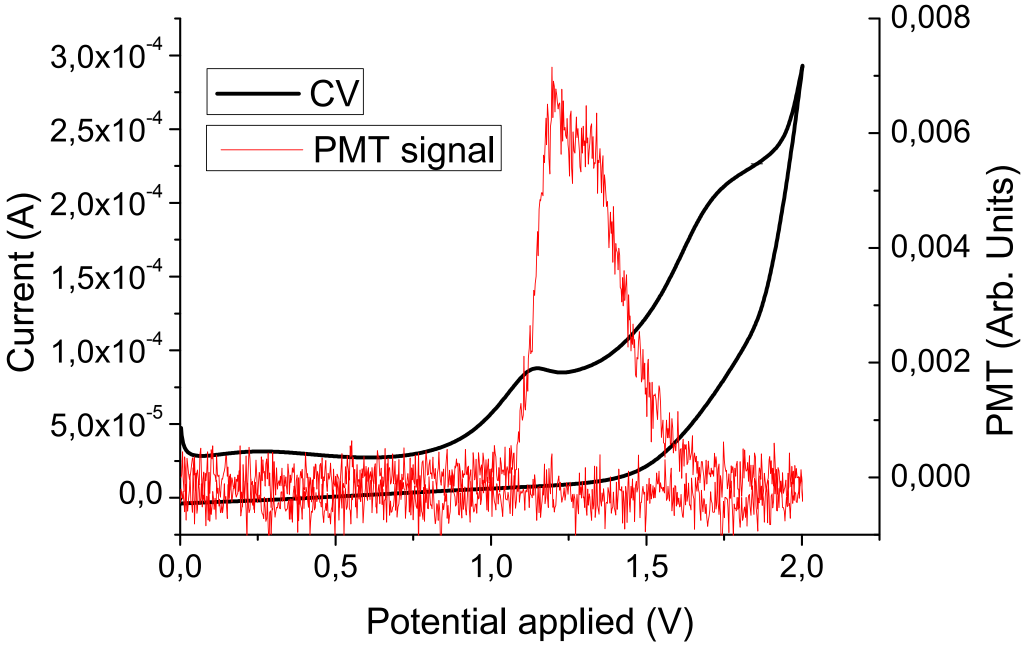

Figure S25: concentration of **2** (10^{-3} M), $TBAPF_6$ (0.1 M, supporting electrolyte) and sodium ascorbate ($5*10^{-3}$ M, pH 13, NaOH) in DMF/H_2O 2:1 is analyzed via CV/PMT method. The applied potential ranges from 0 to 2 V (reference electrode Ag wire). Black line: potential (V) vs current (A). Red line: potential (V) vs light emitted (Arb. Units)

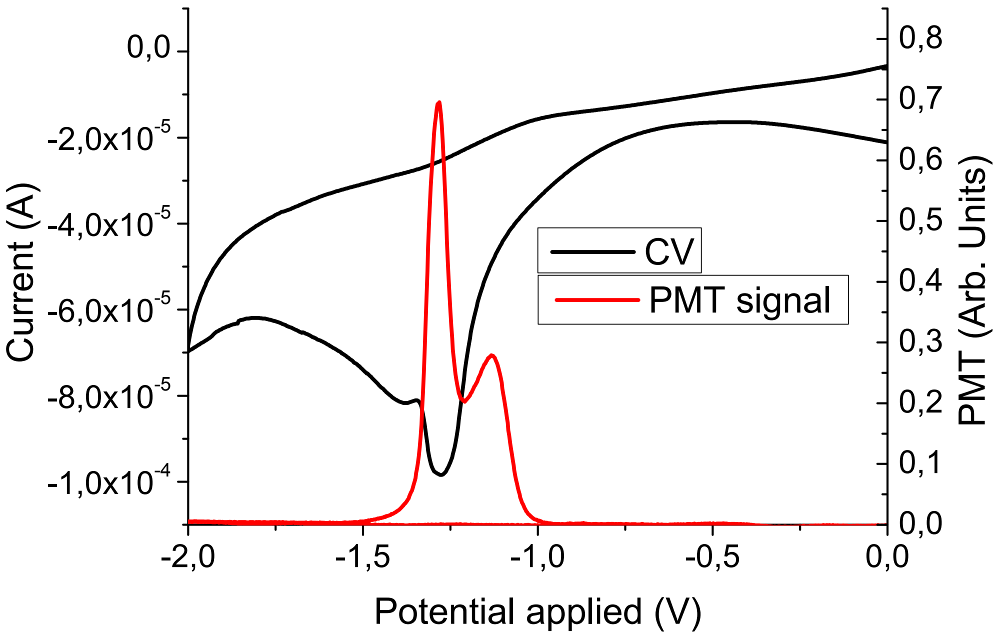

Figure 26: concentration of **2** (10^{-3} M), $TBAPF_6$ (0.1 M, supporting electrolyte) and $PhICl_2$ ($5*10^{-3}$ M, pH 13, NaOH) in DMF/H_2O 1:1 is analyzed via CV/PMT method. The applied potential ranges from 0 to -2 V (reference electrode Ag wire). Black line: potential (V) vs current (A). Red line: potential (V) vs light emitted (Arb. Units)

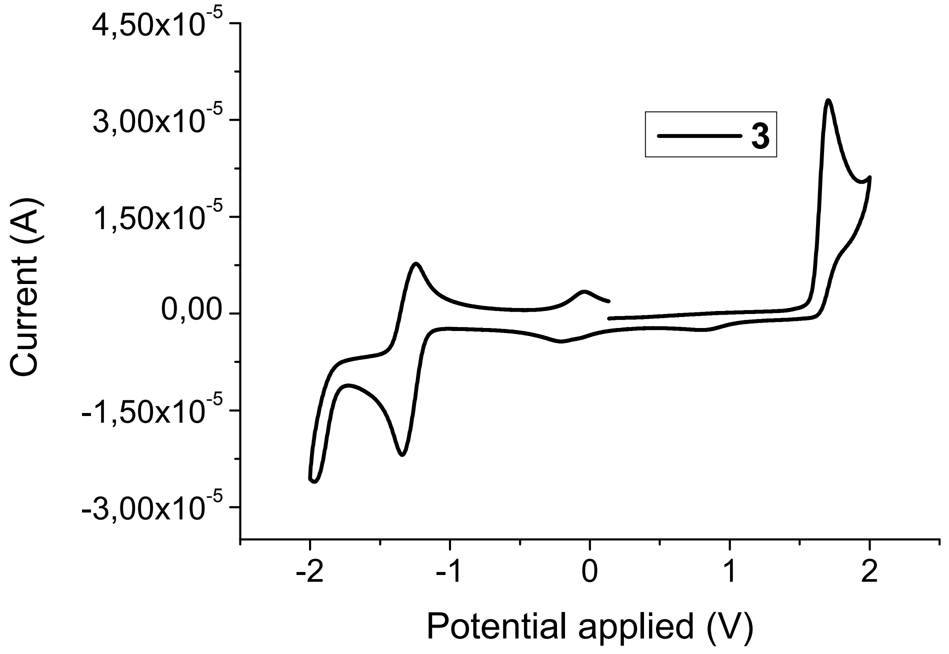

Figure S27: CV of comp. **3** (10^{-3} M in ACN) using $TBAPF_6$ (0.1 M) as supporting electrolyte and Ag wire as reference electrode; the potential of ferrocene, used as standard, is 0.47 V. Applied potential ranging from 2 V to -2 V (Ox-Red cycle), scan speed 0.1 V/sec.

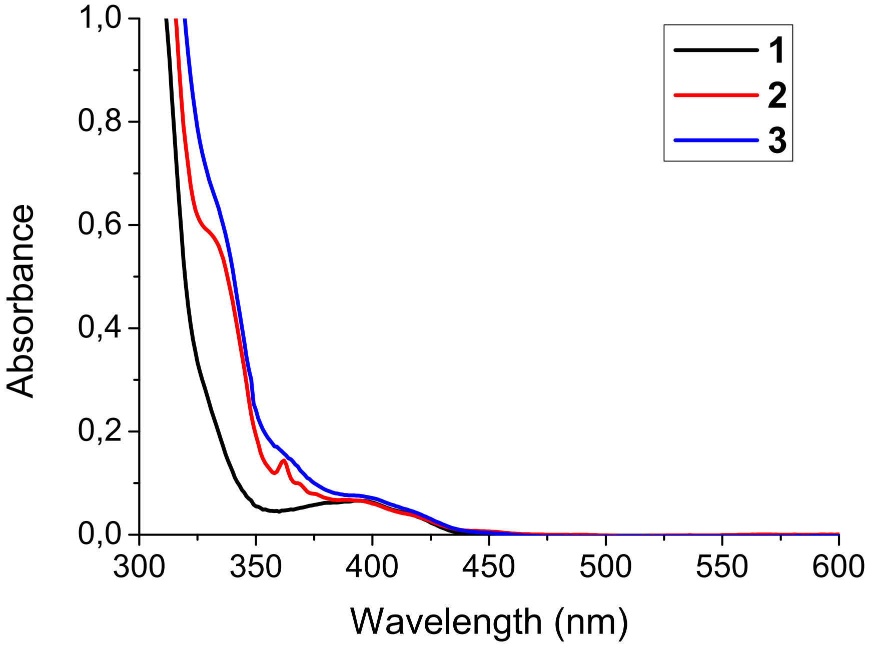

Figure S28: Uv-Vis spectrum of complex **1-3** dissolved in ACN at 10^{-4} M concentration

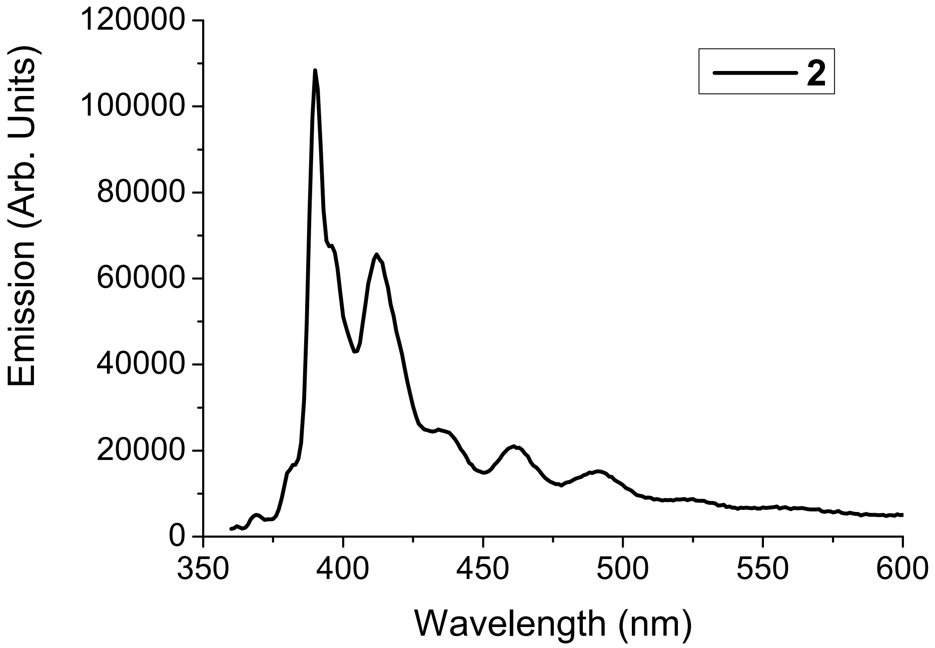

Figure S29: Emission spectrum of compound **2** (10^{-4} M in ACN), λ_{exc} = 350 nm

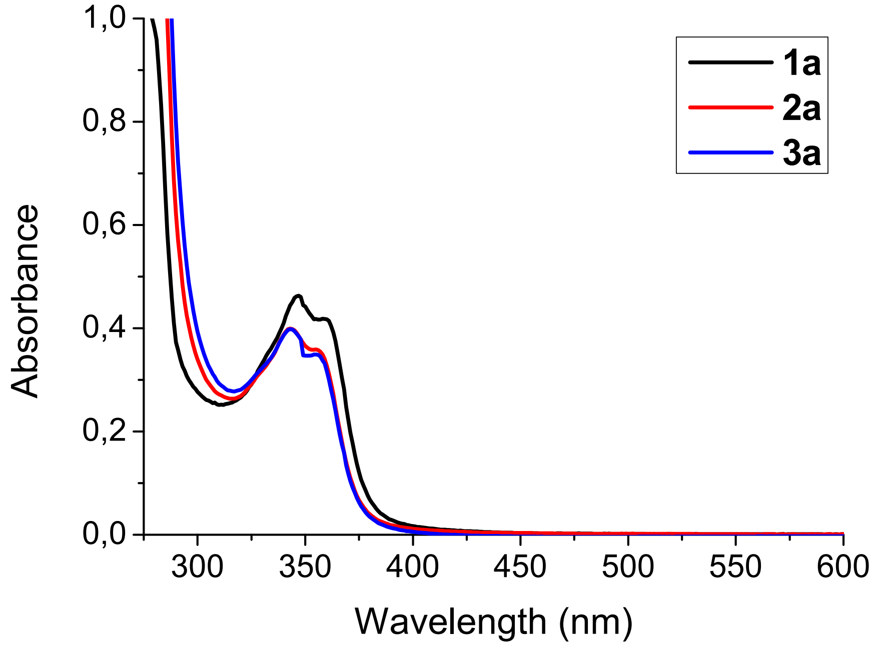

Figure S30: Uv-Vis spectrum of complex **1a-3a** dissolved in ACN at 10^{-4} M concentration

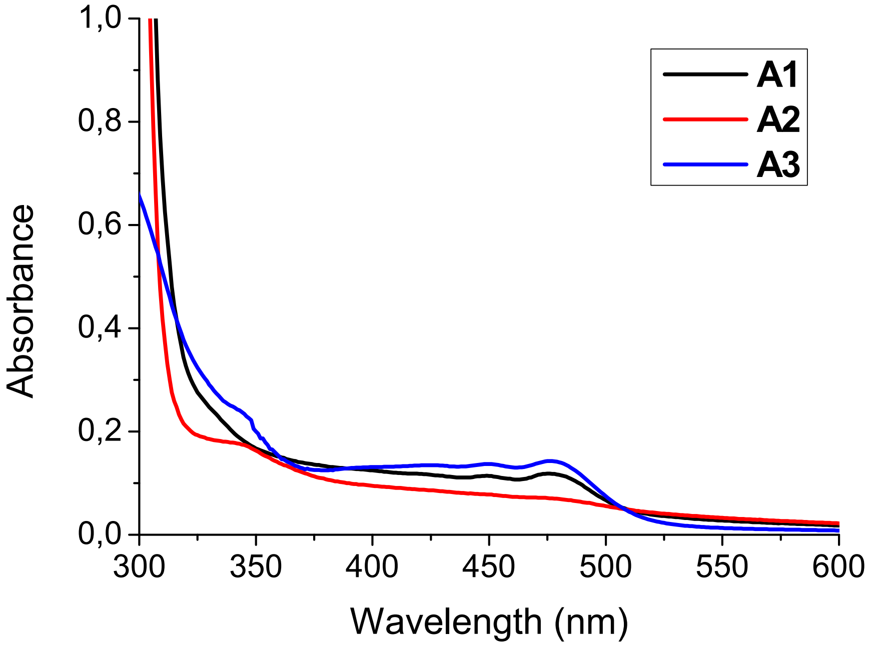

Fig. S31. Absorption spectrum of gel and aggregates **A1**-**A3** in ACN obtained by chemiluminescent reaction. Initial condition: 75 µL, 0.02 M of **1a**-**3a** in ACN, addition of 75 µL of Na Ascorbate aq. solution at 0.04 M concentration at pH 13 (NaOH).

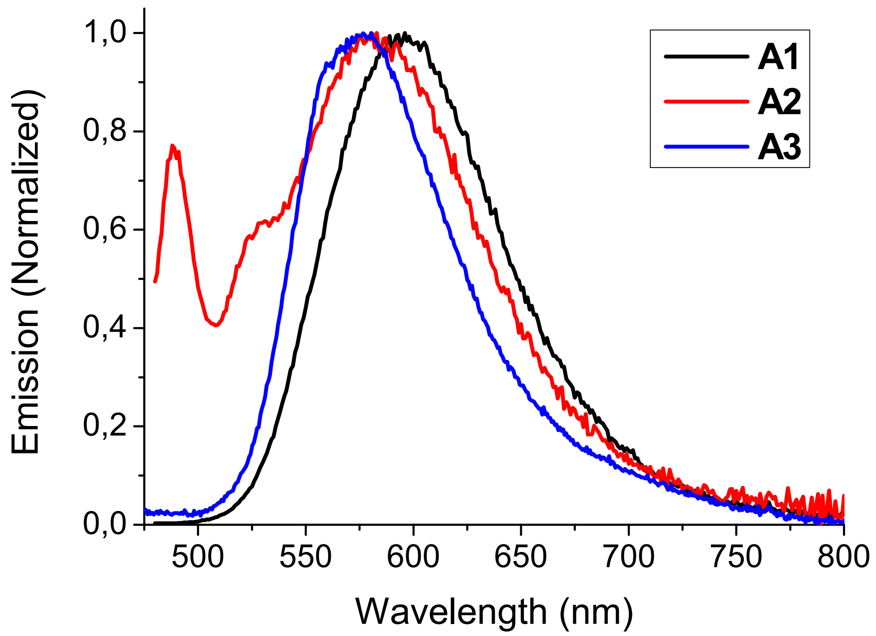

Figure S32: Emission spectrum of aggregates **A1**-**A3** in ACN-H_2O obtained by reduction reaction. Initial condition: 75 µL, 0.02 M of **1a**-**3a** in ACN, addition of 75 µL of sodium ascorbate aq. solution at 0.04 M concentration with no additional base. λ_{exc} = 470 nm

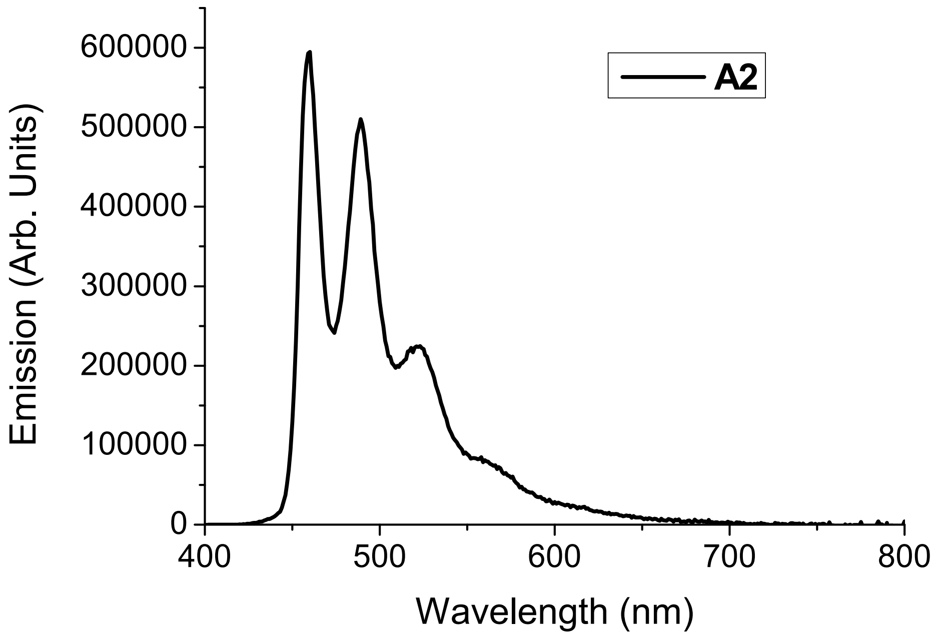

Figure S33: Emission spectrum of aggregates **A2** in ACN obtained by chemiluminescent reaction. Initial condition: 75 μL, 0.02 M of **2a** in ACN, addition of 75 μL of sodium ascorbate aq. solution at 0.04 M concentration with no additional base. λ_{exc} = 350 nm

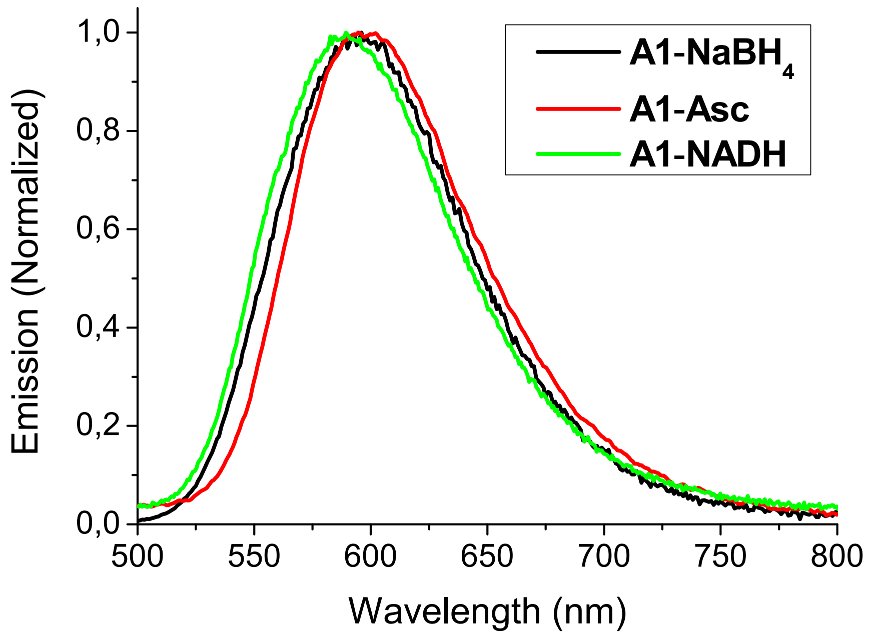

Figure S34: Emission spectrum of aggregates **A1-NaBH$_4$**, **A1-Asc**, **A1-NADH** in ACN-H_2O obtained after chemiluminescent reaction. Initial condition: 100 µL, 0.02 M of **1a** in ACN, addition of $NaBH_4$, Na Ascorbate or NADH aq. solution at 0.04 M concentration at pH 13 (NaOH).

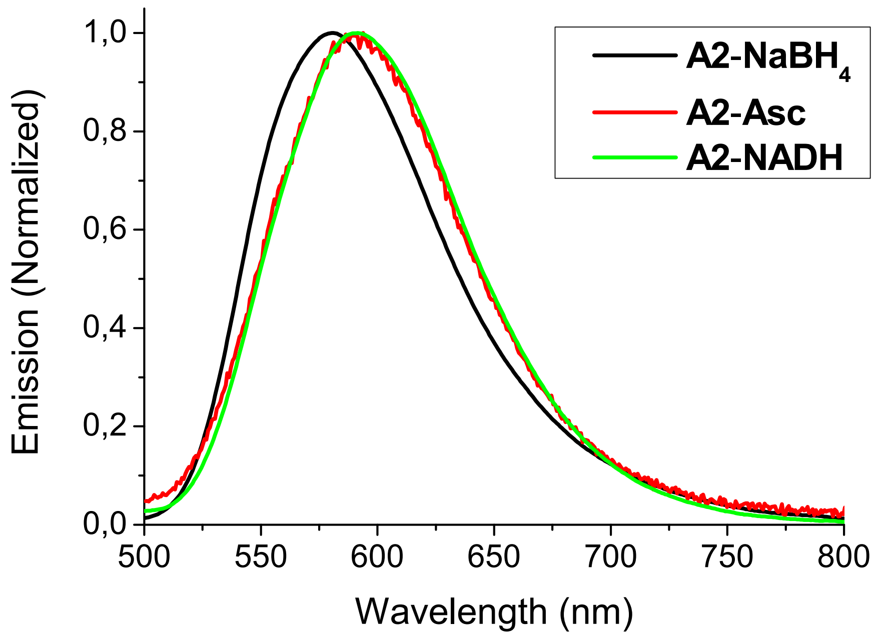

Figure S35: Emission spectrum of aggregates **A2-NaBH$_4$**, **A2-Asc** and **A2-NADH** in ACN-H_2O obtained after chemiluminescent reaction. Initial condition: 100 µL, 0.02 M of **2a** in ACN, addition of $NaBH_4$, Na Ascorbate or NADH aq. solution at 0.04 M concentration at pH 13 (NaOH).

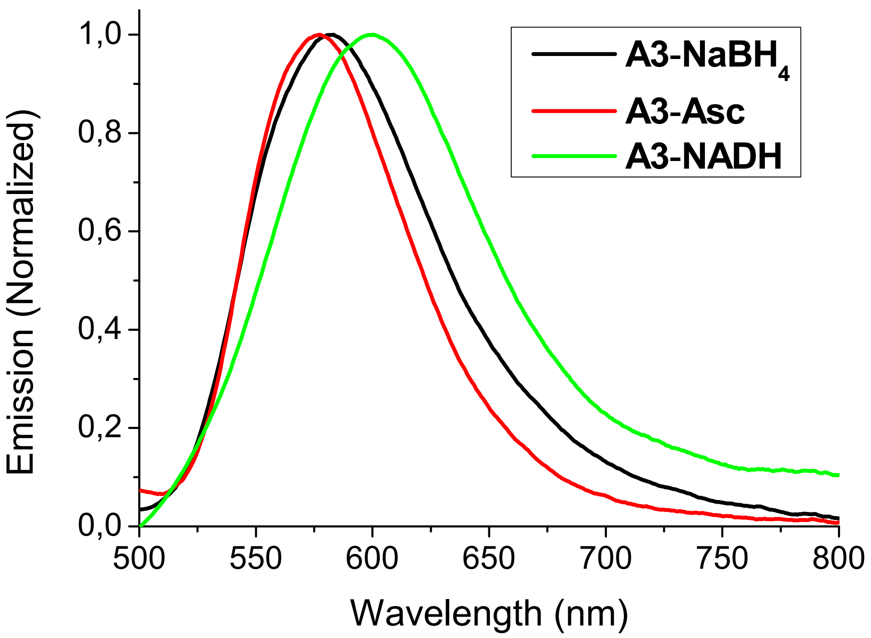

Figure S36: Emission spectrum of aggregates **A3-NaBH$_4$**, **A3-Asc** and **A3-NADH** in ACN-H_2O obtained after chemiluminescent reaction. Initial condition: 100 µL, 0.02 M of **3a** in ACN, addition of $NaBH_4$, Na Ascorbate or NADH aq. solution at 0.04 M concentration at pH 13 (NaOH).

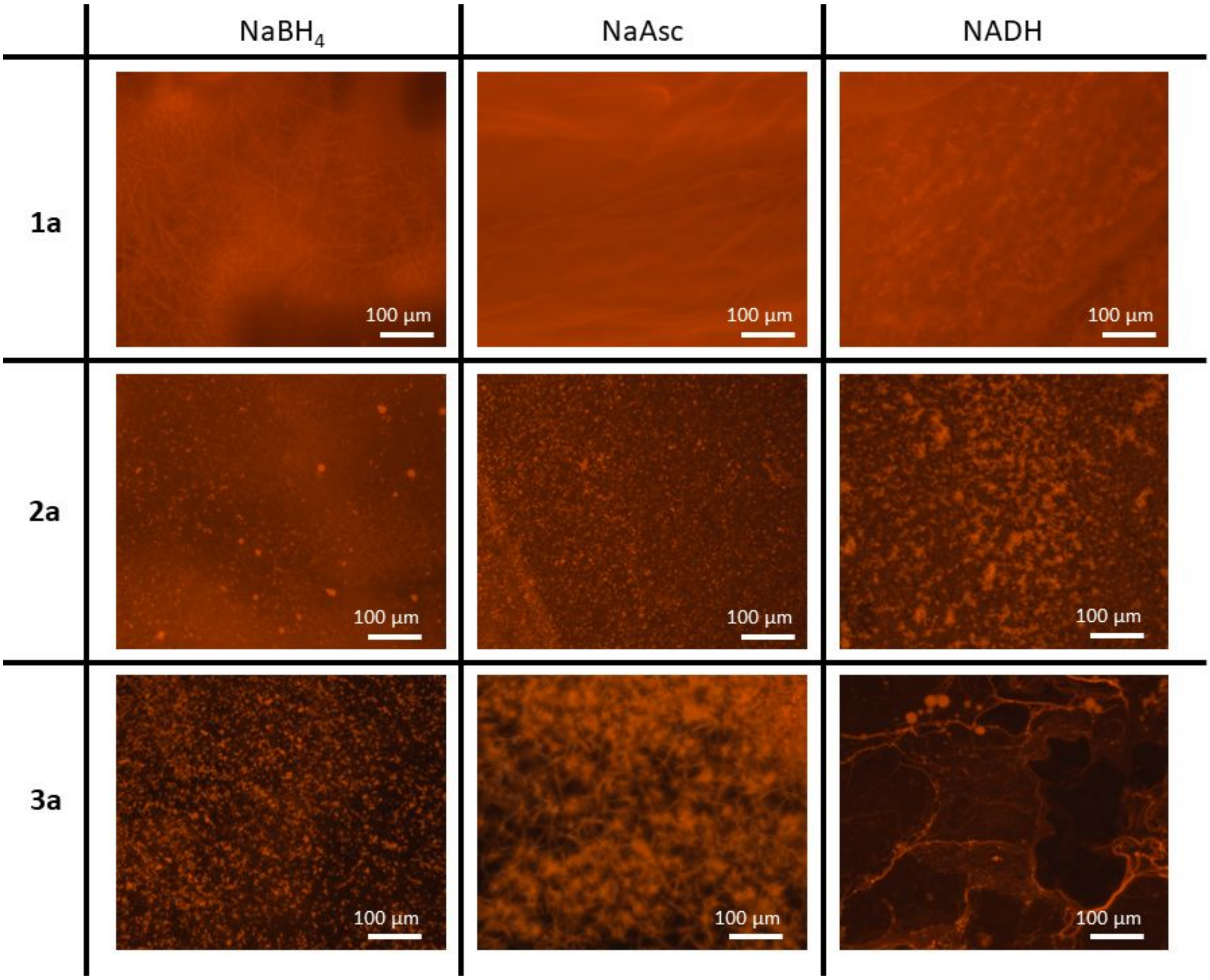

Figure S37: Fluorescence microscopy analysis of Pt(II) aggregates obtained by all combinations of Pt(IV) starting complex and reducing agents. Initial conditions for aggregates formation respects the same composition and concentration of chemiluminescence analysis on lower volume: 20 µL of metal complex (0.02 M) ACN solution, 100 µL of aq. reducing agent solution (0.04 M, pH 13). Excitation wavelength 475 nm.

Table S1. Distances and potentials of monomers and dimers (DFT calculation).

Entry	State	En. *vs* 2x ^{GS}monomer (eV)	Pt-Pt Å
1	^{GS}monomer	-	-
2	^{T1}monomer	2.64	-
3	^{GS}dimer	-1.10	3.2307
4	^{T1}dimer	1.33	3.1531
5	^{S}dimer*-A	-0.92	3.4521
6	^{S}dimer*-B	-0.78	3.3727
7	^{S}dimer*-C	-1.09	3.3739
8	^{S}dimer*-D	-0.56	4.6852
9	^{T}dimer*-A	1.49	3.4566
10	^{T}dimer*-B	1.81	4.0064
11	^{T}dimer*-C	1.34	3.4238
12	^{T}dimer*-D	1.83	4.5849

Xyz coordinates of the calculated molecules

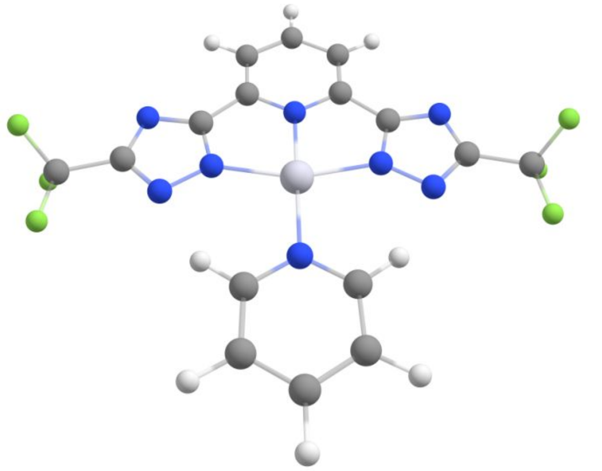

GS_monomer
SPE = -20690.9857 Hartree

Pt	-3.652737000	-2.445685000	15.793110000
N	-5.049231000	-3.410806000	16.809201000
N	-5.277425000	-1.961635000	14.724247000
N	-5.571116000	-1.248545000	13.652818000
N	-7.471374000	-2.149948000	14.457788000
N	-2.538735000	-3.282930000	17.233432000
N	-1.269743000	-3.322314000	17.595031000
N	-2.467559000	-4.561626000	19.044431000
N	-2.218563000	-1.454781000	14.749367000
C	-4.679446000	-4.119208000	17.890839000
C	-5.639431000	-4.809106000	18.626215000
H	-5.333640000	-5.379512000	19.499140000
C	-6.968267000	-4.737350000	18.205218000
H	-7.736643000	-5.268509000	18.764143000
C	-7.327521000	-3.995362000	17.078900000
H	-8.354287000	-3.923418000	16.730417000
C	-6.322580000	-3.327014000	16.384858000
C	-6.430470000	-2.497471000	15.196995000
C	-6.889272000	-1.385401000	13.524177000
C	-3.246527000	-4.032982000	18.114661000
C	-1.266042000	-4.094095000	18.679966000
C	-2.545844000	-0.735885000	13.659038000
H	-3.601709000	-0.718801000	13.378474000
C	-1.589603000	-0.048079000	12.924884000
H	-1.903611000	0.518619000	12.051652000
C	-0.256853000	-0.099520000	13.321872000
C	0.070992000	-0.845222000	14.450115000
H	1.095518000	-0.921082000	14.806295000
C	-0.931763000	-1.510779000	15.141473000
H	-0.712987000	-2.106194000	16.031120000
C	-7.660151000	-0.718665000	12.424359000
C	-0.007128000	-4.421823000	19.425696000
F	-8.535380000	0.149972000	12.918529000
F	-6.843213000	-0.066964000	11.609499000
F	-8.337697000	-1.611146000	11.712274000
F	1.031520000	-3.801641000	18.884664000
F	0.230159000	-5.728715000	19.403166000
F	-0.099621000	-4.051839000	20.697410000
H	0.511715000	0.431445000	12.762589000

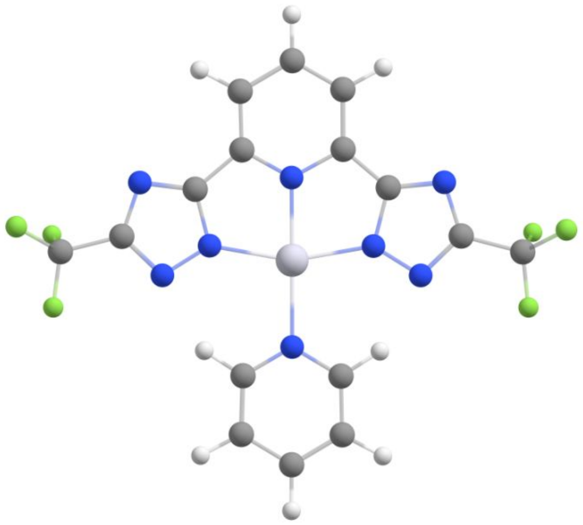

T1_monomer
SPE = -20690.8886 Hartree

Pt	-3.661697000	-2.460077000	15.809967000
N	-5.051740000	-3.407061000	16.808062000
N	-5.263305000	-1.972768000	14.743752000
N	-5.545097000	-1.257489000	13.671033000
N	-7.464666000	-2.145647000	14.459576000
N	-2.561288000	-3.269895000	17.209029000
N	-1.293035000	-3.306831000	17.563375000
N	-2.461404000	-4.565970000	19.052333000
N	-2.213956000	-1.462793000	14.755004000
C	-4.675755000	-4.141384000	17.928325000
C	-5.678756000	-4.828442000	18.652951000
H	-5.382062000	-5.401963000	19.528717000
C	-6.992879000	-4.759241000	18.241670000
H	-7.769252000	-5.283663000	18.792117000
C	-7.338562000	-3.992623000	17.082934000
H	-8.362365000	-3.913390000	16.726788000
C	-6.332416000	-3.330894000	16.394796000
C	-6.441956000	-2.507596000	15.213128000
C	-6.863873000	-1.385837000	13.534227000
C	-3.291718000	-4.059296000	18.142966000
C	-1.284152000	-4.082806000	18.655425000
C	-2.542083000	-0.743849000	13.663275000
H	-3.597320000	-0.725920000	13.383570000
C	-1.590070000	-0.056069000	12.924617000
H	-1.910061000	0.508566000	12.052108000
C	-0.254689000	-0.103679000	13.314315000
C	0.076005000	-0.848050000	14.442458000
H	1.101626000	-0.923172000	14.795927000
C	-0.923222000	-1.513740000	15.138499000
H	-0.697301000	-2.106252000	16.026746000
C	-7.619891000	-0.712354000	12.425704000
C	-0.005103000	-4.392073000	19.384946000
F	-8.496068000	0.155904000	12.915855000
F	-6.791057000	-0.061435000	11.623402000
F	-8.289659000	-1.602750000	11.705736000
F	1.020380000	-3.781505000	18.813021000
F	0.225326000	-5.697682000	19.379170000
F	-0.084009000	-3.993038000	20.646365000

H	0.510857000	0.427475000	12.751248000

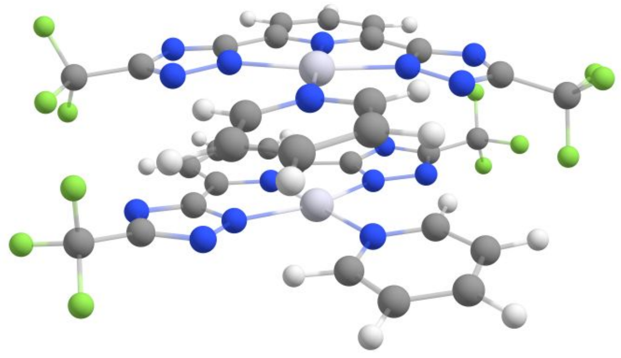

GS_dimer
(Pt–Pt = 3.2307 Å)
SPE = -41382.01186 Hartree

Pt	-5.057060000	13.272675000	4.795955000
N	-4.938142000	12.250736000	3.105948000
N	-5.015896000	14.772259000	3.468497000
N	-5.062411000	15.387777000	1.339479000
N	-5.145691000	16.085406000	3.478654000
N	-5.097881000	11.398840000	5.502382000
N	-5.183875000	9.230035000	5.047108000
N	-5.285027000	10.796558000	6.660444000
N	-5.131371000	14.323928000	6.533427000
F	-6.379163000	17.929497000	0.890308000
F	-4.267968000	18.247438000	1.068969000
F	-5.549481000	18.647458000	2.746460000
F	-5.475662000	7.259675000	6.960798000
F	-5.035060000	8.686325000	8.512835000
F	-7.008018000	8.586238000	7.679723000
C	-4.946938000	10.907387000	3.168942000
C	-4.924825000	10.157153000	1.997287000
H	-4.991684000	9.074828000	2.058554000
C	-4.874832000	10.841684000	0.783157000
H	-4.863452000	10.277404000	-0.147423000
C	-4.858154000	12.237017000	0.742263000
H	-4.830854000	12.792690000	-0.191061000
C	-4.902265000	12.931675000	1.947399000
C	-4.966256000	14.365461000	2.175908000
C	-5.175864000	16.417380000	2.190449000
C	-5.042011000	10.444828000	4.541168000
C	-5.336011000	9.502339000	6.346408000
C	-5.285024000	15.658868000	6.513531000
H	-5.352296000	16.137967000	5.536360000
C	-5.350763000	16.406414000	7.682155000
H	-5.503298000	17.479078000	7.600226000
C	-5.233566000	15.760950000	8.906918000
C	-5.068954000	14.377304000	8.918181000
H	-4.979756000	13.820406000	9.847644000
C	-5.031189000	13.687836000	7.716400000
H	-4.948484000	12.600367000	7.684562000
C	-5.347490000	17.832519000	1.725929000
C	-5.703822000	8.486269000	7.381956000
Pt	-8.286959000	13.287102000	4.723745000
N	-8.334318000	13.565587000	2.765210000
N	-8.260184000	15.288534000	4.642923000
N	-8.141273000	17.106579000	3.378263000
N	-8.113715000	16.298013000	5.478948000
N	-8.290926000	11.387547000	4.086561000
N	-8.168786000	9.989049000	2.370971000
N	-8.170808000	10.184051000	4.614079000
N	-8.291754000	12.998997000	6.735528000
F	-6.425648000	18.748604000	5.630245000
F	-8.438870000	18.973057000	6.332912000
F	-7.902885000	19.668621000	4.366905000
F	-6.853729000	7.486766000	2.997318000
F	-7.748936000	7.545723000	4.958233000
F	-8.970942000	7.242050000	3.217783000
C	-8.332784000	12.486105000	1.964175000
C	-8.326211000	12.654846000	0.582714000
H	-8.323083000	11.778701000	-0.059928000
C	-8.298931000	13.955430000	0.075853000
H	-8.270282000	14.111353000	-1.000888000
C	-8.288979000	15.060113000	0.927209000
H	-8.214644000	16.081104000	0.564409000
C	-8.317266000	14.826817000	2.298675000
C	-8.271602000	15.789471000	3.383561000
C	-8.041615000	17.366300000	4.685234000
C	-8.289148000	11.256174000	2.736906000
C	-8.094282000	9.375117000	3.560307000
C	-8.432110000	14.046471000	7.570549000
H	-8.501863000	15.034251000	7.112418000
C	-8.449067000	13.882274000	8.946730000
H	-8.569078000	14.757784000	9.580042000
C	-8.298911000	12.605427000	9.484152000
C	-8.140638000	11.533033000	8.615112000
H	-7.997113000	10.514574000	8.965466000
C	-8.152054000	11.763434000	7.245532000
H	-8.052281000	10.940008000	6.537604000
C	-7.711916000	18.712037000	5.251504000
C	-7.913023000	7.892779000	3.693740000
H	-5.274078000	16.323923000	9.838145000
H	-8.301192000	12.451792000	10.562167000

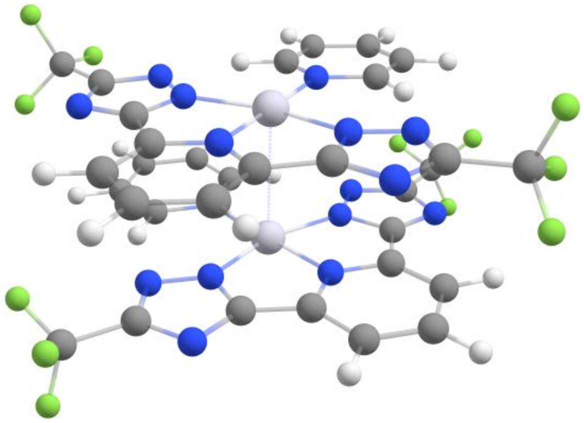

T_1_dimer
(Pt–Pt = 3.1531 Å)
SPE = -41381.9226 Hartree

Pt	-5.088132000	13.297325000	4.775550000
N	-5.072493000	11.838824000	3.465039000
N	-5.018263000	14.346723000	3.113172000

N	-4.979378000	14.347228000	0.859648000
N	-4.998835000	15.606833000	2.748466000
N	-5.108194000	11.717619000	5.988238000
N	-5.166416000	9.504218000	6.198801000
N	-5.114545000	11.481484000	7.284956000
N	-5.093982000	14.812163000	6.148762000
F	-5.562133000	16.682761000	-0.552124000
F	-3.644167000	17.116443000	0.308497000
F	-5.429712000	17.839757000	1.257823000
F	-3.941613000	8.892765000	8.912544000
F	-5.384022000	10.287197000	9.682134000
F	-6.035218000	8.470599000	8.728658000
C	-5.124216000	10.567439000	3.895951000
C	-5.157544000	9.514860000	2.986926000
H	-5.230199000	8.496749000	3.358360000
C	-5.115180000	9.812509000	1.595372000
H	-5.159771000	9.002751000	0.873609000
C	-5.067443000	11.121304000	1.168037000
H	-5.057096000	11.379656000	0.112140000
C	-5.059049000	12.166873000	2.121973000
C	-5.011642000	13.558942000	1.938283000
C	-4.982198000	15.558149000	1.404786000
C	-5.139506000	10.510432000	5.339490000
C	-5.154117000	10.151395000	7.368935000
C	-5.053688000	16.098560000	5.755705000
H	-4.997388000	16.286405000	4.683401000
C	-5.103444000	17.145741000	6.663524000
H	-5.096793000	18.165446000	6.289840000
C	-5.211849000	16.868154000	8.021613000
C	-5.247133000	15.536127000	8.420204000
H	-5.326922000	15.256531000	9.467993000
C	-5.184748000	14.534707000	7.462682000
H	-5.203154000	13.480645000	7.743317000
C	-4.905685000	16.820574000	0.592475000
C	-5.127907000	9.442500000	8.689812000
Pt	-8.239694000	13.251127000	4.690372000
N	-8.209003000	14.077115000	2.897990000
N	-8.241035000	15.193996000	5.183925000
N	-8.183596000	17.304365000	4.503300000
N	-8.275727000	15.924797000	6.282670000
N	-8.275169000	11.613081000	3.539157000
N	-8.273557000	10.756154000	1.486648000
N	-8.290664000	10.305012000	3.695459000
N	-8.298243000	12.399192000	6.536262000
F	-7.373760000	19.259114000	6.452540000
F	-8.104413000	17.975349000	8.018310000
F	-9.481365000	18.956854000	6.692665000
F	-7.621458000	8.051531000	1.093792000
F	-7.852823000	7.669052000	3.199692000
F	-9.581455000	7.948245000	1.957704000
C	-8.211381000	13.266323000	1.815059000
C	-8.177326000	13.822825000	0.538074000
H	-8.173093000	13.166703000	-0.327717000
C	-8.119416000	15.208571000	0.419816000
H	-8.058166000	15.662626000	-0.565847000
C	-8.110237000	16.026366000	1.556829000
H	-8.050475000	17.109086000	1.494015000
C	-8.164877000	15.418992000	2.806204000
C	-8.185406000	16.036284000	4.120408000
C	-8.235985000	17.178344000	5.834642000
C	-8.258752000	11.875853000	2.200602000
C	-8.284362000	9.828908000	2.447484000
C	-8.271839000	13.166749000	7.640962000
H	-8.263044000	14.247797000	7.488080000
C	-8.257384000	12.614509000	8.913629000
H	-8.228979000	13.278336000	9.774451000
C	-8.273980000	11.231516000	9.059556000
C	-8.315487000	10.446950000	7.911924000
H	-8.305571000	9.361987000	7.963223000
C	-8.320994000	11.060250000	6.667902000
H	-8.329228000	10.473380000	5.747803000
C	-8.301320000	18.354220000	6.759955000
C	-8.336230000	8.356573000	2.171918000
H	-5.281182000	17.673647000	8.749606000
H	-8.241931000	10.772105000	10.045257000

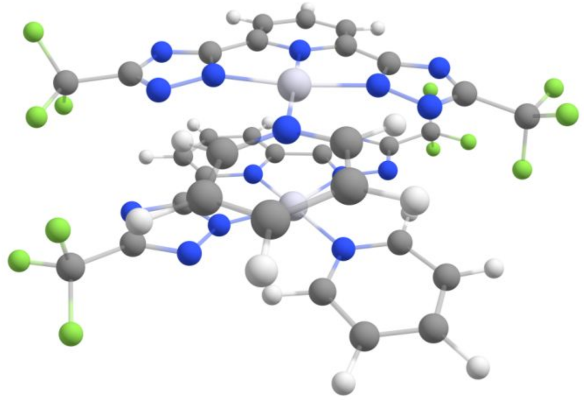

S_dimer-A
(Pt–Pt = 3.4521 Å)
SPE = -41382.00532 Hartree

Pt	-4.938791000	13.282687000	4.779940000
N	-4.813491000	12.265800000	3.112840000
N	-4.908041000	14.756064000	3.489925000
N	-4.931243000	15.406249000	1.350691000
N	-5.021037000	16.071952000	3.512824000
N	-5.016873000	11.460579000	5.494805000
N	-5.114228000	9.264433000	5.079998000
N	-5.188892000	10.877631000	6.668101000
N	-4.999205000	14.354846000	6.532582000
F	-6.128453000	17.979216000	0.889971000
F	-4.032151000	18.270876000	1.240525000
F	-5.438668000	18.635430000	2.823760000
F	-5.255610000	7.354845000	7.074972000
F	-4.968778000	8.866130000	8.580323000
F	-6.900881000	8.598046000	7.683197000
C	-4.873776000	10.895733000	3.164599000
C	-4.873028000	10.156126000	1.991008000
H	-4.962557000	9.074102000	2.053062000
C	-4.805758000	10.826345000	0.755531000
H	-4.804267000	10.259840000	-0.171581000
C	-4.784160000	12.232150000	0.726770000
H	-4.765416000	12.784216000	-0.210536000
C	-4.804146000	12.941518000	1.920517000
C	-4.858049000	14.358014000	2.148025000
C	-5.038373000	16.419493000	2.228936000
C	-4.981208000	10.452443000	4.523671000
C	-5.246189000	9.578286000	6.376763000

C	-5.182102000	15.690730000	6.521887000
H	-5.293796000	16.170389000	5.550507000
C	-5.214937000	16.442952000	7.688545000
H	-5.388643000	17.512658000	7.604882000
C	-5.039976000	15.810924000	8.915101000
C	-4.854479000	14.429277000	8.921867000
H	-4.719016000	13.876445000	9.848727000
C	-4.846066000	13.734161000	7.722209000
H	-4.741517000	12.649877000	7.700735000
C	-5.166417000	17.853777000	1.794748000
C	-5.585279000	8.575179000	7.441718000
Pt	-8.390050000	13.258854000	4.708524000
N	-8.444920000	13.543438000	2.751913000
N	-8.354034000	15.264622000	4.632258000
N	-8.196585000	17.084179000	3.369707000
N	-8.203890000	16.274176000	5.471520000
N	-8.384166000	11.362027000	4.062835000
N	-8.302394000	9.964609000	2.341478000
N	-8.243053000	10.156355000	4.584585000
N	-8.410725000	12.958942000	6.722863000
F	-6.515647000	18.728404000	5.661536000
F	-8.547098000	18.965377000	6.304519000
F	-7.953320000	19.641753000	4.349063000
F	-6.925793000	7.480614000	2.954994000
F	-7.802828000	7.527305000	4.923069000
F	-9.036953000	7.196629000	3.193837000
C	-8.467931000	12.465861000	1.944755000
C	-8.497996000	12.638395000	0.562769000
H	-8.517810000	11.763622000	-0.081961000
C	-8.482439000	13.940423000	0.057619000
H	-8.492894000	14.098843000	-1.019352000
C	-8.435125000	15.042473000	0.913073000
H	-8.377946000	16.064395000	0.547743000
C	-8.422945000	14.807190000	2.285419000
C	-8.347447000	15.767502000	3.372299000
C	-8.111428000	17.342524000	4.679351000
C	-8.413511000	11.231966000	2.712650000
C	-8.193560000	9.349015000	3.527078000
C	-8.552531000	14.002503000	7.564399000
H	-8.598465000	14.994674000	7.112367000
C	-8.611099000	13.830409000	8.940049000
H	-8.733921000	14.704743000	9.575058000
C	-8.506405000	12.547720000	9.474223000
C	-8.342082000	11.478256000	8.600702000
H	-8.230566000	10.455374000	8.951076000
C	-8.305363000	11.717251000	7.232645000
H	-8.195327000	10.896580000	6.521749000
C	-7.790810000	18.691586000	5.247311000
C	-7.987813000	7.868658000	3.658192000
H	-5.052483000	16.378495000	9.844268000
H	-8.547529000	12.387102000	10.550585000

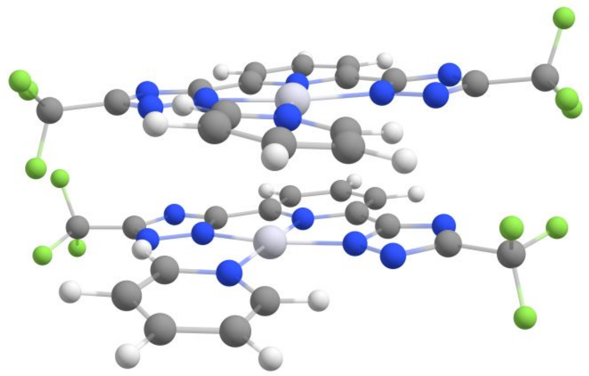

S_dimer-B
(Pt–Pt = 3.3727 Å)
SPE = -41381.99996 Hartree

Pt	-4.332623000	0.924189000	16.630945000
N	-5.449657000	0.091671000	18.012357000
N	-6.117818000	1.251300000	15.862250000
N	-6.634140000	1.830257000	14.794565000
N	-8.329192000	1.041830000	16.066389000
N	-2.936308000	0.327429000	17.892318000
N	-1.624145000	0.344481000	17.967916000
N	-2.421778000	-0.763618000	19.770090000
N	-3.183032000	1.836615000	15.204715000
C	-4.849332000	-0.491914000	19.084633000
C	-5.626154000	-1.097160000	20.067707000
H	-5.131043000	-1.564428000	20.916070000
C	-7.024594000	-1.081914000	19.935958000
H	-7.646503000	-1.537561000	20.702543000
C	-7.618275000	-0.473278000	18.819604000
H	-8.697206000	-0.442527000	18.686176000
C	-6.804667000	0.102989000	17.852815000
C	-7.168545000	0.771850000	16.630436000
C	-7.949290000	1.686891000	14.951225000
C	-3.417118000	-0.356845000	19.000111000
C	-1.354016000	-0.322183000	19.094601000
C	-3.717330000	2.213607000	14.025295000
H	-4.775908000	2.006562000	13.869782000
C	-2.968464000	2.867176000	13.055514000
H	-3.454560000	3.150967000	12.124882000
C	-1.626733000	3.146428000	13.296525000
C	-1.077941000	2.741211000	14.511395000
H	-0.033923000	2.923733000	14.755393000
C	-1.878192000	2.088856000	15.437405000
H	-1.472337000	1.733440000	16.384643000
C	-8.933417000	2.167702000	13.920907000
C	0.067856000	-0.488391000	19.552113000
F	-9.976310000	2.734407000	14.511912000
F	-8.375851000	3.052199000	13.110507000
F	-9.378676000	1.152700000	13.191305000
F	0.544670000	0.668078000	20.006955000
F	0.841526000	-0.869725000	18.541384000
F	0.151473000	-1.379805000	20.519528000
Pt	-3.546464000	-2.142430000	15.467907000
N	-5.145931000	-2.834446000	16.384087000
N	-4.968162000	-1.581065000	14.185268000
N	-5.045162000	-0.947057000	13.032939000
N	-7.134660000	-1.477954000	13.694865000
N	-2.699156000	-2.981473000	17.066349000
N	-1.483368000	-3.169073000	17.549530000

N	-2.962755000	-4.169124000	18.929008000
N	-1.877807000	-1.475193000	14.506263000
C	-4.982215000	-3.507672000	17.552107000
C	-6.091810000	-4.025761000	18.211438000
H	-5.947084000	-4.578166000	19.136629000
C	-7.359505000	-3.814985000	17.659840000
H	-8.240163000	-4.211404000	18.160942000
C	-7.504356000	-3.102820000	16.460878000
H	-8.475310000	-2.924200000	16.005270000
C	-6.360429000	-2.623999000	15.827712000
C	-6.238705000	-1.898767000	14.578277000
C	-6.354426000	-0.907655000	12.770636000
C	-3.584354000	-3.591229000	17.913674000
C	-1.682970000	-3.876248000	18.662391000
C	-1.952569000	-1.036051000	13.233457000
H	-2.945294000	-1.015116000	12.780707000
C	-0.831057000	-0.608045000	12.537647000
H	-0.946558000	-0.271113000	11.510017000
C	0.411567000	-0.620578000	13.167672000
C	0.477942000	-1.059240000	14.486386000
H	1.416315000	-1.086948000	15.035558000
C	-0.680837000	-1.480858000	15.125753000
H	-0.652815000	-1.855031000	16.149501000
C	-6.862105000	-0.207240000	11.543207000
C	-0.541366000	-4.383613000	19.495753000
F	-8.109463000	-0.543277000	11.280617000
F	-6.808120000	1.116887000	11.699661000
F	-6.109853000	-0.507914000	10.488970000
F	0.593326000	-3.795690000	19.144459000
F	-0.392078000	-5.695426000	19.324690000
F	-0.753774000	-4.168004000	20.783790000
H	1.307654000	-0.295162000	12.641422000
H	-1.021683000	3.666267000	12.555364000

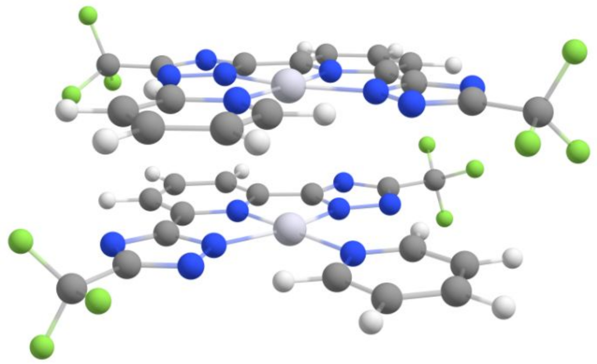

S_dimer-C
(Pt–Pt = 3.3739 Å)
SPE = -41382.01141 Hartree

Pt	-4.970559000	13.266636000	4.768042000
N	-4.908565000	11.815757000	3.449054000
N	-4.906576000	14.321360000	3.111412000
N	-4.870687000	14.331455000	0.873296000
N	-4.931199000	15.590577000	2.754008000
N	-5.005360000	11.707725000	5.967933000
N	-5.049821000	9.484396000	6.188026000
N	-5.064548000	11.473798000	7.265813000
N	-4.982661000	14.785650000	6.153439000
F	-5.591593000	16.635721000	-0.525999000
F	-3.667420000	17.153173000	0.270199000
F	-5.450227000	17.817641000	1.266930000
F	-3.987235000	8.829830000	8.928874000
F	-5.375212000	10.300334000	9.661008000
F	-6.095343000	8.518851000	8.692229000
C	-4.959488000	10.523960000	3.882805000
C	-4.980329000	9.483690000	2.961372000
H	-5.050563000	8.460553000	3.322230000
C	-4.946817000	9.784009000	1.588960000
H	-4.991958000	8.982808000	0.857040000
C	-4.898812000	11.119196000	1.167027000
H	-4.888878000	11.385526000	0.112819000
C	-4.888820000	12.133363000	2.120398000
C	-4.873598000	13.554365000	1.940780000
C	-4.916267000	15.554558000	1.420572000
C	-5.000432000	10.474634000	5.316933000
C	-5.093228000	10.144853000	7.354204000
C	-4.982588000	16.076481000	5.762637000
H	-4.968940000	16.268329000	4.689738000
C	-5.012289000	17.123538000	6.672154000
H	-5.042300000	18.143139000	6.296999000
C	-5.047611000	16.847331000	8.035268000
C	-5.039353000	15.514064000	8.433243000
H	-5.062250000	15.232531000	9.483629000
C	-5.009521000	14.512300000	7.473710000
H	-5.011809000	13.460036000	7.758932000
C	-4.907576000	16.812494000	0.596726000
C	-5.137315000	9.440017000	8.680431000
Pt	-8.343621000	13.269311000	4.691130000
N	-8.336966000	14.091833000	2.897129000
N	-8.341536000	15.213557000	5.181885000
N	-8.298444000	17.325287000	4.497705000
N	-8.330568000	15.947860000	6.281550000
N	-8.363057000	11.629865000	3.540703000
N	-8.332819000	10.766070000	1.492247000
N	-8.331660000	10.318099000	3.701700000
N	-8.412829000	12.417510000	6.542571000
F	-7.413238000	19.289028000	6.365038000
F	-8.008993000	18.015874000	7.995256000
F	-9.494114000	18.976727000	6.776151000
F	-7.640567000	8.094483000	1.066837000
F	-7.727184000	7.705772000	3.182492000
F	-9.538363000	7.889635000	2.045602000
C	-8.343411000	13.281457000	1.812598000
C	-8.328380000	13.836551000	0.535686000
H	-8.328972000	13.179618000	-0.330130000
C	-8.290688000	15.226103000	0.413169000
H	-8.250399000	15.679657000	-0.574399000
C	-8.284466000	16.044078000	1.549763000
H	-8.241268000	17.128000000	1.484325000
C	-8.313922000	15.438651000	2.801075000
C	-8.319366000	16.056539000	4.115360000
C	-8.301097000	17.200890000	5.830749000
C	-8.358609000	11.886950000	2.202914000
C	-8.311309000	9.839937000	2.457221000
C	-8.411653000	13.184056000	7.650748000
H	-8.383978000	14.265238000	7.499375000
C	-8.445320000	12.632414000	8.924516000
H	-8.441178000	13.297097000	9.785484000
C	-8.480106000	11.249132000	9.072371000

C	-8.491792000	10.464476000	7.923047000
H	-8.503702000	9.378974000	7.974703000
C	-8.454742000	11.077317000	6.678082000
H	-8.447503000	10.489010000	5.758056000
C	-8.307649000	18.382034000	6.752859000
C	-8.305740000	8.365705000	2.184312000
H	-5.094075000	17.651895000	8.766498000
H	-8.491351000	10.790906000	10.059664000

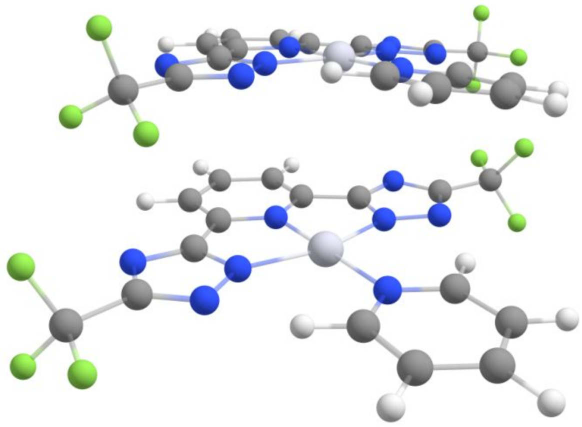

S_dimer-D
(Pt–Pt = 4.6852 Å)
SPE = -41381.99203 Hartree

Pt	-4.169822000	1.682945000	16.023465000
N	-5.458300000	0.581625000	17.040498000
N	-5.879887000	2.202908000	15.121232000
N	-6.268697000	2.994840000	14.138229000
N	-8.079541000	1.956513000	14.981642000
N	-2.923354000	0.742662000	17.290119000
N	-1.622506000	0.664979000	17.518727000
N	-2.679951000	-0.753602000	18.912401000
N	-2.853083000	2.839878000	14.988700000
C	-4.991086000	-0.220330000	18.015944000
C	-5.874174000	-1.015120000	18.741571000
H	-5.491609000	-1.660010000	19.528576000
C	-7.231230000	-0.962449000	18.414447000
H	-7.937948000	-1.582737000	18.962575000
C	-7.692935000	-0.125047000	17.395370000
H	-8.740613000	-0.074700000	17.111419000
C	-6.762789000	0.652399000	16.711655000
C	-6.980439000	1.583518000	15.616971000
C	-7.587297000	2.821017000	14.084915000
C	-3.545073000	-0.116147000	18.135813000
C	-1.517090000	-0.240205000	18.490929000
C	-3.284170000	3.642835000	13.996559000
H	-4.355716000	3.625634000	13.779460000
C	-2.414270000	4.459394000	13.285427000
H	-2.813489000	5.090643000	12.494778000
C	-1.059217000	4.455170000	13.603175000
C	-0.622450000	3.621217000	14.628968000
H	0.423913000	3.579112000	14.922792000
C	-1.542763000	2.826599000	15.300160000
H	-1.236567000	2.155518000	16.105873000
C	-8.444250000	3.499702000	13.058759000
C	-0.177303000	-0.631943000	19.043247000
F	-9.479183000	4.102964000	13.629441000
F	-7.746270000	4.408618000	12.390672000
F	-8.921527000	2.620759000	12.184486000
F	0.428820000	0.404621000	19.612090000
F	0.628104000	-1.073590000	18.073858000
F	-0.301409000	-1.587948000	19.947343000
Pt	-3.693603000	-2.977984000	16.026240000
N	-5.067193000	-3.959801000	17.017333000
N	-5.289196000	-2.435969000	15.021728000
N	-5.582498000	-1.625452000	14.016123000
N	-7.512435000	-2.501445000	14.807694000
N	-2.579941000	-3.794038000	17.408102000
N	-1.313587000	-3.776473000	17.777272000
N	-2.474042000	-5.008559000	19.281529000
N	-2.257271000	-1.936603000	14.987415000
C	-4.702636000	-4.668582000	18.134048000
C	-5.661983000	-5.335937000	18.873907000
H	-5.352731000	-5.897532000	19.752896000
C	-7.014022000	-5.269063000	18.473841000
H	-7.777367000	-5.797381000	19.038421000
C	-7.367329000	-4.501153000	17.350015000
H	-8.401470000	-4.407733000	17.024495000
C	-6.379094000	-3.838856000	16.634954000
C	-6.489427000	-2.966630000	15.498866000
C	-6.906555000	-1.686996000	13.924700000
C	-3.284408000	-4.557992000	18.351199000
C	-1.288695000	-4.502276000	18.890831000
C	-2.579095000	-1.251210000	13.869984000
H	-3.624347000	-1.274708000	13.560879000
C	-1.636872000	-0.528422000	13.152699000
H	-1.954795000	0.000893000	12.257231000
C	-0.316790000	-0.491449000	13.595021000
C	0.006105000	-1.189785000	14.754341000
H	1.011285000	-1.186030000	15.168408000
C	-0.981058000	-1.901551000	15.420764000
H	-0.750582000	-2.455780000	16.330143000
C	-7.640156000	-0.936095000	12.846705000
C	-0.018288000	-4.748846000	19.656880000
F	-8.866430000	-0.640842000	13.227513000
F	-6.995175000	0.185588000	12.547794000
F	-7.711373000	-1.670443000	11.739486000
F	1.001357000	-4.139848000	19.077718000
F	0.240461000	-6.050907000	19.705726000
F	-0.130076000	-4.308049000	20.900053000
H	0.439634000	0.071449000	13.050445000
H	-0.358268000	5.090601000	13.063821000

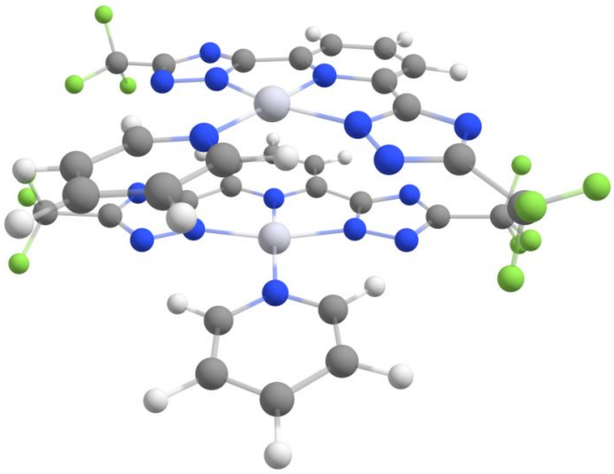

T_dimer-A
(Pt–Pt = 3.4566 Å)
SPE = -41381.91654 Hartree

Pt	-4.942848000	13.288128000	4.784514000
N	-4.831818000	12.283614000	3.108891000
N	-4.903270000	14.776890000	3.479825000
N	-4.932148000	15.422560000	1.344159000
N	-5.015679000	16.090695000	3.500817000
N	-5.024787000	11.458562000	5.489931000
N	-5.125313000	9.250658000	5.053650000
N	-5.195014000	10.862883000	6.651654000
N	-5.000726000	14.354315000	6.537177000
F	-6.136302000	17.989847000	0.871776000
F	-4.041667000	18.294933000	1.217069000
F	-5.446138000	18.652072000	2.803332000
F	-5.309359000	7.325484000	7.016984000
F	-4.933236000	8.811458000	8.526950000
F	-6.898147000	8.612071000	7.684967000
C	-4.889876000	10.901140000	3.160093000
C	-4.890709000	10.169590000	1.962179000
H	-4.980672000	9.086730000	2.016065000
C	-4.819597000	10.841761000	0.746053000
H	-4.820761000	10.285705000	-0.187317000
C	-4.786555000	12.261325000	0.729280000
H	-4.758872000	12.824264000	-0.200883000
C	-4.808856000	12.956464000	1.932464000
C	-4.855362000	14.378681000	2.155817000
C	-5.036287000	16.436657000	2.212474000
C	-4.991994000	10.452021000	4.495965000
C	-5.255300000	9.556949000	6.340497000
C	-5.170588000	15.690509000	6.522980000
H	-5.274703000	16.168914000	5.549162000
C	-5.200648000	16.444048000	7.689413000
H	-5.365010000	17.515288000	7.606387000
C	-5.034671000	15.809615000	8.915741000
C	-4.859885000	14.427110000	8.925196000
H	-4.730783000	13.874780000	9.853227000
C	-4.855256000	13.731568000	7.724808000
H	-4.759389000	12.646048000	7.700642000
C	-5.171004000	17.868539000	1.775322000
C	-5.591680000	8.550605000	7.404803000
Pt	-8.398695000	13.254392000	4.718914000
N	-8.436182000	13.532657000	2.759881000
N	-8.357417000	15.259210000	4.636455000
N	-8.190351000	17.074345000	3.369386000
N	-8.212545000	16.270643000	5.473252000
N	-8.389660000	11.354567000	4.080377000
N	-8.282377000	9.953285000	2.363856000
N	-8.251060000	10.150883000	4.606677000
N	-8.424356000	12.962452000	6.734299000
F	-6.519252000	18.724239000	5.660568000
F	-8.551412000	18.961048000	6.301345000
F	-7.955859000	19.635876000	4.345823000
F	-6.926909000	7.466749000	2.980257000
F	-7.798415000	7.519596000	4.951160000
F	-9.038365000	7.191573000	3.226241000
C	-8.452291000	12.452235000	1.957577000
C	-8.467144000	12.619595000	0.574572000
H	-8.481718000	11.742779000	-0.067534000
C	-8.443381000	13.920145000	0.065517000
H	-8.441009000	14.074814000	-1.012079000
C	-8.403546000	15.025000000	0.916784000
H	-8.337784000	16.045370000	0.548581000
C	-8.407677000	14.794127000	2.290556000
C	-8.341489000	15.758207000	3.375017000
C	-8.113471000	17.336858000	4.678658000
C	-8.403319000	11.221059000	2.730549000
C	-8.185919000	9.340699000	3.552094000
C	-8.577162000	14.008267000	7.570562000
H	-8.630495000	14.997986000	7.113994000
C	-8.634215000	13.841928000	8.947000000
H	-8.766005000	14.717607000	9.578335000
C	-8.515601000	12.562732000	9.486579000
C	-8.340602000	11.491004000	8.618127000
H	-8.218366000	10.470943000	8.973060000
C	-8.306739000	11.724287000	7.248855000
H	-8.188919000	10.901610000	6.541709000
C	-7.793440000	18.686819000	5.245181000
C	-7.984471000	7.859840000	3.686243000
H	-5.045272000	16.377802000	9.844713000
H	-8.554578000	12.406560000	10.563706000

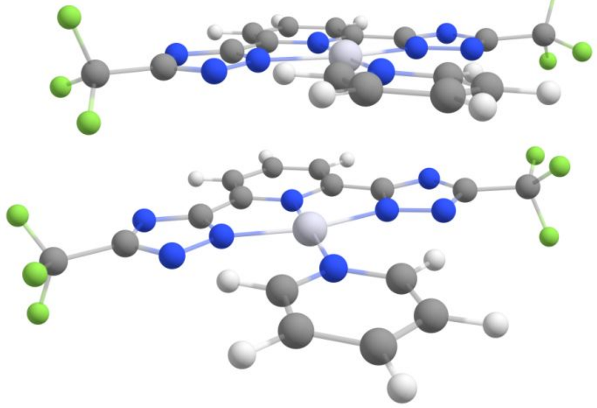

T_dimer-B
(Pt–Pt = 4.0064 Å)
SPE = -41381.90484 Hartree

Pt	-4.222982000	1.381820000	16.346085000
N	-5.509980000	0.455938000	17.488531000
N	-5.901058000	1.839042000	15.417475000

N	-6.272120000	2.508294000	14.345224000
N	-8.118305000	1.602226000	15.284626000
N	-2.994207000	0.573238000	17.645442000
N	-1.692643000	0.493092000	17.847500000
N	-2.722329000	-0.726836000	19.459478000
N	-2.881338000	2.379960000	15.157766000
C	-5.041950000	-0.261045000	18.571632000
C	-5.954763000	-0.935904000	19.390038000
H	-5.575335000	-1.488451000	20.247642000
C	-7.317136000	-0.891031000	19.091313000
H	-8.034555000	-1.408365000	19.722225000
C	-7.762324000	-0.165621000	17.954986000
H	-8.812904000	-0.116617000	17.677933000
C	-6.831389000	0.500772000	17.170560000
C	-7.042929000	1.286497000	15.985917000
C	-7.595082000	2.336868000	14.296405000
C	-3.627000000	-0.190610000	18.646913000
C	-1.579362000	-0.290149000	18.928011000
C	-3.293325000	3.022041000	14.045019000
H	-4.359954000	2.973736000	13.815890000
C	-2.414758000	3.728344000	13.234725000
H	-2.804615000	4.230408000	12.352030000
C	-1.064968000	3.786579000	13.571946000
C	-0.646043000	3.118765000	14.719459000
H	0.394401000	3.126855000	15.036656000
C	-1.573883000	2.425535000	15.484934000
H	-1.272800000	1.876105000	16.378362000
C	-8.435310000	2.984056000	13.231541000
C	-0.225062000	-0.570139000	19.520382000
F	-9.055090000	4.054232000	13.723672000
F	-7.683824000	3.376245000	12.214693000
F	-9.357570000	2.148901000	12.785625000
F	0.187000000	0.477579000	20.230387000
F	0.668951000	-0.787414000	18.564635000
F	-0.266757000	-1.619120000	20.319843000
Pt	-3.654119000	-2.543373000	15.779663000
N	-5.058444000	-3.508814000	16.785492000
N	-5.274659000	-2.068114000	14.690277000
N	-5.569292000	-1.343098000	13.626108000
N	-7.470057000	-2.256599000	14.416913000
N	-2.553201000	-3.372586000	17.234579000
N	-1.286574000	-3.410941000	17.607010000
N	-2.492050000	-4.661031000	19.039920000
N	-2.196953000	-1.591801000	14.725130000
C	-4.696282000	-4.216402000	17.871557000
C	-5.660779000	-4.916027000	18.594244000
H	-5.360530000	-5.487482000	19.468791000
C	-6.984402000	-4.858959000	18.154221000
H	-7.754292000	-5.402019000	18.700186000
C	-7.335375000	-4.120034000	17.022984000
H	-8.357643000	-4.060664000	16.658620000
C	-6.327323000	-3.439051000	16.344111000
C	-6.428368000	-2.610073000	15.154877000
C	-6.890299000	-1.474817000	13.497179000
C	-3.265601000	-4.126624000	18.108234000
C	-1.289780000	-4.186276000	18.690757000
C	-2.492523000	-0.982446000	13.560468000
H	-3.540909000	-0.990038000	13.254240000
C	-1.517209000	-0.363797000	12.790411000
H	-1.806207000	0.109190000	11.854750000
C	-0.196657000	-0.364749000	13.232656000
C	0.097005000	-0.984814000	14.442796000
H	1.107931000	-1.011866000	14.842728000
C	-0.924658000	-1.588429000	15.165275000
H	-0.725624000	-2.091839000	16.113643000
C	-7.629983000	-0.849953000	12.349415000
C	-0.024995000	-4.565708000	19.403892000
F	-8.916187000	-0.738623000	12.618729000
F	-7.142249000	0.360350000	12.079134000
F	-7.499814000	-1.582859000	11.245267000
F	0.987020000	-3.810096000	19.002801000
F	0.287295000	-5.835534000	19.150367000
F	-0.157605000	-4.440357000	20.715091000
H	0.587610000	0.109402000	12.644441000
H	-0.358039000	4.341470000	12.957060000

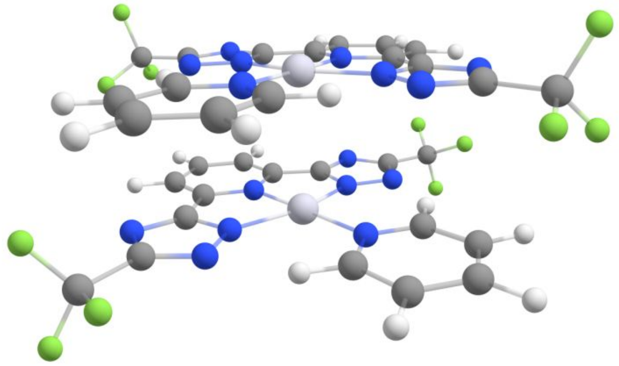

T_dimer-C
(Pt–Pt = 3.4238 Å)
SPE = -41381.92228 Hartree

Pt	-4.957215000	13.266397000	4.787412000
N	-4.916875000	11.771993000	3.519953000
N	-4.907597000	14.268101000	3.095675000
N	-4.881300000	14.204596000	0.841521000
N	-4.927808000	15.521050000	2.692025000
N	-4.988831000	11.723929000	6.042896000
N	-5.033614000	9.512451000	6.317794000
N	-5.036271000	11.522927000	7.346131000
N	-4.964376000	14.823668000	6.124471000
F	-5.545446000	16.464008000	-0.640409000
F	-3.643143000	17.004504000	0.193614000
F	-5.452693000	17.690800000	1.124491000
F	-3.957031000	8.899535000	9.062873000
F	-5.313885000	10.409023000	9.772896000
F	-6.071518000	8.613737000	8.860969000
C	-4.955127000	10.507905000	3.985590000
C	-4.966276000	9.430303000	3.106122000
H	-5.021271000	8.421572000	3.506379000
C	-4.934989000	9.684124000	1.707192000
H	-4.968583000	8.854914000	1.006591000
C	-4.902729000	10.988075000	1.244240000
H	-4.896738000	11.216314000	0.180747000
C	-4.898299000	12.056279000	2.164708000
C	-4.883854000	13.448062000	1.940910000
C	-4.916428000	15.432373000	1.351318000
C	-4.989402000	10.491557000	5.428214000
C	-5.064853000	10.194484000	7.467930000
C	-4.992489000	16.101862000	5.698788000

H	-4.998569000	16.262200000	4.620227000
C	-5.021851000	17.172793000	6.581382000
H	-5.077544000	18.182134000	6.182206000
C	-5.023152000	16.930987000	7.951189000
C	-4.983291000	15.609394000	8.384785000
H	-4.980211000	15.356540000	9.442637000
C	-4.958460000	14.583587000	7.450565000
H	-4.942333000	13.538006000	7.761497000
C	-4.890742000	16.669011000	0.494743000
C	-5.099696000	9.522278000	8.809719000
Pt	-8.379088000	13.280878000	4.672620000
N	-8.366336000	14.158347000	2.900290000
N	-8.376932000	15.211677000	5.221566000
N	-8.320784000	17.340154000	4.594678000
N	-8.369641000	15.915738000	6.340037000
N	-8.390643000	11.675494000	3.471148000
N	-8.352653000	10.877923000	1.397342000
N	-8.359835000	10.360204000	3.590439000
N	-8.432682000	12.373878000	6.497473000
F	-7.476622000	19.271595000	6.498749000
F	-8.013632000	17.942314000	8.104515000
F	-9.542229000	18.902698000	6.941343000
F	-7.684852000	8.213739000	0.884319000
F	-7.738753000	7.768099000	2.990270000
F	-9.567102000	7.983605000	1.887167000
C	-8.367619000	13.381550000	1.796109000
C	-8.343588000	13.972501000	0.535008000
H	-8.335257000	13.341552000	-0.349642000
C	-8.304758000	15.364646000	0.456388000
H	-8.257950000	15.847225000	-0.517293000
C	-8.301327000	16.148491000	1.614017000
H	-8.254165000	17.233494000	1.581308000
C	-8.337939000	15.502888000	2.847062000
C	-8.345988000	16.082370000	4.180173000
C	-8.330896000	17.180543000	5.924297000
C	-8.382106000	11.973861000	2.144748000
C	-8.333555000	9.920824000	2.332202000
C	-8.452011000	13.107470000	7.626671000
H	-8.450734000	14.192833000	7.507447000
C	-8.464835000	12.517746000	8.883534000
H	-8.476615000	13.155634000	9.764326000
C	-8.451654000	11.130315000	8.988938000
C	-8.443201000	10.379741000	7.817484000
H	-8.414113000	9.293693000	7.837806000
C	-8.432795000	11.030264000	6.591255000
H	-8.414841000	10.470390000	5.654074000
C	-8.343431000	18.335919000	6.878855000
C	-8.331397000	8.454396000	2.018291000
H	-5.067190000	17.753795000	8.662096000
H	-8.438140000	10.642316000	9.961812000

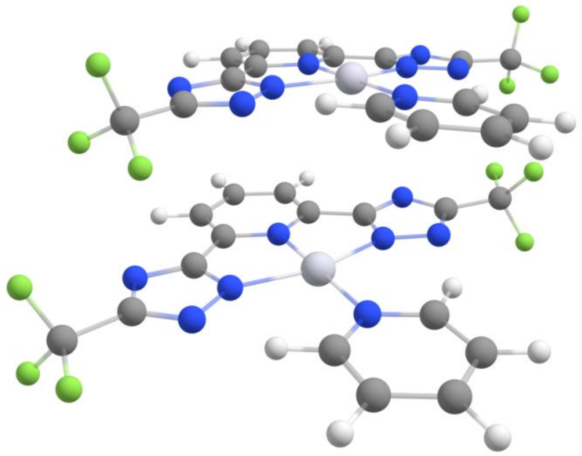

T_dimer-D
(Pt–Pt = 4.5849 Å)
SPE = -41381.90416 Hartree

Pt	-4.256675000	1.624139000	16.054666000
N	-5.570893000	0.577966000	17.098430000
N	-5.955131000	2.171642000	15.146488000
N	-6.327324000	2.954548000	14.150426000
N	-8.160535000	1.975468000	15.016213000
N	-3.031935000	0.679544000	17.339108000
N	-1.733398000	0.571475000	17.565026000
N	-2.824152000	-0.775638000	19.002918000
N	-2.913795000	2.727459000	14.995429000
C	-5.122410000	-0.206289000	18.095943000
C	-6.024833000	-0.955569000	18.845878000
H	-5.657456000	-1.588183000	19.649853000
C	-7.380896000	-0.871971000	18.521091000
H	-8.103417000	-1.455778000	19.088725000
C	-7.822655000	-0.054550000	17.479339000
H	-8.869631000	0.018478000	17.197813000
C	-6.872650000	0.677398000	16.771143000
C	-7.068992000	1.587935000	15.655503000
C	-7.649946000	2.811319000	14.102914000
C	-3.673895000	-0.137400000	18.210472000
C	-1.648922000	-0.308563000	18.562489000
C	-3.325760000	3.512160000	13.980969000
H	-4.396678000	3.510286000	13.760160000
C	-2.437742000	4.295369000	13.254749000
H	-2.821459000	4.913374000	12.446176000
C	-1.084940000	4.276192000	13.581243000
C	-0.668198000	3.460973000	14.630075000
H	0.375539000	3.408011000	14.931333000
C	-1.605870000	2.698831000	15.314802000
H	-1.315477000	2.040482000	16.136661000
C	-8.492828000	3.483578000	13.061099000
C	-0.319175000	-0.700796000	19.139223000
F	-9.527054000	4.102703000	13.616053000
F	-7.781893000	4.377265000	12.386332000
F	-8.970940000	2.597398000	12.195097000
F	0.250240000	0.322253000	19.768805000
F	0.522054000	-1.088154000	18.180701000
F	-0.455352000	-1.692843000	20.004629000
Pt	-3.597528000	-2.912723000	15.991850000
N	-4.974579000	-3.863769000	17.004784000
N	-5.214396000	-2.368736000	14.988965000
N	-5.514351000	-1.573915000	13.977157000

N	-7.438564000	-2.418101000	14.807575000
N	-2.478144000	-3.743218000	17.365653000
N	-1.213053000	-3.753790000	17.718301000
N	-2.367927000	-4.970742000	19.247050000
N	-2.162742000	-1.895436000	14.933588000
C	-4.597433000	-4.582489000	18.124555000
C	-5.575314000	-5.238502000	18.877618000
H	-5.264801000	-5.805472000	19.753219000
C	-6.915810000	-5.153189000	18.496921000
H	-7.683166000	-5.666821000	19.069447000
C	-7.271043000	-4.382877000	17.358309000
H	-8.304907000	-4.278152000	17.036395000
C	-6.277034000	-3.741054000	16.634634000
C	-6.400055000	-2.879580000	15.487205000
C	-6.844309000	-1.625034000	13.905649000
C	-3.195031000	-4.500541000	18.324447000
C	-1.192109000	-4.486177000	18.840832000
C	-2.490184000	-1.227450000	13.808640000

H	-3.538952000	-1.251237000	13.509466000
C	-1.549693000	-0.520441000	13.072991000
H	-1.870199000	-0.003273000	12.171389000
C	-0.226139000	-0.486047000	13.505321000
C	0.102421000	-1.167420000	14.672604000
H	1.112911000	-1.167125000	15.074114000
C	-0.885111000	-1.861207000	15.358938000
H	-0.653329000	-2.403817000	16.275190000
C	-7.582330000	-0.882275000	12.826481000
C	0.095611000	-4.778172000	19.563792000
F	-8.815031000	-0.602776000	13.199487000
F	-6.950538000	0.250460000	12.534688000
F	-7.637977000	-1.610570000	11.714002000
F	1.080554000	-4.034896000	19.089075000
F	0.423893000	-6.056854000	19.405878000
F	-0.033490000	-4.545351000	20.858952000
H	0.530045000	0.062217000	12.945483000
H	-0.370213000	4.886388000	13.030884000
